# Rational Design of a Self‐Assembling High Performance Organic Nanofluorophore for Intraoperative NIR‐II Image‐Guided Tumor Resection of Oral Cancer

**DOI:** 10.1002/advs.202206435

**Published:** 2023-01-31

**Authors:** Xianwei Sun, Praveen Kumar Chintakunta, Andrew A. Badachhape, Rohan Bhavane, Huan‐Jui Lee, David S. Yang, Zbigniew Starosolski, Ketan B. Ghaghada, Peter G. Vekilov, Ananth V. Annapragada, Eric A. Tanifum

**Affiliations:** ^1^ Department of Radiology Baylor College of Medicine Houston TX 77030 USA; ^2^ Department of Radiology Texas Children's Hospital Houston TX 77030 USA; ^3^ Department of Chemical and Biomolecular Engineering University of Houston Houston TX 77204 USA; ^4^ Department of Chemistry University of Houston Houston TX 77204 USA; ^5^ Present address: Sai Life Sciences Ltd Turakapally Telangana India

**Keywords:** intraoperative imaging, mesoscopic solute‐rich clusters, NIR‐II margin delineation, NIR‐II probe, organic nanofluorophore, self‐assembling

## Abstract

The first line of treatment for most solid tumors is surgical resection of the primary tumor with adequate negative margins. Incomplete tumor resections with positive margins account for over 75% of local recurrences and the development of distant metastases. In cases of oral cavity squamous cell carcinoma (OSCC), the rate of successful tumor removal with adequate margins is just 50–75%. Advanced real‐time imaging methods that improve the detection of tumor margins can help improve success rates,overall safety, and reduce the cost. Fluorescence imaging in the second near‐infrared (NIR‐II) window has the potential to revolutionize the field due to its high spatial resolution, low background signal, and deep tissue penetration properties, but NIR‐II dyes with adequate in vivo performance and safety profiles are scarce. A novel NIR‐II fluorophore, XW‐03‐66, with a fluorescence quantum yield (QY) of 6.0% in aqueous media is reported. XW‐03‐66 self‐assembles into nanoparticles (≈80 nm) and has a systemic circulation half‐life (*t*_1/2_) of 11.3 h. In mouse models of human papillomavirus (HPV)+ and HPV‐ OSCC, XW‐03‐66 outperformed indocyanine green (ICG), a clinically available NIR dye, and enabled intraoperative NIR‐II image‐guided resection of the tumor and adjacent draining lymph node with negative margins. In vitro and in vivo toxicity assessments revealed minimal safety concerns for in vivo applications.

## Graphical abstract

A new NIR‐II fluorophore, XW‐03‐66, self‐assembles into nanoparticles with 80 nm hydrodynamic diameter in aqueous media, generating NIR‐II images with 6% fluorescence quantum yield. When administered intravenously, the particles accumulate in mouse tumor models of oral cancer enabling real‐time NIR‐II image‐guided resection of tumors and draining lymph nodes with negative resection margins. Preliminary toxicity evaluation shows very mild concerns.

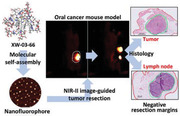

## 1 Introduction

Oral cavity cancers rank among the top 10 solid tumors worldwide, with an annual incidence of 350 000. About 90% of these cancers are oral cavity squamous cell carcinoma (OSCC), affecting sites in the oral mucosa around the tongue and floor of the mouth. ^[^ ^1^ ^]^ The prognosis for OSCC is poor, with a five‐year survival rate of just 50– 64.8%.^[^ ^2^, ^3^, ^4^ ^]^ Although standard treatment is a combination of surgery, radiation, and chemotherapy, recent reports suggest that surgery with adequate resection margins (> 5 mm) leads to higher survival and a reduction in local recurrence rates. However, adequate resections are reported in only 50–75% of cases worldwide.^[^ ^5^, ^6^, ^7^, ^8^ ^]^ These poor results have been attributed to the complex anatomy of the oral cavity and the lack of effective intraoperative guidance. Currently, surgeons rely primarily on physical inspection, palpation, and preoperative imaging to determine resection margins. To improve outcomes, protocols in which resection margins are determined through intricate tissue tagging and sample collection by a team of surgeons and pathologists during surgery or the frozen section intraoperative histopathologic approach have been implemented.^[^ ^9^, ^10^, ^11^ ^]^ However, these have not resulted in a significant impact on regional control or survival rates, and come at a high cost.^[^ ^7^, ^9^, ^12^, ^13^ ^]^ Intraoperative image‐guided resection can significantly simplify tumor margin delineation, reduce surgical staff, and improve adequate resection outcomes. While cross‐sectional imaging techniques such as magnetic resonance imaging (MRI) and computed tomography can be effective preoperative imaging tools for surgical planning in OSCC cases, they are less effective for intraoperative procedures in the oral cavity due to its complicated anatomy. Intraoperative ultrasound can also effectively delineate margins for some tumor types, but several drawbacks, including image quality, ultrasound artifacts, and patient positioning, limit its broad applicability.^[^ ^14^ ^]^

A more promising imaging modality for real‐time interrogation and guidance in surgical procedures is fluorescence image‐guided tumor surgery, which uses dyes that fluoresce in the visible and the first near‐infrared (NIR‐I) window (400–900 nm).^[^ ^15^, ^16^, ^17^, ^18^, ^19^ ^]^ The leading NIR‐I dye is indocyanine green (ICG), a small organic molecule approved by the US Food and Drug Administration and the European Medicines Agency, which has been used successfully in several research studies and clinical procedures.^[^ ^16^, ^20^, ^21^, ^22^ ^]^ The performance of some nanoparticle‐based NIR‐I dyes, albeit still in clinical trials, further corroborates the utility of this technique for intraoperative image‐guided tumor resection with improved negative margins.^[^ ^16^, ^17^ ^]^ However, imaging in the NIR‐I window is limited by tissue auto‐fluorescence and low tissue penetration due to tissue absorption and scattering. These limitations are significantly reduced in the second near‐infrared (NIR‐II) window (1000–1700 nm) where scattering, tissue absorption, and auto‐fluorescence are minimal and NIR‐II dyes generate superior images with high signal‐to‐background ratio (SBR), at depths of up to 3 cm, and spatial resolution of ≈25 µm.^[^ ^23^, ^24^, ^25^, ^26^, ^27^ ^]^

That said, finding the ideal NIR‐II dye has proven challenging. Although ICG fluoresces in the NIR‐II window,^[^ ^28^ ^]^ its emission maximum is in the NIR‐I window and the NIR‐II fluorescence originates from a weak tail of the emission spectrum which stretches into the NIR‐II region. Its NIR‐II QY is reported at 0.042% in PBS,^[^ ^29^ ^]^ and image quality is suboptimal. In addition, ICG has a short blood circulation half‐life, as 97% is removed from circulation via the liver and excreted through the biliary route, without biotransformation, in 20 min post‐intravenous injection in healthy individuals.^[^ ^30^ ^]^ This significantly limits ICG's usefulness in tumor margin delineation, except in primary liver tumors where prolonged retention of ICG by malignant cells enables real‐time identification of liver tumors.^[^ ^21^ ^]^ There is an ongoing effort to develop nanoparticle variants of ICG with longer blood circulation times and tumor localization (some in clinical trials),^[^ ^16^ ^]^ but dyes with emission maxima in the NIR‐II window are more desirable.

A variety of NIR‐II fluorophores based on different molecular constructs, including small organic molecules; conjugated organic polymers; and inorganic nanomaterials such as single‐walled carbon nanotubes, quantum dots, and rare earth nanomaterials, have been prepared and tested in vitro and in pre‐clinical settings with several outstanding results.^[^ ^24^ ^]^ However, the clinical translation of each of these materials as imaging probes for intraoperative tumor surgery remains challenging for a variety of reasons. While most of the small organic molecules are generally biocompatible and present few safety concerns, they often show poor in vivo performance (low fluorescence quantum yield, low photostability, and low tumor specificity). On the other hand, polymeric organic nanoparticles and inorganic nanomaterials often show high in vivo fluorescence and can accumulate in tumors either by passive (enhanced permeation and retention effect, EPR)^[^ ^31^ ^]^ or active ligand targeting mechanisms. Their biosafety remains a serious concern due to their slow excretion kinetics and long‐term in vivo retention.^[^ ^32^ ^]^ The goal, therefore, is finding novel NIR‐II constructs that combine the safety profile of small organic molecules and the high in vivo fluorescence performance and tunable imaging functionality of organic polymers and inorganic nanomaterials.

The source of NIR‐II fluorescence in small organic molecule fluorophores is the characteristic huge *π*‐conjugated system. This enables an extensive *π*‐electron flux through the whole system, lowering the energy bandgap of electron transitions between the highest occupied and lowest unoccupied orbitals, achieving longer wavelength fluorescence. The overall intensity of the fluorescence release upon excitation is influenced by energy exchange with the environment of the molecule as it returns to the ground state. In solutions, some of the energy is transferred to the solvent as the fluorophore interacts with the solvent molecules, resulting in solvent‐induced fluorescence quenching. In organic solvents, such as toluene at low solute concentrations, energy transfer between the highly hydrophobic fluorophore and solvent molecules is minimal and such solutions generate fluorescence with high quantum yield. At high concentrations, the high planarity of the fluorophore may drive п‐stacking, causing the molecules to aggregate. This results in aggregation‐induced quenching and low overall fluorescence output. In aqueous media, both solvent‐ and aggregation‐induced quenching effects on the fluorophore are exacerbated by the high polarity of water molecules, resulting in poor fluorescence performance (low QY), and rendering them unsuitable for in vivo applications. Furthermore, the hydrophobicity of organic fluorophores renders both in vivo safety and biodistribution unpredictable.

**Figure 1:**
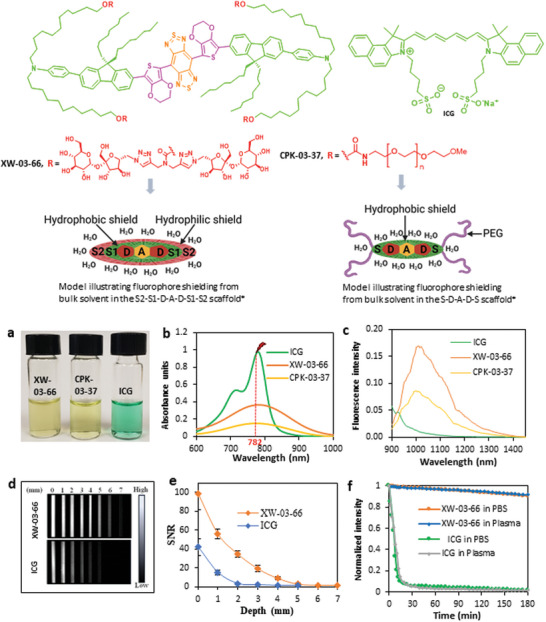
Structures and spectroscopic properties of CPK‐03‐37 and XW‐03‐66, compared to IGG. a) Solutions of compounds and ICG in PBS (50 µM); b) Absorption spectra of compounds and ICG in PBS; c) Emission spectra of compounds and ICG in PBS; d) NIR‐II images (excitation 785 nm, 1300 nm long‐pass filter) of capillary tubes through different depths of 1% Intralipid; e) Plot of SNR against imaging depth; f) Comparison of the photostability of XW‐03‐66 versus ICG in PBS and plasma, under continuous laser excitation (settings: 475 mA and 2V, 400 mW optical power, 55 mW cm^−2^ power density on imaging stage) for 3 h shows a drop in fluorescence to baseline levels within 30 min for ICG while that of XW‐03‐66 stays above 90% over the 3 h period in both media. *Created with BioRender.com.

Ionic moieties, such as sulphonate and carboxylate groups, and polyethylene glycol (PEG) chains have been used to hydrophilize these molecules, but PEG chains are mostly preferred due to their electrical neutrality and biocompatibility. In the past decade, research on small organic NIR‐II fluorophores has focused mostly on molecular constructs which limit the attack of water molecules on the fluorophore backbone and minimize aggregation potential. The shielded donor‐acceptor‐donor (S‐D‐A‐D‐S) scaffold engineered and optimized by the Dai group^[^ ^33^, ^34^ ^]^ has been tremendously successful in addressing both limitations. However, a closer look at the lead performers in the series, such as IR‐FEP (QY = 2.0% in water),^[^ ^33^ ^]^ suggests even more effective shielding is possible. Literature reports on PEGylated nanoparticles suggest that PEG chains (as used in hydrophilizing IR‐FEP) maintain a more stretched than curled conformation in an aqueous solution, thereby exposing the entire fluorophore to the bulk solvent.^[^ ^35^ ^]^ We hypothesized that extending the hydrophobic shield around the S‐D‐A‐D‐S scaffold and solubilizing the construct by expressing evenly distributed smaller hydrophilic moieties, such as simple sugars instead of PEG chains, will result in a double‐shielded construct: the fluorophore (D‐A‐D) at the core, the hydrophobic shield (S1) and a second shield (S2) from bulk solvent, by a hydrophilic sugar envelope, stabilized by a network of intramolecular hydrogen bonding (H‐bonding) between the sugar moieties and water molecules. This would result in a novel shield 2‐shield 1‐donor‐acceptor‐donor‐shield 1‐shield 2 (S2‐S1‐D‐A‐D‐S1‐S2) NIR‐II molecular scaffold (**Figure** **1**). In addition, at a suitable solute concentration, intermolecular H‐bonding between hydroxyl groups on different molecules can drive self‐assembly and nanocluster formation.

In this report, we verify this hypothesis through the design, synthesis, characterization, and evaluation of a novel PEGylated S‐D‐A‐D‐S‐type NIR‐II fluorophore, CPK‐03‐37, and its sucrose labeled S2‐S1‐D‐A‐D‐S1‐S2 variant, XW‐03‐66. Our data show that replacing PEG in CPK‐03‐37 (QY = 3.4) with sucrose as the solubilizing moiety in XW‐03‐66 (QY = 6.0) results in an almost two‐fold increase in the QY. Furthermore, XW‐03‐66 self‐assembles into mesoscopic solute‐rich clusters in aqueous media with a particle hydrodynamic diameter of 80 ± 5 nm. This unique characteristic gives the construct a long systemic circulation half‐life (≈ 11 h), enabling high‐resolution NIR‐II imaging of the vasculature for prolonged periods (up to 6 h post‐administration). In addition, it allows the probe to accumulate in solid tumors via the EPR effect, enabling tumor imaging and real‐time NIR‐II image‐guided resection of tumors with negative margins. In vitro assessments of cytotoxicity in seven different cell lines, and inflammatory potential in three key immune cell lines, suggest that XW‐03‐66 poses minimal biosafety concerns. These in vitro safety observations are corroborated by acute and long‐term in vivo safety data, including serum chemistry and histopathological analyses of liver and spleen tissue samples.

## 2 Results and Discussion

### 2.1 Molecular Design, Synthesis, and Characterization of XW‐03‐66

Two key factors that influence the in vivo fluorescence performance of small organic molecule dyes are solvent‐ and aggregation‐induced fluorescence quenching due to different interactions with highly polar water molecules. Since the discovery of the first NIR‐II dyes, synthetic chemists have primarily focused on designing new molecules with optimal in vivo fluorescence performance and imaging function.^[^ ^24^, ^25^, ^30^ ^]^ The S‐D‐A‐D‐S system incorporates appropriate features to reduce both solvent‐ and aggregation‐induced fluorescence quenchings.^[^ ^33^, ^34^, ^36^ ^]^ For example, in the design of IR‐FEP,^[^ ^33^ ^]^ one of the best performers in this series, using 3,4‐ethylenedioxythiophene (EDOT) as the donor unit afforded a conformational distortion of the conjugated backbone, thereby limiting aggregation‐induced quenching. Alkyl chains on the fluorene shielding unit stretch out of the plane of the conjugated backbone, further limiting the propensity of the molecule to aggregate while also serving as a solvent shield for the core. We adapted this scaffold as the basis for our molecular design in which EDOT and benzobisthiadiazole were maintained as the donor and acceptor units, respectively. To improve the shielding effect of the unit, the alkyl chains on the fluorene were extended from six to eight carbon atoms each. We also appended to each of the fluorene‐shielding units, an *N,N*‐dialkylaniline moiety bearing functionalized C_11_ chains as anchors to the solubilizing moieties. Molecular dynamics studies on dilute solutions of sucrose suggest the existence of two hydration shells between the solute and the bulk solvent.^[^ ^37^, ^38^ ^]^ The first hydration shell spans 2.8–3.7 Å from the solute hydroxyl oxygens, with strong radial water structuring. This structuring is stabilized by inter‐ and intramolecular hydrogen bonding between water and solute hydroxy groups, with each hydroxyl group surrounded by 3.9 to 4.4 nearest neighbors, indicating saturated hydrogen bonding capacity. At the limit of the first hydration shell (3.7 Å), the water density is estimated to decrease to a little more than half of the bulk water density. The second hydration shell has a long‐range structure with a center of 5.5 Å. Taken together, both hydration shells form a substantial shielding effect between the core of the solute and the surrounding bulk water molecules. We reasoned that this shielding phenomenon can be adapted to the S‐D‐A‐D‐S system. The PEGylated variant of the molecule CPK‐03‐37 was accessed in eight linear synthetic steps (Scheme S1.1, Supporting Information). To access the sucrose derivative XW‐03‐66, 6f‐azido sucrose was synthesized in five linear synthetic steps and appended to the core structure using click chemistry (Schemes S1.2 and S1.3, Supporting Information). All intermediates and the final structure were characterized by ^1^H and ^13^C NMR, and mass analysis (HRMS or MALDI where appropriate).

### 2.2 Optical Properties of XW‐03‐66

Like ICG, both compounds dissolve in PBS resulting in clear solutions (Figure 1a). The CPK‐03‐37 solution shows an absorbance spectrum centered at 786 nm while the XW‐03‐66 solution is centered at 796 nm (Figure 1b). Excitation of each solution with a 782 nm laser generates emissions centered at 1018 nm for XW‐03‐66 and 1010 nm for CPK‐03‐37, each with a tail that extends beyond 1300 nm and QY of 6.0% and 3.4%, respectively. The subtle red shift in absorption and emission maxima from CPK‐03‐37 to XW‐03‐66 is expected because there is no change in the electronics of the core fluorophore. However, the near doubling of the fluorescence quantum yield (Figure 1c) suggests a significant change in the immediate environment of the fluorophore, with the switch from PEG units as the hydrophilic moieties to sucrose units. Given its superior optical performance, we focused our further in vitro and in vivo evaluations on XW‐03‐66, in comparison with clinically approved ICG. NIR‐II images at increasing depths (Figure 1d) of capillary tubes filled with equimolar solutions of XW‐03‐66 or ICG, immersed in a phantom consisting of 1% Intralipid solution, show that tubes containing XW‐03‐66 can be clearly visualized up to a depth of 6 mm, while ICG is undetectable at depths beyond 3 mm. A plot of the SBR versus depth (Figure 1e) highlights the superiority of XW‐03‐66 over ICG in the NIR‐II window at increasing depths. Continuous laser excitation (55 Wcm^−2^, 785 nm) of XW‐03‐66 or ICG solutions in both PBS and bovine plasma for 3 h (Figure 1f) shows that XW‐03‐66 is highly photostable in both media. ICG, in contrast, shows a sharp drop in fluorescence to the baseline within 30 min of illumination. Thus, in in vivo applications, procedures lasting several hours may not require continuous bolus administration of XW‐03‐66. Further evaluation of the PBS solution reveals a fundamental property of XW‐03‐66: self‐assembly into unique nanoscale mesoscopic aggregates referred to as solute‐rich clusters in the literature,^[^ ^39^, ^40^ ^]^ which may contribute to its in vivo properties. This characteristic is not observed with CPK‐03‐37 at low concentrations (< 500 µM), but some less organized particles with a hydrodynamic diameter of 6.6 nm are observed at a concentration of 500 µM (Figure S2.30, Supporting Information). To further assess this intriguing behavior, we performed molecular dynamic simulations on both compounds to gain more insights on their respective conformations in solution.

### 2.3 Conformations of CPK‐03‐37 and XW‐03‐66 in Solution

**Figure 2:**
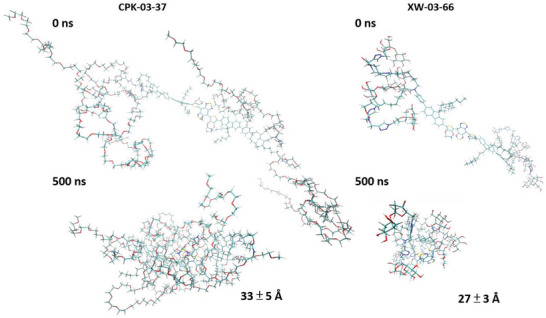
Conformations of the CPK‐03‐37 and XW‐03‐66 dyes at the start of all‐atom MD simulations and after 500 ns of evolution in explicit solvent comprised of 0.15 solution of NaCl in water. C atoms are shown in teal, H, in silver, O, in red, N, in cobalt, and S, in gold. The diameters of both final structures were computed as the averages of six measurements of the distances between atoms that were furthest apart along uniformly azimuthally distributed axes that pass through the molecular center of mass and are shown at the bottom.

The suspected irregular shapes and the structural flexibilities of the two dye molecules would hamper experimental determinations of their conformation using X‐ray crystallography or solution NMR. To fill this gap, we employed all‐atom molecular dynamics simulations of the evolution of the dye structures in an atomically explicit solvent consisting of 0.15 M NaCl dissolved in water. In the starting conformation, at 0 ns, the common fluorophore core of the two dyes (Figure 1) was assumed to be linear (**Figure** **2**). Random starting conformations were assumed for the side aliphatic and PEG chains and sugar moieties.

Within 100 ns of structure evolution, the eight aliphatic chains attached to the periphery of the fluorophore have collapsed on top of the core (Figure S1.1c,d, Supporting Information), likely driven by hydrophobic attraction. The sugar residues of XW‐03‐66 fold within about the same time (Figure S1.1d, Supporting Information) and the variation of the XW‐03‐66 conformation in the remaining 400 ns of simulation is minor (Figure S1.1b,c, and Movie S3.1, Supporting Information). By contrast, the PEG chains on the surface of CPK‐03‐37 fold slower (Figure. S1.1.c, Supporting Information) and even after 500 ns one of them still extends into the solution (Figure S1.1c and Movie S3.2, Supporting Information). The distinct folding rates of the side chains at the periphery of the two dyes reflect in the evolutions of the root mean squared deviation (RMSD) of the position of the atoms from those in the starting conformations. The rapid folding of the aliphatic tethers of both dyes results in a jump of RMSD to about 20 Å within the first 20 ns of simulation time. After that time, the RMSD of XW‐03‐66 remains relatively steady and fluctuates around 20 Å likely owing to the conformational flexibility of the side chains. For CPK‐03‐37 RMSD continuously increases to 30 Å as the side PEG chains fold around the hydrophobic core. Notably, the larger PEG chains and the remaining partially unfolded chain on the surface of CPK‐03‐37 contribute to a larger size of the folded molecule, 33 ± 5 Å, compared to 27 ± 3 Å for XW‐03‐66 (Figure 2).

The final simulated structures, at 500 ns, reveal another important distinction between CPK‐03‐37 and XW‐03‐66. The PEG and the underlying aliphatic chains appear relatively uniformly distributed around the fluorophore core (Figure 2 and Movie S3.2, Supporting Information), protecting the core from contact with water and its impact on fluorescence. In contradistinction, the positions of the aliphatic tethers and the sugar residues on the surface of the 500 ns conformation of XW‐03‐66 (Figure 2 and Movie S3.1, Supporting Information) leave channels for free access of the solvent to the fluorophore core and the hydrophobic moieties. This observation appears to refute our hypothesis that the fluorescence performance of XW‐03‐66 is better than that of CPK‐03‐37 owing to improved protection from solvent interactions by the sugar residues. We posit the main contribution of the superior fluorescence of XW‐03‐66 is the nanoparticle formation driven by hydrophobic interactions via the exposed hydrophobic pockets and stabilized by intramolecular hydrogen bonding between sucrose moieties once formed. In CPK‐03‐37, on the other hand, the complete PEG envelope around the fluorophore and hydrophobic moieties leaves no room for any strong intermolecular hydrophobic interactions or hydrogen bonding and therefore, less propensity to form particles.

### 2.4 Characterization of XW‐03‐66 Nanoparticles

**Figure 3:**
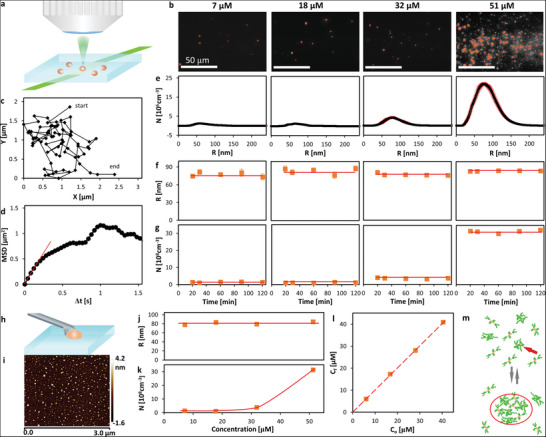
Characterization of self‐assembled XW‐03‐66 nanoparticles. a) Schematic of oblique illumination microscopy (OIM). A 500 mm thick solution layer is illuminated by a green laser (wavelength 532 nm) at an oblique angle. Upward scattered light is collected by a microscope lens; b) Representative OIM micrographs of aggregates in XW‐03‐66 solutions at 37 ⁰C at the concentrations indicated above the images. The clusters appear as red speckles; c) Trajectory of a cluster determined from a sequence of OIM images as in b recorded at 25 frames s^−1^; d) Mean squared displacement, MSD, calculated from the trajectory in b as a function of the lag time Δt; e) Number density N distribution of the radii *R* of clusters determined by OIM at 37 ⁰C and the concentrations indicated at the top of each column in (b). The averages of five measurements are displayed. The error bars represent the respective standard deviations; f,g) Evolutions of the average radius *R*, in (f), and number *N* of clusters per unit solution volume, in (g), at 37 ⁰C in solutions with concentrations indicated at the top of each column in (b) determined by OIM from images as in (b). The averages of five measurements are displayed. The respective standard deviations are, in several cases, smaller than the symbol size. Horizontal lines denote the mean values of *R* and *N*; h) Schematic of interaction of the AFM tip with a cluster on a substrate highlighting that the angle of the AFM tip is smaller than the angle between the cluster surface and the substrate. This angle ratio allows accurate determination of the cluster sizes; i) An AFM image of XW‐03‐66 clusters deposited on a glass substrate. Cluster height is displayed as color according to the scale bar at the right; j,k) The concentration dependences of *R* and *N* determined at 37 ⁰C by OIM. The averages of five measurements are displayed. The error bars represent the respective standard deviations and are smaller than the symbol size for most data points. Horizontal line in (j) denotes the mean value of *R*; the curve in (k) is a guide to the eye; l) The concentration *C*_f_ of the solution after incubation for 20 min at 37 °C and removal of the clusters by filtration as a function of the initial solution concentration *C*_0_. Dashed line corresponds to *C*_f_ = *C*_0_; m) Schematic of formation of mesoscopic XW‐03‐66‐rich clusters, highlighted in an oval, owing to the accumulation of XW‐03‐66 solute dimers, indicated with a red arrow.

XW‐03‐66 was dissolved in PBS and the solution was filtered through a membrane with 220 nm pores, to remove extrinsic inhomogeneities, and examined within 10–20 min of preparation. The resulting aggregates in the solution were monitored with oblique illumination microscopy (OIM, **Figure** **3**a). In this method, the solution is held in a flat cuvette and illuminated by a 532 nm laser. The scattered light is recorded by a CCD camera attached to a microscope. In the Rayleigh scattering regime (particle diameter << light wavelength), in which we operate, the scattered light intensity scales as the sixth power of the particle size (notably, in the Mie regime (particle diameter ∼> wavelength), the scattered intensity is well represented by a Ricatti–Bessel function). Owing to the Rayleigh scaling, scattered light intensity is dominated by aggregates and not by individual molecules. Each aggregate is treated as a point source of scattered light and its location is determined from the OIM images (Figure 3b). The Brownian trajectories of individual aggregates are tracked from sequences of images collected at 25 Hz (Figure 3c). The diffusion coefficients of the individual clusters are computed from the trajectories by correlating the mean squared displacements of individual clusters with the lag time (Figure 3d).^[^ ^41^ ^]^ The cluster sizes are then evaluated using the Stokes–Einstein equation and the known viscosity of the buffer. This procedure allows OIM to assess sizes as low as 20 nm, much smaller than the diffraction limit of a conventional optical microscope.^[^ ^39^, ^40^, ^42^, ^43^, ^44^ ^]^ 3e. The clusters observed in solutions of XW‐03‐66 exhibit relatively narrow size distributions (Figure 3e) with an average *R* = 80 ± 5 nm. Such clusters would hold ≈20000 moderately packed XW‐03‐66 molecules with a molecular weight of 5526 g mol^−1^ and a diameter of ≈3 nm (Figure 2). Both *R* and *N* are steady for at least 2 h (Figure 3f,g), behaviors that stand in contrast to expectations for crystals or other solid or liquid aggregates that result from first‐order phase transitions,^[^ ^45^, ^46^, ^47^ ^]^ for which nucleation of new liquid domains and their growth persists and *R* and *N* increase in time.^[^ ^48^, ^49^ ^]^ The particle size distribution is corroborated by cryogenic electron microscopic images of a sample from a 50 µM solution of XW‐03‐66 (Figure S1.2, Supporting Information), which shows a particle distribution consistent with the OIM data.

To complement the characterization of the aggregates’ sizes and properties, we used atomic force microscopy (AFM, Figure 3h,i). The aggregates were deposited on a glass substrate and probed with a pyramid‐shaped SiC tip attached to a flexible cantilever. The modification of the cantilever oscillations due to interactions with the substrate and the aggregates was recorded and analyzed to recover the aggregates’ shapes.^[^ ^50^, ^51^, ^52^ ^]^ The AFM images reveal that the aggregates deposited on the glass substrate are shaped as domes with circular bases with diameters of ≈100 nm and heights of ≈5 nm (Figure 3i). With these dimensions, the angle between the cluster surface and the substrate is less than 5^o^. This angle is smaller than the 45^o^ angle at the apex of the AFM tip and allows the tip to approach the cluster base and accurately convey the cluster diameter at the base (Figure 3h). These aggregates’ volumes are smaller than those of the aggregates observed by OIM, likely owing to solvent evaporation after exposure to air before AFM imaging. The shrinking aggregate size indicates that the aggregates entrap a substantial volume of the solution. The solvent evaporation, on the other hand, boosted the aggregates’ viscosity and enabled imaging by probing with the AFM tip. The AFM images reveal two important characteristics of the aggregates. First, their size distribution (Figure 3i) is narrow, consistent with the OIM measurements (Figure 3e). Second, the aggregates’ shared circular cross‐section (Figure 3i) suggests that prior to deposition they were spherical, a shape that minimizes their surface free energy and is only possible for liquid objects.

The reversibility of the aggregates is affirmed by the correlations of *R* and *N* with the molecule concentration (Figure 3j,k). The concentration *N* declines from 30 × 10^8^ to 2 × 10^8^ cm^−3^, ≈15‐fold, in response to a three‐fold reduction of concentration, from 51 to 17 µM (Figure 3k). *R* is consistently ≈80 nm (Figure 3j). The exaggerated response of *N* to reduced concentration indicates that the aggregates are not irreversibly disordered agglomerates, whose concentration is diluted in parallel with that of the molecule, but rather condensates existing in dynamic equilibrium with the host solution.

Two other unusual behaviors of the aggregates are the low fraction of the solute they capture and their lack of solubility. We measured *C_f_*, the concentration of equilibrium between the aggregates and the solution, by filtering out the aggregates after incubation for ≈1 h. The equilibrium concentration *C*_f_ is approximately equal to the initial *C*_0_ (Figure 3l), demonstrating that the aggregates capture a minor fraction of the solute, in accordance with the low fraction of the solution volume they occupy: $\phi_{2}\approx \frac{4}{3}\pi R^{3}N$ ≈ 10^−5^ at the highest solute concentration examined, 51 µM, and even lower at the lower XW‐03‐66 concentrations. Surprisingly, *C*_f_ is not constant but instead increases with *C*_0_ (Figure 3l). The finding of variable equilibrium concentration is in striking contrast with expectations for phase equilibria between solutions and crystals, amorphous aggregates, and liquids. These phases equilibrate with solutions of concentration that are constant and independent of the initial concentration of the solution in which they form.^[^ ^47^, ^53^, ^54^, ^55^ ^]^

The XW‐03‐66 aggregates’ behaviors deviate from thermodynamic predictions for domains of new solids or liquids that result from first‐order phase transitions. However, they do cohere with previous observations of mesoscopic solute‐rich clusters of proteins^[^ ^41^, ^44^, ^56^ ^]^ and organic molecules.^[^ ^57^, ^58^ ^]^ According to recent models, the mesoscopic clusters form due to the accumulation of transient oligomers (Figure 3m, where the transient oligomers are tentatively represented as dimers).^[^ ^44^, ^59^ ^]^ In the clusters, the transient oligomers co‐exist with monomers.^[^ ^60^, ^61^ ^]^ This kinetic model accounts for the conversion of monomers to oligomers, the diffusion of monomers to fill the void created by this conversion, as well as the outflow and decay of the transient oligomers.^[^ ^59^, ^62^, ^63^ ^]^ The cluster size appears as a square root of the product of the diffusivity of the oligomers and their lifetime and is, hence, independent of the solute concentration and steady in time.^[^ ^59^, ^62^, ^63^ ^]^ By contrast, the amount of solute captured in the clusters, and the related number of clusters and cluster population volume, increase exponentially with the solute concentration due to the equilibrium between the clusters and the bulk solution.^[^ ^59^, ^63^, ^64^ ^]^ A thermodynamic model of this equilibrium, comprised of concurrent chemical and phase transformations, predicts a strong correlation between the final *C_f_* and the initial *C_0_* solution concentrations. The mesoscopic aggregates of XW‐03‐66 appear to comply with the predictions of this model remarkably well. We, therefore, conclude that the aggregates are mesoscopic XW‐03‐66‐rich clusters.

### 2.5 In Vivo Pharmacokinetics and Biodistribution

**Figure 4:**
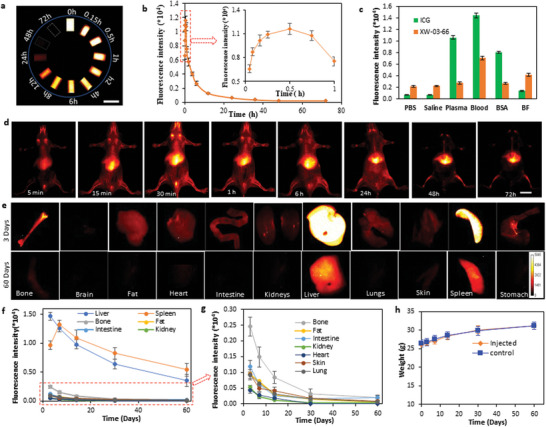
Pharmacokinetics and biodistribution of XW‐03‐66. a) NIR‐II images of capillary tubes containing blood drawn at different time points post‐injection of XW‐03‐66 in mice C57BL/6J mice (*n* = 4, scale bar = 6 mm); b) Plot of blood fluorescence intensity against time showed a bi‐phasic pharmacokinetic distribution curve: an equilibration phase characterized by an increase in fluorescence intensity within the first 30 min, followed by an elimination phase with a half‐life of 11.3 h; c) Evaluation of changes in fluorescence intensity upon exposure of XW‐03‐66 (10 µM) to plasma proteins, including bovine serum albumin (BSA) and bovine fibrinogen (BF), suggests that observed increase in fluorescence upon mixing with blood is due to interactions with fibrinogen; d) In vivo NIR‐II imaging in nude mice (*n* = 4) suggests that although some of the dye is cleared via the urinary system, the major clearance pathway is the monocyte phagocyte system (MPS) as the liver, spleen, and bone show high accumulation of the dye (scale bar = 8 mm); e) Organs harvested from treated mice over a 60‐day period show noteworthy accumulation in organs other than the liver, spleen, and bone at 3 days post‐injection, but cleared by day 60; f,g) A plot of fluorescence intensity against time between 3 and 60 days shows that fluorescence signal in all organs except the liver and spleen is back to baseline levels at day 60. The low residual signal observed in the bone and intestine at day 60 is consistent with the MPS elimination pathway; h) Weight curves for treated animals and untreated controls over the 60‐day period show no significant difference between the test and controls.

To investigate the in vivo pharmacokinetics and biodistribution, XW‐03‐66 (1000 µg mL^−1^ in PBS, 5 mg kg^−1^) was administered intravenously (i.v.) via the tail vein in C57BL/6J mice (*n* = 4). Analysis of NIR‐II fluorescence (excitation at 785 nm and imaged using 1300 nm long pass filter) of venous blood samples collected at multiple time points post‐injection (**Figure** **4**a) showed that upon intravenous administration, the fluorescence of XW‐03‐66 increases, peaking at about 30 min post‐injection, before gradually reducing and returning to baseline levels after 72 h (Figure 4b). This data shows a systemic circulation half‐life (*t*_1/2_) of ≈11.3 h, comparable to the *t*_1/2_ of recently reported NIR‐II smart self‐assembled amphiphilic cyclopeptide‐dye, SIMM1000, (12.9 h)^[^ ^65^ ^]^ but shorter than the NIR‐II polymeric organic fluorophore, p‐FE (*t*_1/2_ ∼16 h).^[^ ^66^ ^]^ Nude mice (*n* = 4) injected with the same dose of the agent showed a similar pharmacokinetic profile. NIR‐II imaging of the head, back, abdomen, and hindlimb at various time points shows clear visualization of the vasculature in these areas for up to 6 h post‐injection (Figures S1.3 and S1.4, Supporting Information). Whole body imaging shows a signal in the bladder within the first 6 h and a majority showing up in the liver, increasing with time over the 72‐h period, during which the signal persists in the blood. Superficial cervical lymph nodes are clearly visible and increase in signal intensity by 5 min post‐injection. The signal appears in the bones at about 12 h post‐injection and becomes increasingly prominent in the sternum and limbs as the blood pool signal diminishes. Postmortem ex vivo NIR‐II images of tissue and organs (bone, fat, heart, intestine, kidney, liver, lung, skin, and stomach) collected at 72 h post‐injection from treated animals (blood fluorescence back to baseline levels) show low levels of fluorescence attributed to the dye in target organs and saturating signal in the liver and spleen (Figure 4d). Analysis of tissues harvested at 60 days post‐treatment shows a return of signal to baseline levels for most organs, except bone, liver, and spleen (Figure 4e). Taken together, this data suggests that the main clearance pathway for this dye is the mononuclear phagocyte system (MPS),^[^ ^67^, ^68^ ^]^ consistent with nanoparticle clearance.^[^ ^69^ ^]^

Fluorescent signal enhancement upon mixing with blood and solutions of some macromolecules has been reported for several NIR‐II dyes in the literature. For instance, ICG exhibits an increase in fluorescence upon mixing with blood because of binding with serum albumin, which has been shown to increase the fluorescence by a factor of 23 in solution, compared to a solution of ICG in PBS. Another NIR‐II dye, LZ‐1105,^[^ ^70^ ^]^ also exhibits an increase in fluorescence upon mixing with blood, but in this case due to binding to fibrinogen. To assess which macromolecule is responsible for the observed XW‐03‐66 in vivo fluorescence enhancement, solutions of the dye were prepared in PBS, saline, reconstituted bovine plasma (plasma), bovine serum albumin (BSA), bovine fibrinogen (BF), and mouse blood. Corresponding ICG solutions were also prepared for comparison. The solutions were excited with a 785 nm laser and NIR‐II fluorescence signals were collected with a 1300 nm long‐pass filter. The results (Figure 4c) show large signal increases for ICG in blood, plasma, and BSA compared to PBS and saline solutions, consistent with previous reports. XW‐03‐66 signal in plasma and BSA does not show any significant increase compared to PBS and saline solutions. It shows a 2x increase in the blood (consistent with the pharmacokinetic data) and a 1.5x increase in fibrinogen, suggesting that fibrinogen is the major contributor to in vivo signal enhancement observed in the first 30 min of the pharmacokinetic studies.

### 2.6 In Vivo Tumor Imaging and Intraoperative Tumor Resection

Solid tumors are characterized by higher vascular density, leaky vessels, and impaired lymphatic clearance.^[^ ^71^ ^]^ In normal and inflamed tissue, macromolecules with molecular weight > 40 kDa and nanoparticles present in the interstitial fluid are cleared via the lymphatic system, while smaller molecules readily redistribute to the blood via diffusion and/or convection.^[^ ^72^ ^]^ In solid tumors, the leaky vasculature results in higher‐than‐normal extravasation of the solute content of serum into the tumor interstitial space and poor lymphatic clearance. This enhances the accumulation of macromolecules and nanoparticles in the tumor, a phenomenon known as the enhanced permeation and retention (EPR) effect.^[^ ^31^ ^]^ OSCCs make up about 90% of head and neck solid tumors and their resection is particularly challenging due to the complex anatomy of the oral cavity. As a result, the reported rate of successful resection outcomes with adequate margin delineation is currently only 50–75%. We hypothesized that i.v. administration of NIR‐II dyes such as XW‐03‐66 has the potential to accumulate in these tumors via the EPR effect, thus enabling NIR‐II image‐guided intraoperative resection with improved adequate margin delineation. To test this hypothesis, two different syngeneic murine models of head and neck squamous cell carcinoma were used: 1) the human papillomavirus (HPV)‐positive immunologically “hot” mEER tumor model, featuring murine pharyngeal epithelial cells transformed with HPV16 E6 and E7 oncogenes and H‐ras,^[^ ^73^ ^]^ and 2) HPV‐ve, the carcinogen‐induced, immunologically “cold” Mouse Oral Cancer 2 (MOC2) tumor model.^[^ ^74^, ^75^ ^]^

**Figure 5:**
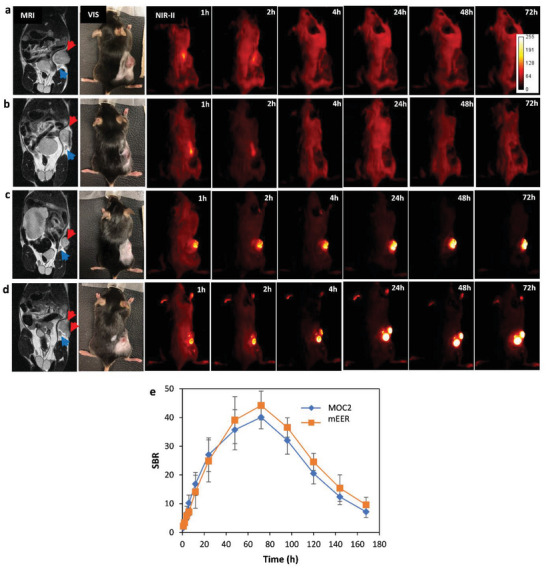
Preoperative MRI and in vivo NIR‐II tumor imaging. NIR‐II imaging with ICG: a) Preoperative MR image shows the location of the tumor (red arrowhead) and draining inguinal lymph node (blue arrowhead) in a MOC2 tumor‐bearing mouse. NIR‐II imaging after intravenous (i.v.) administration of ICG demonstrates a faint tumor signal at 1–2 h, and no apparent signal thereafter. b) Preoperative MR image of mEER tumor‐bearing mouse confirms the presence of the tumor. NIR‐II images at different time points following i.v. administration of ICG shows a faint tumor signal at 1–2 h, and no apparent signal thereafter. NIR‐II imaging with XW‐03‐66: NIR‐II images at different time points following i.v. administration of XW‐03‐66 in c) MOC2 and d) mEER tumor‐bearing mice demonstrate strong tumor signal which increases over time. e) A plot of tumor SBR versus time over a period of 168 h (7 days) shows a peak at 72 h (3 days), which also corresponds to the time at which signal in systemic circulation returns to baseline levels.

Test mice (*n* = 4 for each model) were first subjected to preoperative MRI scans to establish the presence of the tumor (a full panel of all preoperative images of all mice is shown in Figure S1.5, Supporting Information**.)** Following MRI confirmation, tumor‐bearing mice were administered XW‐03‐66 (dose = 5 mg kg^−1^) by tail vein injection. For comparison, a similar number of mice with MRI‐confirmed tumors were injected with clinically approved ICG at a similar dose. To monitor the dynamics of tumor uptake, NIR‐II images were collected at different time intervals over a period of seven days. As shown in **Figure** **5**a,b, mice injected with ICG showed no signal uptake in either tumor model. Signal was observed in the liver in images captured within the first 4 h but completely disappeared thereafter. NIR‐II images from mice injected with XW‐03‐66 started showing a signal in the tumor within 1‐h post‐injection, which increased in intensity over time. Both the MOC2 (Figure 5c) and mEER (Figure 5d) tumor models showed uniform uptake of the dye. A plot of SBR against time (Figure 5e) showed maximum tumor signal at 72‐h time point. This also corresponds to the time at which the dye is completely cleared from the systemic circulation, as determined in our pharmacokinetics experiments. Beyond this point, signal intensity in the tumor slowly decreased through day 7. For comparison, a panel of images showing the dynamics of tumor signal changes in all mice up to 72‐h time point, along with the preoperative MRI and white light images, is shown in Figures S1.6 and S1.7, Supporting information.

**Figure 6:**
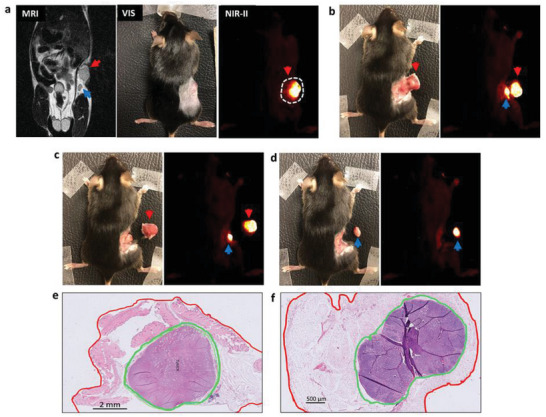
NIR‐II image‐guided resection of tumor (red arrowhead) and draining lymph node (blue arrowhead) with negative margins around the entire tumor mass and lymph node, respectively. a) Tumor images as seen with the three different imaging modalities employed in the operation; b) Dissection of the primary tumor (red arrowhead) reveals a bright draining lymph node (blue arrowhead) underneath as seen in the NIR‐II image; c) Complete removal of primary tumor; d) Complete removal of draining lymph node; e and f) H&E images confirm removal of entire tumor mass and lymph node (green line indicates lymph node boundary) with negative margins (red line).

Real‐time NIR‐II image‐guided tumor resection was performed at 72 h after i.v. administration of XW‐03‐66 when the tumor appeared brightest, with on‐screen image guidance. The mouse was euthanized by CO_2_ exposure prior to tumor resection, which proceeded in three major steps. First, the NIR‐II camera was turned on and a resection line was drawn at about 1–2 mm from the edge of the glowing tumor using onscreen guidance (represented by the white broken line in **Figure** **6**a). In the second step, the camera was turned off and dissection proceeded through the resection line with visible light. The visible light was turned off and the NIR‐II camera turned on again as needed throughout the process to ensure a 1–2 mm thick tissue layer was maintained between the glowing tumor and the resection margin. A second glowing spot (inguinal lymph node) was observed after cutting about halfway into the primary tumor (Figure 6b). Following the complete removal of the primary tumor (Figure 6c), the same sequence was repeated to remove the second glowing spot (Figure 6d). In the final step of the procedure, the excised tumors were sent for pathology analysis and the findings were compared with intraoperative observations. As shown in the H&E images in Figure 6e,f, histology data confirmed the tissue architecture in the tumor (Figure 6e) and a metastatic lymph node (Figure 6f). More remarkably, the pathology results showed that real‐time imaging allowed a consistent negative resection margin (red line) ranging between 400 µm to 2 mm around the entire specimen with no positive margins; the entire tumor boundary (green line) remained intact. Similarly, H&E‐stained sections from the lymph node specimen showed negative resection margins around the entire lymph node boundaries (green line). A panel of H&E‐stained sections from other tumors and lymph nodes is presented in the supporting information section (Figure S1.8, Supporting Information).

### 2.7 Preliminary Toxicity Profile of XW‐03‐66

**Figure 7:**
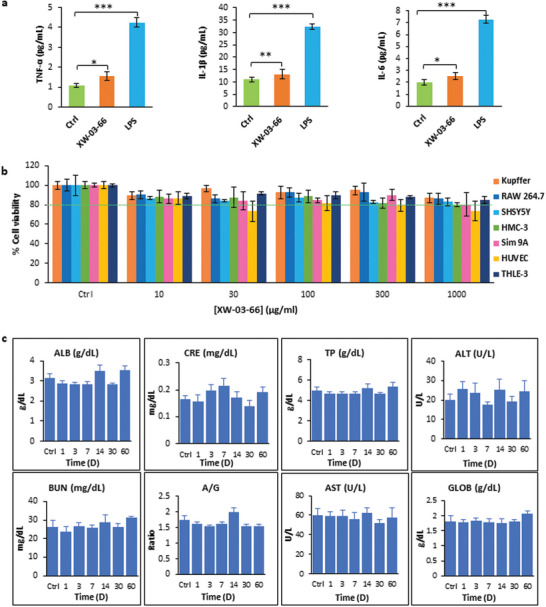
Preliminary toxicity evaluation indicated a good safety profile for XW‐03‐66. a) Kupffer cells exposed to XW‐03‐66 at a concentration of 1000 *µ*g mL^−1^ showed minimal change in the level of key inflammatory markers including TNF‐*α*, IL‐1*β*, and IL‐6, compared to cells exposed to LPS (10 ng), and untreated controls; b) Exposure of seven different cell lines to solutions of XW‐03‐66 at concentrations up to 1000 *µ*g mL^−1^ showed cell viability of over 80% or higher after 24 h; c) Serum biochemistry studies on C57BL/6 mice treated with XW‐03‐66 (dose 5 mg kg^−1^, *n* = 4), over a 60‐day period showed no changes in any of the toxicity indicators out of the normal range. *p <0.05, **p < 0.01, ***p < 0.001

To evaluate XW‐03‐66's effect on live cells, its inflammatory potential and cytotoxicity were tested in vitro. Three different immune cell lines, including Kupffer cells (liver resident immune cells), RAW 264.7 cells (a mouse macrophage cell line), and HMC‐3 cells (a human microglia cell line) were used to evaluate the inflammatory potential. Cells were incubated in a 1000 µg mL^−1^ solution of the agent in PBS for 24 h and three different inflammation markers (TNF‐*α*, IL‐1*β*, and IL‐6) were assessed. Non‐treated cells were used as a negative control and cells treated with LPS were used as a positive control. As exemplified by the Kupffer cells results (**Figure** **7**a), while there is a statistically significant increase in levels of all three markers in XW‐03‐66‐treated cells compared to untreated controls, both are 2–3x lower than the LPS‐treated samples. Data from RAW 264.7 and HMC‐3 cells, presented in Figure S1.9, Supporting Information section also show similar trends. To further evaluate its cytotoxicity, XW‐03‐66 was incubated at different injectable concentrations (up to 1000 µg mL^−1^) with seven different cell lines including Kupffer, RAW 264.7, HMC‐3, SH‐SY5Y, HUVEC, Sim 9A, and THLE‐3. After 24 h following incubation, all cell lines (except the HUVEC cells) showed, on average, 80% or more cell survival (Figure 7b), suggesting that the dye is only mildly cytotoxic at injectable concentrations.

In vivo toxicity of XW‐03‐66 was monitored over a 60‐day period. As demonstrated earlier (Figure 4h), there was no noteworthy difference in the body weight of animals injected with XW‐03‐66 compared to controls. Serum biochemistry results (Figure 7c) showed that albumin (ALB), globulin (GLOB), albumin/globulin (A/G), and total protein (TP) levels remained within normal ranges for C57BL/6 mice over the entire period.^[^ ^76^, ^77^ ^]^ Levels of alanine aminotransferase (ALT) and aspartate aminotransferase (AST), two liver enzymes that are key reporters of liver function, also remained within the normal range, indicating no signs of liver injury. Serum biochemistry results also showed that blood urea nitrogen (BUN) and creatinine (CRE), both reporters of kidney function, were within the normal range throughout the 60‐day period, indicating that XW‐03‐66 does not have any adverse effects on kidney function.

**Figure 8:**
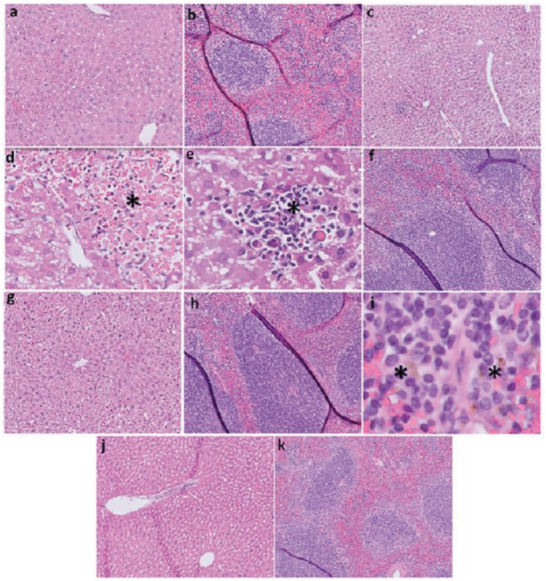
Histological analysis of liver and spleen over a 60‐day period following administration of XW‐06‐66 to C57BL/6J mice (*n* = 4). Controls: a) Hematoxylin and eosin (H&E) stained sections showing normal liver architecture. Hepatocytes show mild reactive changes with focal vacuolization of the cytoplasm. There is sinusoidal vascular congestion. The portal spaces are unremarkable (10x); b) Spleen shows no significant pathologic alteration (10x). 14 days post injection: c) Normal overall architecture. Hepatocytes show reactive changes with diffuse micro and macrosteatosis that seems to be of similar intensity in zone 1, 2, and 3. There is sinusoidal vascular congestion (10x); d) Apoptotic hepatocytes and necrosis is seen (*) (40x); e) Focal parenchymal chronic inflammation with associated apoptotic hepatocytes are seen (*) (40x). f) The spleen sections show follicular hyperplasia (10x). 28 days post‐injection: g) The liver shows normal overall architecture. Hepatocytes show reactive changes with diffuse micro and macrosteatosis that seems to be of similar intensity in zone 1, 2, and 3. There is sinusoidal vascular congestion. The portal spaces are unremarkable (10x). h) The spleen shows persistent follicular hyperplasia (10x); i) focal hemosiderin deposition is seen (*) (40x). 60 Days post‐injection: j) The liver shows normal overall architecture. Hepatocytes show minimal reactive changes with micro steatosis. There is sinusoidal vascular congestion. The portal spaces are unremarkable (10x). k) the spleen shows no significant pathologic alteration (10x).

Given the long residence time of XW‐03‐66 in the liver and spleen, we performed a histopathological analysis of H&E‐stained tissue sections at different time points over the 60‐day post‐injection period (**Figure** **8**). Analysis of H&E stained liver sections from sham controls at two weeks post‐injection showed normal overall architecture. Hepatocytes showed mild reactive changes with focal vacuolization of the cytoplasm and sinusoidal vascular congestion. The portal spaces were unremarkable. XW‐03‐66‐treated animals showed normal overall architecture and some reactive changes of the hepatocytes, consisting of diffuse vacuolization of the cytoplasm, (consistent with the sham‐treated controls), as well as macro‐ and microsteatosis. In addition, some focal parenchymal chronic inflammation with associated apoptotic hepatocytes was also observed. Treated sections at 1 month post‐injection also showed normal overall architecture, the presence of diffuse vacuolization of the cytoplasm, and macro‐ and microsteatosis, but no evidence of inflammation or apoptosis. Treated sections collected at 2 months post‐injection showed no evidence of pathologic alteration. Both sham and XW‐03‐66‐treated spleen sections showed normal overall architecture at 2 weeks and 1 month post‐injection, but sections from animals treated with the agent also showed some reactive follicular hyperplasia These changes were more pronounced 2 weeks after administration when hemosiderin‐laden macrophages were also present. Samples at 2 months post‐injection showed no significant pathologic alteration in both test and control animals. This data is consistent with the mild inflammatory potential and mild cytotoxicity observed in the in vitro assays, and the overall normal serum biochemistry results.

## 3 Conclusion 

In summary, we capitalized on the hydration properties of sucrose to develop a double‐shielded novel high‐performance **S‐**D‐A‐D‐S‐type NIR‐II molecule that self‐assembles into nanoscale mesoscopic solute‐rich clusters, enhancing both in vivo optical properties and imaging function. The classical approach to improving the performance of NIR‐II small organic molecule dyes is to add hydrophobic groups around the core fluorophore (also hydrophobic) to reduce solvent‐induced quenching and then append either PEG or ionic moieties such as carboxyl or sulphonate groups to render them water‐soluble. We hypothesized that the right choice of solubilizing moiety can provide additional shielding to the entire hydrophobic core, generating a shield‐shield‐donor‐acceptor‐donor‐shield‐shield (S‐S‐D‐A‐D‐S‐S) system, further enhancing the performance of the dye. Our data shows that the choice of eight sucrose units around the fluorophore in XW‐03‐66 does not result in a complete shield but provides a construct that dries molecular self‐assembly and nanoparticle formation. The enhanced fluorescence property of the dye appears to be due to the multiplet effect of the individual molecules within the particle rather than primarily from the effective shielding of the sucrose moieties, which apper to provide stability to the particles via an extended hydrogen bondibg network. However, this appears to be the case with CPK‐03‐37 in which the fluorophore is completely enveloped by the PEG units. The data demonstrate that the XW‐03‐66 molecule self‐assembles in aqueous media to form mesoscopic solute‐rich clusters with a hydrodynamic diameter of 80 ± 5 nm and a QY of 6.0%. The amount of solute captured in the clusters and the related number of clusters and cluster population volume increase exponentially with the solute concentration but overall fluorescence intensity increases linearly, suggesting that aggregation does not influence fluorescence performance. This corroborates with the observation that nanoparticles are mesoscopic solute‐rich clusters and not highly organized crystal structures, which would otherwise result in some aberration in fluorescence properties with increasing particle concentration. The ability of XW‐03‐66 to self‐assemble into stable nanoparticles raises its in vivo imaging functionality to that of organic polymeric nanofluorophores and inorganic nanomaterials, while maintaining the safety profile of a small molecule. The observed long in vivo circulation half‐life enables the acquisition of high‐resolution vascular images for over 6 h and passive accumulation of the probe in tumors via the EPR effect. Such results are only possible with nanoparticle‐based probes. The brightness and photostability of the dye allow for real‐time high‐resolution NIR‐II tumor imaging, enabling the resection of 5 mm tumors and draining lymph nodes with all‐round negative margins. More importantly, preliminary toxicity data suggest that the dye is well‐tolerated in rodents.

## Conflict of Interest

K.B.G., A.V.A., and E.A.T. own stock and/or serve as consultants at Alzeca Biosciences, Inc.

## Supporting information

Supporting Information 1

Supporting Information 2

## Data Availability

The data that support the findings of this study are available in the supplementary material of this article.
