# Supplementary material for: Rational Design of a Self‐Assembling High Performance Organic Nanofluorophore for Intraoperative NIR‐II Image‐Guided Tumor Resection of Oral Cancer
Source: Adv Sci (Weinh). 2023 Jan 31;10(10):2206435. doi: 10.1002/advs.202206435 (PMC10074073; doi:10.1002/advs.202206435)
Supplement: Supplementary file 1 — Supporting Information 1 [file ADVS-10-2206435-s002.pdf]

ADVANCED SCIENCE
Open Access

# Supporting Information

for *Adv. Sci.*, DOI 10.1002/advs.202206435

Rational Design of a Self-Assembling High Performance Organic Nanofluorophore for Intraoperative NIR-II Image-Guided Tumor Resection of Oral Cancer

*Xianwei Sun, Praveen Kumar Chintakunta, Andrew A. Badachhape, Rohan Bhavane, Huan-Jui Lee, David S. Yang, Zbigniew Starosolski, Ketan B. Ghaghada, Peter G. Vekilov, Ananth V. Annapragada and Eric A. Tanifum**

**Supporting information 1 (S1)**

# Rational Design of a self-assembling high performance organic nanofluorophore for intraoperative NIR-II image-guided tumor resection in mouse models of oral cancer

*Xianwei Sun[1], Praveen Kumar Chintakunta[1,2], Andrew Badachhape[1,3], Rohan Bhavane[1,3], Huan-Jui Lee[4], David S. Yang[4], Zbigniew Starosolski[1,3], Ketan Ghaghada[1,3], Peter Vekilov[4,5], Ananth Annapragada[1,3], and Eric Tanifum[1,3]**

[1]Department of Radiology, Baylor College of Medicine, Houston, TX 77030, USA.

[2] Current Address: Sai Life Sciences Ltd, Turakapally, Telangana, India.

[3]Department of Radiology, Texas Children's Hospital, Houston, TX 77030, USA.
[4]Department of Chemical and Biomolecular Engineering, University of Houston, Houston, TX 77204, USA.
[5]Department of Chemistry, University of Houston, Houston, TX 77204, USA

*E-mail: eatanifu@texaschildrens.org

**TABLE OF CONTENTS**

1. General information and experimental methods……………………………………….......S1.3
2. Synthetic procedures and characterization data for target compounds and intermediates…S1.5
3. Supplementary figures…………...……………………………………………………….S1.18
4. References……………………………………………………………….…………….S1.28

## 1. General information and methods

**Materials**. Unless otherwise noted, all reagents and solvents were obtained from commercial sources including, Sigma-Aldrich, TCI, Alfa Aesar, and Acros Organics and used without further purification. **IR-26** was purchased from BOCSCI INC, New York, USA. Thin layer chromatography (TLC) was performed on silica gel 60 F254 plates from EMD Chemical Inc. and components were visualized by ultraviolet light (254 nm) and/or phosphomolybdic acid, 20 wt% solution in ethanol. SiliFlash silica gel (230–400 mesh) was used for all column chromatography.
**Measurements**. Proton nuclear magnetic resonances ($^1$H NMR) were recorded at 600 MHz or 500 MHz on Bruker 600 or 500 NMR spectrometers. Carbon nuclear magnetic resonances ($^{13}$C NMR) were recorded at 75 MHz or 125 MHz on a Bruker 300 or 500 NMR spectrometers respectively. Chemical shifts are reported in parts per million (ppm) from an internal standard acetone (2.05 ppm), chloroform (7.26 ppm), or dimethylsulfoxide (2.50 ppm) for $^1$H NMR; and from an internal standard of either residual acetone (206.26 ppm), chloroform (77.00 ppm), or dimethylsulfoxide (39.52 ppm) for $^{13}$C NMR. NMR peak multiplicities are denoted as follows: s (singlet), d (doublet), t (triplet), q (quartet), dd (doublet of doublet), td (doublet of triplet), dt (triplet of doublet), and m (multiplet). Coupling constants ($J$) are given in hertz (Hz). High resolution mass spectra (HRMS) were obtained from The Ohio State University Mass Spectrometry and Proteomics Facility. Ultraviolet-visible-near infrared (UV-VIS-NIR) absorption spectra were recorded on a UV-1600PC spectrophotometer and NIR-II fluorescence spectra were acquired using a NS3 NanoSpectralyzer (Applied NanoFluorescence, LLC., Houston, TX, USA). Cluster size was determined by Oblique Illumination Microscopy (OIM) and Atomic Force Microscopy (AFM) data was collected on a Bruker MultiMode 8 microscope and analyzed using NanoScope Analysis 1.5 software (Bruker). NIR-II fluorescence imaging was conducted on a home-built instrument (all key components from ThorLabs, Newton, NJ, USA) including a 785 nm laser for excitation and a Raptor-Ninox 640 SWIR camera (Raptor Photonics, Ltd., Northern Ireland) equipped with a 1300 nm long pass filter (ThorLabs Inc.). Images were processed and analyzed using ImageJ software.
**Synthesis method I**: $K_2CO_3$ (2.0 eq) and KI (0.1 eq) were slowly added to a solution of amine (1.0 eq) and bromide reagent (1.5 eq) in dry DMF (10 mL). The reaction mixture was stirred at 90 $^0$C overnight, and then cooled to room temperature. The cooled reaction mixture was filtered and evaporated. The ensuing residue was purified by column chromatography to obtain the desired compound.

**Synthesis method II (Suzuki coupling)**: A solution of the desired bromide derivative (2.0 eq), boronic acid derivative (1.0 eq), $K_2CO_3$ (3.0 eq) in Toluene/ $H_2O$ (4:1, 10 mL) was deoxygenated by bubbling argon through for 20 minutes. To this was added $Pd(PPh_3)_4$ (0.1 eq) and argon bubbled through for a further 5 minutes, then stirred at 110 °C overnight. The reaction mixture was then cooled and diluted with water (5 mL), and the aqueous layer extracted with ethyl acetate. The combined organic layer was then washed with saturated $NaHCO_3$, rinsed with brine, dried over $Na_2SO_4$, and concentrated under reduced pressure. The ensuing residue was purified by column chromatography to obtain the desired compound.

**Synthesis method III (Stille coupling)**: A solution of thiophenyl derivative (1.0 eq) in THF under positive inert gas atmosphere was cooled down to -78 °C, *n*-BuLi (2.5 M in hexane, 1.5 eq) was slowly added. The mixture was stirred at this temperature for 2 h, and then tributyltinchloride (2.0 eq) was slowly added. After 10 minutes, the reaction was slowly warmed up to room temperature and stirred overnight. The mixture was poured into ice water and extracted with ethyl acetate, and the combined organic phase washed with saturated $NaHCO_3$, rinsed with brine, dried over $Na_2SO_4$, evaporated and dried by high *vacuo* without any further purification. A solution of the crude product (3.0 eq) and bromide derivative (1.0 eq) in dry toluene (10 mL) was deoxygenated by bubbling argon through for 20 minutes. To this was added $Pd(PPh_3)_2Cl_2$ (0.1 eq) and argon bubbled through for a further 5 minutes, then stirred at 110 °C for 24 hours. The reaction mixture was then cooled and diluted with water (5 mL), and the aqueous layer extracted with ethyl acetate. The combined organic layer was then washed with saturated $NaHCO_3$, rinsed with brine, dried over $Na_2SO_4$ and concentrated under reduced pressure. The ensuing residue was purified by column chromatography to obtain the desired compound.

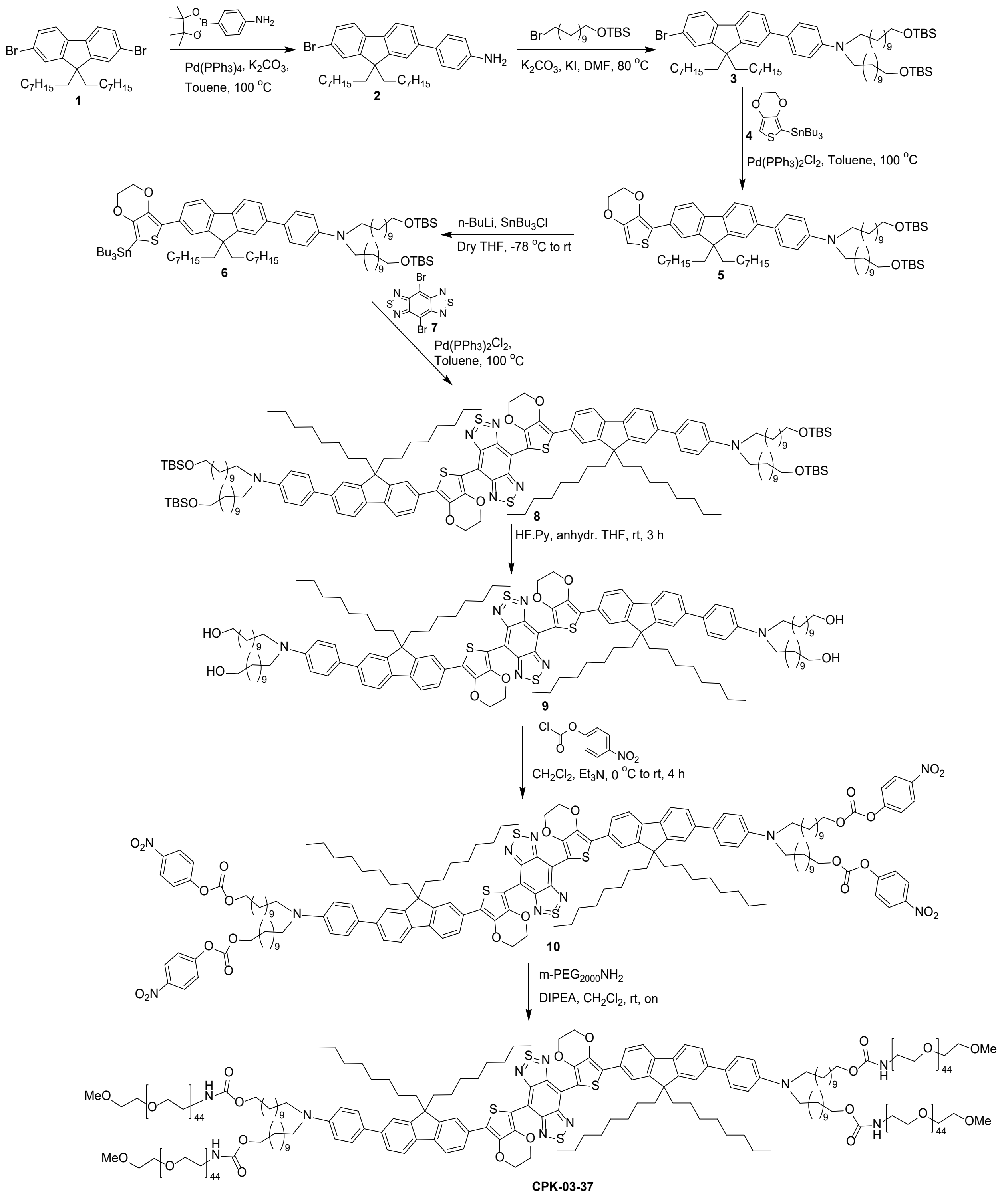


**Scheme S1.1.** Synthetic route to **CPK-03-37**.

**Compound 2:** Prepared by **Method II** with 9,9-dioctyl-2,7-dibromofluorene (25 g, 45.58 mmol), $Pd(PPh_3)_4$ (1.58 g, 1.37 mmol), $K_2CO_3$ (5.71 g, 41.1 mmol) and 4-(diethylamino)phenylboronic acid (3.0 g, 13.7 mmol) to afford compound **2** as a colourless oil (5.0 g, 65% yield). $^1H$ NMR (600 MHz, $CDCl_3$) δ 7.69 (d, *J* = 7.8 Hz, 1H), 7.56 (d, *J* = 7.8 Hz, 1H), 7.55-7.47 (m, 5H), 6.83 (d, *J* = 8.4 Hz, 2H), 3.79 (brs, 2H), 2.03-1.96 (m, 4H), 1.25-1.21 (m, 4H), 1.17-1.08 (m, 16H), 0.84 (t, *J* = 7.2 Hz, 6H), 0.70-0.67 (m, 4H); $^{13}C$ NMR (150 MHz, $CDCl_3$) δ 153.1, 150.9, 145.5, 140.5, 139.9, 138.3, 131.9, 129.9, 128.0, 126.1, 125.3, 120.8, 120.7, 120.6, 119.9, 115.5, 55.4, 40.3, 31.7, 29.9, 29.2, 29.1, 23.7, 22.6, 14.0. HRMS(ESI) calcd for $C_{35}H_{46}BrN$ ([M+Na$^+$]): 583.6235, found 583.6311.

**Compound 3:** Prepared by **Method I** with compound **2** (5.0 g, 8.92 mmol), $K_2CO_3$ (4.96 g, 35.7 mmol), KI (299 mg, 1.8 mmol) and [(11-bromoundecyl)oxy)(tert-butyl)]dimethylsilane (9.45 g, 26.8 mmol) to afford compound **3** as a colourless oil (2.0 g, 20% yield). $^1H$ NMR (600 MHz, $CDCl_3$) δ 7.67 (d, *J* = 7.8 Hz, 1H), 7 55-7.50 (m, 4H), 7.48 (s, 1H), 7 45-7.43 (m, 2H), 6.74 (d, *J* = 9.0 Hz, 2H), 3.61 (t, *J* = 6.6 Hz, 4H), 3.32 (t, *J* = 7.8 Hz, 4H), 2.00-1.92 (m, 4H), 1.63 (brs, 4H), 1.54-1.51 (m, 6H), 1.35-1.30 (m, 30H), 1.22-1.19 (m, 6H), 1.15-1.06 (m, 18H), 0.91 (s, 18H), 0.83 (t, *J* = 7.2 Hz, 6H), 0.66 (m, 4H), 0.06 (s, 12H); $^{13}C$ NMR (150 MHz, $CDCl_3$) δ 150.8, 147.5, 140.7, 140.2, 137.8, 129.8, 127.9, 127.8, 126.0, 124.9, 120.7, 120.4, 119.9, 111.8, 63.3, 55.1, 51.2, 40.4, 32.9, 31.8, 29.9, 29.6,7, 29.6, 29.58, 29.4, 29.2, 29.1, 27.3, 27.2, 25.9, 25.8, 23.7, 22.6, 18.4, 14.1, -5.2. HRMS(ESI) calcd for $C_{69}H_{118}BrNO_2Si_2$ ([M+H$^+$]): 1130.7895, found 1130.7921.

**Compound 5:** Prepared by **Method III** with compound **3** (2.0 g, 1.81 mmol), $Pd(PPh_3)_2Cl_2$ (126 mg, 0.18 mmol), and tributyl(2,3-dihydrothieno[3,4-b][1,4]dioxin-5-yl)stannane **4** ( 1.17 g, 2.72 mmol) to afford compound **5** as a yellow oil (1.05 mg, 50% yield). $^1H$ NMR (600 MHz, $CDCl_3$) δ 7.73 (dd, $J_1$ = 1.8 Hz, $J_2$ = 7.8 Hz, 1H), 7.68 (d, *J* = 7.8 Hz, 1H), 7.66 (d, *J* = 7.8 Hz, 1H), 7.63 (d, *J* = 1.2 Hz, 1H), 7.55 (d, *J* = 9.0 Hz, 2H), 7.52 (dd, $J_1$ = 1.2 Hz, $J_2$ = 7.8 Hz, 1H), 7.50 (s, 1H), 6.73 (d, *J* = 9.0 Hz, 2H), 6.31 (s, 1H), 4.37-4.36 (m, 2H), 4.29-4.28 (m, 2H), 3.61 (t, *J* = 6.6 Hz, 4H), 3.31 (t, *J* = 7.8 Hz, 4H), 2.00-1.98 (m, 4H), 1.66-1.62 (m, 8H), 1.53-1.50 (m, 4H), 1.39-1.30 (m, 46H), 1.20-1.19 (m, 6H), 1.11-1.06 (m, 20H), 0.95-0.90 (m, 30H), 0.81 (t, *J* = 7.2 Hz, 6H), 0.72-0.71 (m, 4H), 0.06 (s, 12H); $^{13}C$ NMR (150 MHz, $CDCl_3$) δ 151.5, 151.3, 148.8, 147.4, 142.3, 140.1, 139.8, 138.6, 137.8, 131.4, 128.2, 127.8, 124.8, 124.79, 119.7, 119.5, 118.5, 111.8, 97.0, 64.8, 64.5, 63.3, 55.1, 51.2, 40.4, 32.9, 31.8, 30.1, 29.68, 29.61, 29.59, 29.56, 29.4,

29.2, 29.19, 27.3, 27.2, 25.9, 25.8, 23.8, 22.6, 18.4, 14.1, -5.2. HRMS(ESI) calcd for $C_{75}H_{123}NO_4SSi_2$ ([M+Na$^+$]): 1214.0312, found 1214.0389.

**Compound 6**: A solution of compound **5** (1.0 g, 0.86 mmol) in THF under positive inert gas atmosphere was cooled down to -78 °C, *n*-BuLi (2.5 M in hexane, 0.52 mL, 1.28 mmol) was slowly added. The mixture was stirred at this temperature for 2 hours, and then tributyltinchloride (559 mg, 1.72 mmol) was solwly added. After 10 minutes, the reaction was slowly warmed up to room temperature and stirred overnight. The mixture was poured into ice water and extract with ethyl acetate. The organic phase was washed with saturated $NaHCO_3$, rinsed with brine, dried over $Na_2SO_4$, evaporated and dried by high *vacuo* without any further purification.

**Compound 8:** Prepared by **Method III** with compound **6** (1.1 g, 0.76 mmol), $Pd(PPh_3)_2Cl_2$ ( 28 mg, 0.04 mmol), and compound **A** ( 60 mg, 0.17 mmol) to afford compound **8** as a dark solid (150 mg, 35% yield). $^1$H NMR (600 MHz, $CDCl_3$) δ 7.91 (dd, $J_1$ = 1.8 Hz, $J_2$ = 7.8 Hz, 2H), 7.77 (s, 2H), 7.48 (dd, $J_1$ = 3.2 Hz, $J_2$ = 7.8 Hz, 4H), 7.59-7.54 (m, 6H), 7.52 (s, 2H), 6.74 (d, *J* = 8.4 Hz, 4H), 4.54 (brs, 4H), 4.41 (brs, 4H), 3.62 (t, *J* = 6.6 Hz, 8H), 3.32 (t, *J* = 7.2 Hz, 8H), 2.04-2.03 (m, 8H), 1.63 (m, 8H), 1.54-1.51 (m, 8H), 1.35-1.30 (m, 60H), 1.21-1.19 (m, 12H), 1.15-1.06 (m, 36H), 0.91 (s, 36H), 0.82 (t, *J* = 7.2 Hz, 12H), 0.73 (brs, 8H), 0.07 (s, 24H); $^{13}$C NMR (150 MHz, $CDCl_3$) δ 152.6, 151.6, 151.3, 147.5, 141.9, 140.5, 140.3, 138.6, 138.2, 131.1, 128.2, 127.8, 125.5, 124.8, 123.1, 120.8, 120.4, 119.8, 119.5, 113.1, 111.9, 108.6, 64.6, 64.5, 63.3, 55.2, 51.2, 40.5, 32.9, 31.8, 30.1, 29.7, 29.6, 29.56, 29.56, 29.4, 29.3, 29.2, 27.3, 27.2, 25.9, 25.8, 23.8, 22.6, 18.4, 14.1, -5.2. HRMS(ESI) calcd for $C_{156}H_{244}N_6O_8S_4Si_4$ ([M+Na$^+$]): 2595.2712, found 2595.2798.

**Compound 9**: A solution of compound **8** (150 mg, 0.06 mmol) in THF under positive inert gas atmosphere was cooled down to 0 °C, and HF/Pyr (sigma-aldrich 70% HF-30% Pyridine; 1 mL) was slowly added. The mixture was stirred at room temperature overnight. The mixture was poured into ice water and extracted with ethyl acetate. The combined organic extract was then washed with saturated $NaHCO_3$, rinsed with brine, dried over $Na_2SO_4$ and concentrated under reduced pressure. The ensuing residue was purified by column chromatography to obtain the desired compound **9** as a dark solid (75 mg, 62% yield). $^1$H NMR (600 MHz, $CDCl_3$) δ 7.91 (d, *J* = 9.0 Hz, 2H), 7.76 (s, 2H), 7.48 (d, *J* = 9.0 Hz, 4H), 7.58-7.52 (m, 8H), 6.74 (d, *J* = 8.4 Hz, 4H), 4.53 (m, 4H), 4.40 (m, 4H), 3.62 (t, *J* = 6.6 Hz, 8H), 3.32 (t, *J* = 7.2 Hz, 8H), 2.04-2.02 (m, 8H), 1.63-1.56 (m, 16H), 1.35-1.18 (m, 72H), 1.13-1.08 (m, 36H), 0.81 (t, *J* = 7.2 Hz, 12H), 0.73 (m, 8H);

$^{13}C$ NMR (150 MHz, $CDCl_3$) δ 152.6, 151.6, 151.3, 147.5, 141.9, 140.4, 140.3, 138.6, 138.2, 131.1, 128.2, 127.8, 125.5, 124.8, 123.1, 120.8, 120.4, 119.8, 119.5, 113.1, 111.9, 108.6, 64.7, 64.6, 63.1, 55.2, 51.2, 40.5, 32.8, 31.8, 29.6, 29.5, 29.4, 29.2, 27.3, 27.2, 25.7, 23.8, 22.6, 14.1. HRMS(ESI) calcd for $C_{132}H_{188}N_6O_8S_4$ ([M+H$^+$]): 2114.2375, found 2114.2421.

**Compound 10**: A solution of compound **9** (40 mg, 0.02 mmol), triethylamine (20 mg, 0.20 mmol) and DMAP (3 mg) in DCM (6 mL) under positive inert gas atmosphere was cooled down to 0 °C, 4-nitrophenyl chloroformate (32 mg, 0.16 mmol) was added in one portion. After 10 minutes, the reaction was slowly warmed up to room temperature and stirred overnight. The mixture was poured into ice water and extracted twice with ethyl acetate, and then combined with organic phase. The organic phase was washed with saturated $NaHCO_3$, rinsed with brine, dried over $Na_2SO_4$. The ensuing residue was purified by column chromatography to obtain the desired compound **10** as a dark solid (50 mg, 94% yield). $^1H$ NMR (600 MHz, $CDCl_3$) δ 8.28 (dd, $J_1$ = 1.8 Hz, $J_2$ = 7.2 Hz, 8H), 7.91 (d, $J$ = 8.4 Hz, 2H), 7.76 (s, 2H), 7.72 (d, $J$ = 7.8 Hz, 4H), 7.57 (d, $J$ = 8.4 Hz, 4H), 7.54 (d, $J$ = 7.8 Hz, 2H), 7.52 (s, 2H), 7.39 (dd, $J_1$ = 1.8 Hz, $J_2$ = 7.2 Hz, 8H), 6.75 (d, $J$ = 8.4 Hz, 4H), 4.53 (m, 4H), 4.40 (brs, 4H), 4.30 (t, $J$ = 7.2 Hz, 8H), 3.33 (t, $J$ = 7.2 Hz, 8H), 2.04-2.02 (m, 8H), 1.79-1.76 (m, 8H), 1.63 (m, 8H), 1.45-1.41 (m, 8H), 1.36-1.28 (m, 48H), 1.22-1.18 (m, 8H), 1.15-1.08 (m, 32H), 0.80 (t, $J$ = 7.2 Hz, 12H), 0.73 (m, 8H); $^{13}C$ NMR (150 MHz, $CDCl_3$) δ 155.6, 152.6, 152.58, 151.6, 151.3, 147.4, 145.3, 141.9, 138.7, 138.2, 131.2, 127.8, 125.5, 125.3, 124.8, 121.8, 120.8, 120.4, 119.9, 119.6, 113.1, 111.9, 69.6, 64.7, 64.6, 55.2, 51.1, 40.5, 31.8, 29.6, 29.58, 29.5, 29.45, 29.2, 29.17, 28.5, 27.3, 27.2, 25.7, 25.6, 23.8, 22.6, 14.1. HRMS(ESI) calcd for $C_{160}H_{200}N_{10}O_{24}S_4$ ([M+Na$^+$]): 2798.6352, found 2798.6402.

**CPK-03-37:** A solution of compound **10** (50 mg, 0.018 mmol) and triethylamine (42 mg, 0.42 mmol) in DCM (6 mL) at 0 °C, mPEG-2000 amine (360 mg, 0.18 mmol) was added in one portion. After 10 minutes, the reaction was slowly warmed up to room temperature and stirred overnight. The organic solvents were evaporated, and the resulting residue diluted with methanol/water mixture (1:1, 8 mL). This was then loaded into a 10000 MWCO dialysis bag and dialyzed against MES buffer (50 mM, 2×5 L) 4 h and then water (2×5 L) overnight. The water was then removed by freeze drying to obtain the desired compound **CPK-03-37** as a dark powder (190 mg). $^1H$ NMR (600 MHz, $CDCl_3$) δ 7.92 (d, $J$ = 7.8 Hz, 2H), 7.82-7.76 (m, 18H), 7.58-7.53 (m, 2H), 7.16 (d, $J$ = 7.8 Hz, 1H), 5.23 (m, 1H), 4.53 (m, 1H), 4.40 (m, 1H), 4.01 (t, $J$ = 6.6 Hz, 6H), 3.91-3.89 (m, 6H), 3.76-3.74 (m, 34H), 3.71-3.61 (m, 4012H), 3.56-3.51 (m, 118H), 3.37-3.35

(m, 92H), 3.20-3.18 (m, 6H), 2.31 (m, 90H), 2.08-2.04 (m, 12H), 1.59-1.56 (m, 10H), 1.25-1.17 (m, 116H), 1.12-1.07 (m, 52H), 0.78 (t, $J$ = 6.6 Hz, 12H), 0.75-0.67 (m, 8H).

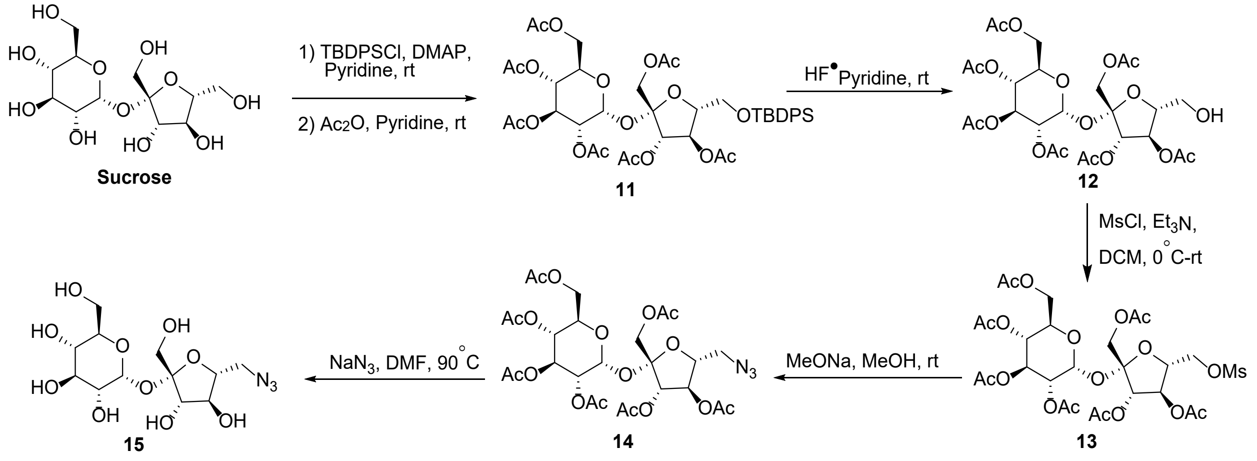


**Scheme S1.2.** Synthetic route to azido sucrose.

**Compound 11**: To a solution of sucrose (15 g, 43.9 mmol) and DMAP (0.1 g, 0.9 mmol) in dry pyridine (200 mL) was slowly dropped TBDPSCl (2.4 g, 8.8 mmol). The resulting mixture was stirred at room temperature for 12 h, followed by the addition of acetic anhydride (100 mL). The reaction mixture was stirred at room temperature overnight, followed by evaporation and the crude product diluted with ethyl acetate (200 mL) and then washed with 1 M HCl, saturated $NaHCO_3$, rinsed with brine, dried over $Na_2SO_4$, and concentrated under reduced pressure. The ensuing residue was purified by column chromatography to obtain the desired compound **11** as a yellow oil (6.1 g, 80% yield). $^1$H NMR (600 MHz, $CDCl_3$) δ 8.28 (dd, $J_1$ = 7.8 Hz, $J_2$ = 16.8 Hz, 4H), 7.37-7.30 (m, 6H), 5.57 (d, $J$ = 3.6 Hz, 1H), 5.45 (t, $J$ = 6.0 Hz, 1H), 5.34-5.31 (m, 2H), 4.96 (t, $J$ = 10.2 Hz, 1H), 4.75 (dd, $J_1$ = 3.6 Hz, $J_2$ = 10.2 Hz, 1H), 4.15-4.06 (m, 4H), 4.02 (q, $J$ = 6.0 Hz, 1H), 3.87 (dd, $J_1$ = 1.8 Hz, $J_2$ = 12.6 Hz, 1H), 3.77 (m, 2H), 2.06 (s, 3H), 2.03 (s, 3H), 2.02 (s, 3H), 1.97 (s, 3H), 1.94 (s, 3H), 1.91 (s, 3H), 1.89 (s, 3H), 0.99 (s, 9H); $^{13}$C NMR (150 MHz, $CDCl_3$) δ 170.6, 170.2, 170.1, 170.0, 169.9, 169.5, 135.6, 135.5, 132.9, 132.8, 129.9, 127.8, 127.7, 127.6, 103.7, 89.7, 81.4, 76.2, 75.1, 70.2, 69.8, 68.3, 68.1, 63.8, 62.9, 61.6, 26.7, 20.8, 20.7, 20.6, 20.5, 20.56, 19.2. HRMS(ESI) calcd for $C_{42}H_{54}O_{18}Si$ ([M+Na$^+$]): 897.2977, found 897.3002.

**Compound 12**: A solution of compound **11** (6.0 g, 6.8 mmol) in THF under positive inert gas atmosphere was cooled down to 0 °C and HF·Pyr (70% HF-30% Pyridine; 2 mL) was slowly added.

The mixture was stirred at room temperature overnight and then poured into ice water and extract trice with ethyl acetate. The combined organic layer was washed with saturated $NaHCO_3$, rinsed with brine, dried over $Na_2SO_4$ and concentrated under reduced pressure. The ensuing residue was purified by column chromatography to obtain the desired compound **12** as a yellow oil (4.0 g, 92% yield). $^1$H NMR (600 MHz, $CDCl_3$) δ 5.65 (d, $J$ = 3.6 Hz, 1H), 5.46-5.43 (m, 2H), 5.40 (t, $J$ = 10.2 Hz, 1H), 5.06 (t, $J$ = 10.2 Hz, 1H), 4.85 (dd, $J_1$ = 3.6 Hz, $J_2$ = 10.2 Hz, 1H), 4.26-4.23 (m, 2H), 4.15-4.12 (m, 2H), 4.07-4.02 (m, 2H), 4.02 (q, $J$ = 6.0 Hz, 1H), 3.82 (dd, $J_1$ = 3.0 Hz, $J_2$ = 12.6 Hz, 1H), 3.64 (dd, $J_1$ = 4.2 Hz, $J_2$ = 12.6 Hz, 1H), 2.16 (s, 3H), 2.09 (s, 3H), 2.08 (s, 3H), 2.076 (s, 3H), 2.07 (s, 3H), 2.02 (s, 3H), 1.99 (s, 3H); $^{13}$C NMR (150 MHz, $CDCl_3$) δ 170.6, 170.5, 170.0, 169.9, 169.8, 169.4, 103.2, 89.9, 81.6, 76.0, 73.6, 70.2, 69.3, 68.6, 67.9, 63.8, 61.3, 61.1, 20.7, 20.6, 20.56, 20.5, 20.49, 20.47. HRMS(ESI) calcd for $C_{26}H_{36}O_{18}$ ([M+Na$^+$]): 659.5568, found 659.5586.

**Compound 13**: To a solution of compound **12** (3.9 g, 6.1 mmol), triethylamine (1.2 g, 12.2 mmol) and DMAP (73 mg, 0.6 mmol) in DCM (60 mL) under positive inert gas atmosphere at 0 °C was slowly added Methanesulfonyl chloride (1.0 g, 9.2 mmol). After 10 minutes, the reaction was slowly warmed up to room temperature and stirred overnight. The mixture was poured into ice water and extracted with DCM. The combined organic phase was washed with saturated $NaHCO_3$, rinsed with brine, dried over $Na_2SO_4$, and concentrated. The ensuing residue was purified by column chromatography to obtain the desired compound **13** as a yellow oil (3.7 g, 85% yield). $^1$H NMR (600 MHz, $CDCl_3$) δ 5.62 (d, $J$ = 3.6 Hz, 1H), 5.48-5.45 (m, 2H), 5.29 (t, $J$ = 6.0 Hz, 1H), 5.06 (t, $J$ = 10.2 Hz, 1H), 4.88 (dd, $J_1$ = 3.6 Hz, $J_2$ = 10.2 Hz, 1H), 4.29-4.27 (m, 1H), 4.23-4.19 (m, 2H), 4.16-4.12 (m, 3H), 3.70 (dd, $J_1$ = 7.8 Hz, $J_2$ = 13.2 Hz, 1H), 3.49 (dd, $J_1$ = 3.6 Hz, $J_2$ = 13.2 Hz, 1H), 2.16 (s, 3H), 2.10 (s, 9H), 2.09 (s, 3H), 2.04 (s, 3H), 2.01 (s, 3H); $^{13}$C NMR (150 MHz, $CDCl_3$) δ 170.6, 170.1, 170.0, 169.9, 169.6, 169.5, 103.9, 90.3, 80.1, 75.7, 70.2, 69.4, 68.5, 68.2, 62.7, 61.9, 52.8, 20.7, 20.6, 20.59, 20.55, 25.3, 20.4. HRMS(ESI) calcd for $C_{27}H_{38}O_{20}S$ ([M+Na$^+$]): 737.1569, found 737.1567.

**Compound 14**: To a solution of **13** (3.5 g, 4.9 mmol) in dry DMF (60 mL) was slowly added $NaN_3$ (0.6 g, 9.8 mmol). The reaction mixture was stirred at 90 °C overnight, and then cooled to room temperature. The mixture was poured into ice water and extract trice with DCM, and the combined

organic extract washed with saturated $NaHCO_3$, rinsed with brine, dried over $Na_2SO_4$, and concentrated. The ensuing residue was purified by column chromatography to obtain the desired compound **14** as a yellow oil (2.6 g, 80% yield). $^1$H NMR (600 MHz, $CDCl_3$) δ 5.65 (d, $J$ = 3.6 Hz, 1H), 5.46-5.43 (m, 2H), 5.40 (t, $J$ = 10.2 Hz, 1H), 5.06 (t, $J$ = 10.2 Hz, 1H), 4.85 (dd, $J_1$ = 3.6 Hz, $J_2$ = 10.2 Hz, 1H), 4.26-4.23 (m, 2H), 4.15-4.12 (m, 2H), 4.07-4.02 (m, 2H), 4.02 (q, $J$ = 6.0 Hz, 1H), 3.81 (dd, $J_1$ = 3.0 Hz, $J_2$ = 12.6 Hz, 1H), 3.64 (dd, $J_1$ = 4.2 Hz, $J_2$ = 12.6 Hz, 1H), 2.16 (s, 3H), 2.09 (s, 3H), 2.08 (s, 3H), 2.076 (s, 3H), 2.07 (s, 3H), 2.02 (s, 3H), 1.99 (s, 3H); $^{13}$C NMR (150 MHz, $CDCl_3$) δ 170.6, 170.5, 170.0, 169.9, 169.8, 169.4, 103.2, 89.9, 81.6, 76.0, 73.6, 70.2, 69.3, 68.6, 67.9, 63.8, 61.3, 61.1, 20.7, 20.6, 20.56, 20.5, 20.49, 20.47. HRMS(ESI) calcd for $C_{26}H_{35}NO_{17}$ ([M+Na$^+$]): 684.1859, found 684.1857.

**Compound 15**: To a solution of **14** (2.5 g, 3.8 mmol) in dry methanol (50 mL) under positive argon pressure was added MeONa (0.1 g, 1.9 mmol). The reaction mixture was stirred at room temperature overnight and then neutralized by passing through a short bed of Amberlite IR-120 (H+-form) and washed with dry MeOH. The ensuing organic mixture was concentrated under reduced pressure to give the crude product which was purified by column chromatography to obtain the desired azido sucrose, compound **15**, as a white solid (1.1 g, 82% yield). $^1$H NMR (600 MHz, $CDCl_3$) δ 5.93 (d, $J$ = 3.0 Hz, 1H), 4.70 (d, $J$ = 8.4 Hz, 1H), 4.67-4.60 (m, 2H), 4.52-4.46 (m, 2H), 4.30 (t, $J$ = 9.6 Hz, 1H), 4.24 (dd, $J_1$ = 6.0 Hz, $J_2$ = 11.4 Hz, 1H), 4.22 (s, 2H), 4.03 (dd, $J_1$ = 2.4 Hz, $J_2$ = 7.2 Hz, 1H), 4.00 (dd, $J_1$ = 3.6 Hz, $J_2$ = 9.6 Hz, 1H), 3.98 (s, 1H), 3.83 (t, $J$ = 9.6 Hz, 1H); $^{13}$C NMR (150 MHz, $CDCl_3$) δ 105.5, 93.1, 81.9, 78.3, 77.2, 74.3, 74.1, 72.9, 71.7, 63.1, 62.5, 54.5, 49.9. HRMS(ESI) calcd for $C_{12}H_{21}NO_{10}$ ([M+Na$^+$]): 390.1119, found 390.1119.

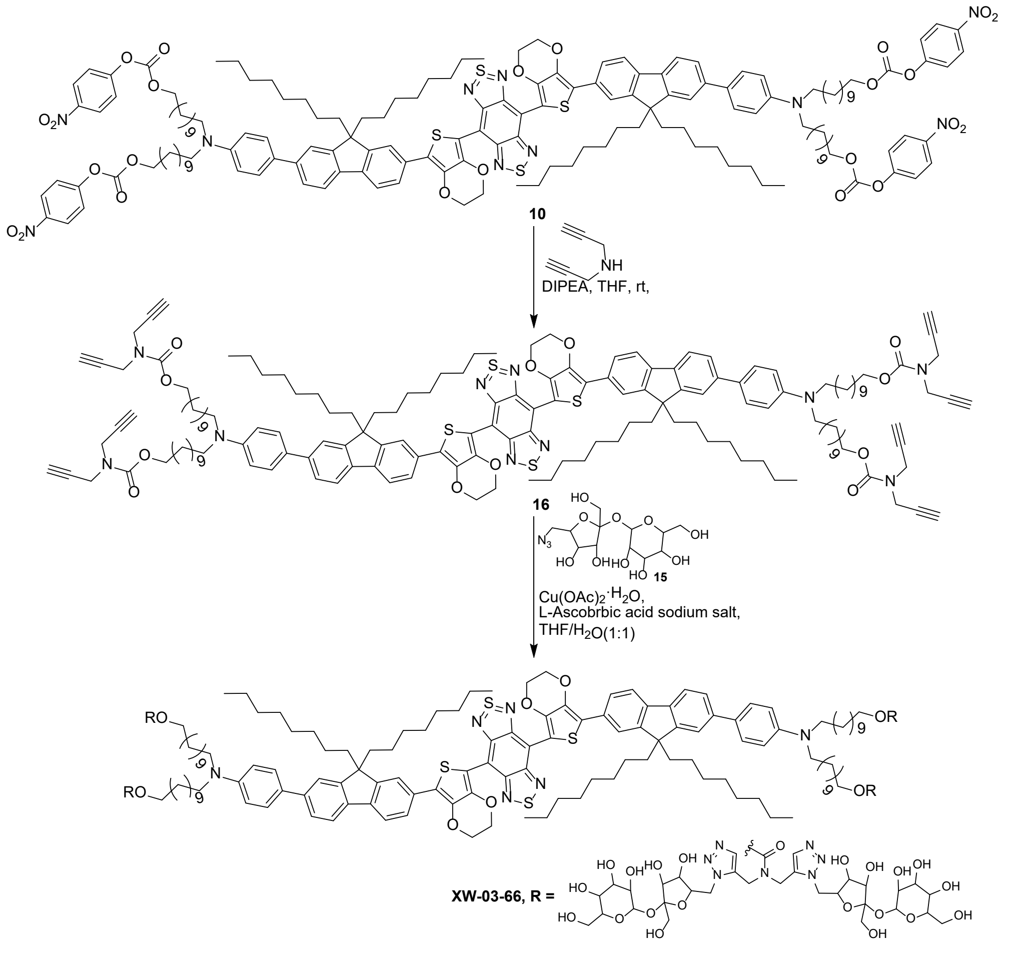

**Scheme S1.3.** Final steps to **XW-03-66**.

**Compound 16**: To a solution of compound **10** (100 mg, 0.04 mmol), pyridine (0.4 ml) in DCM (4 ml) under positive inert gas atmosphere at room temperature, was added *N,N*-diisopropargylamine (0.1 ml) in one portion. After 10 minutes, the reaction was slowly warmed up to room temperature and stirred overnight. The mixture was poured into ice water and extract trice with ethyl acetate. The combined organic phases were washed with saturated $NaHCO_3$, rinsed with brine, dried over $Na_2SO_4$, and the ensuing residue purified by column chromatography to obtain the desired compound **7** as a dark solid (78 mg, 80% yield). $^1$H NMR (600 MHz, $CDCl_3$) δ 7.91 (d, $J$ = 7.8 Hz, 2H), 7.77 (s, 2H), 7.72 (d, $J$ = 7.8 Hz, 4H), 7.57-7.52 (m, 8H), 6.75 (d, $J$ = 7.8 Hz, 4H), 4.53 (s, 4H), 4.40 (s, 4H), 4.25-4.22 (m, 16H), 4.14 (t, $J$ = 6.6 Hz, 8H), 3.32 (s, 8H), 2.25 (s, 8H), 2.05-2.04 (m, 8H), 1.67-1.64 (m, 16H), 1.35-1.31 (m, 66H), 1.12-1.08 (m, 40H), 0.80 (t, $J$ = 7.2 Hz, 12H), 0.73 (s, 8H); $^{13}$C NMR (150 MHz, $CDCl_3$) δ 155.2, 152.6, 151.6, 151.3, 147.4, 141.9, 140.4, 140.2, 138.6, 138.2, 127.8, 125.5, 120.7, 120.4, 119.8, 119.5, 113.1, 111.9, 108.6, 78.4, 73.1, 72.3,

66.4, 64.7, 64.6, 55.2, 51.1, 31.8, 30.1, 29.7, 29.6, 29.5, 29.2, 28.8, 27.3, 27.2, 25.8, 23.8, 22.5, 14.0. HRMS(ESI) calcd for $C_{160}H_{208}N_{10}O_{12}S_4$ ([M+Na$^+$]): 2614.7112, found 2614.7156.

**XW-03-66**: A solution of compound **16** (50 mg, 0.02 mmol), azido sucrose, **15** (114 mg, 0.3 mmol) in THF/methanol/water (2:1:1, 2 mL) was stirred at room temperature for 24 h. The organic solvents were evaporated, and the resulting residue diluted with methanol/water mixture (1:1, 8 mL). This was then loaded into a 3500 MWCO dialysis bag and dialyzed against MES buffer (50 mM, 2×5 L) 4 h and then water (2×5 L) overnight. The water was then removed by freeze drying to obtain the desired compound **XW-03-66** as a dark powder (78 mg, 70% yield). $^1$H NMR (600 MHz, DMSO-$d_6$) δ 7.90-7.78 (m, 22H), 7.63 (s, 6H), 7.56 (s, 12H), 6.70 (s, 4H), 5.52 (s, 8H), 5.15-5.09 (m, 16H), 4.85-4.77 (m, 30H), 4.63-4.31 (m, 32H), 3.95 (s, 24H), 3.78 (s, 24H), 3.52 (s, 32H), 3.30 (s, 24H), 2.99 (s, 8H), 1.99 (s, 8H), 1.52 (s, 8H), 1.35-1.01(m, 88H), 0.85-0.61 (m, 20H). HRMS(MALDI) calcd for $C_{256}H_{376}N_{34}O_{92}S_4$ ([M+H$^+$]): 5527.4750, found 5527.4744.

**Determination of the fluorescence quantum yield.** The quantum yields of **CPK-03-37** and **XW-03-66** in PBS (pH = 7.4) were determined using the dye **IR-26** (Φf = 0.05% in 1,2-dichloroehane) as reference. Briefly, a serial dilution with OD < 0.1 were prepared for **IR-26** (in DCE), **CPK-03-37** (in PBS) and **XW-03-66** (in PBS) and the fluorescent emission spectra were collected following excitation at 782 nm. The integrated emission intensity was plotted as a function of the OD value

$$QY_{sample} = QY_{ref} \times \frac{n^2_{sample}}{n^2_{ref}} \times \frac{Slope_{sample}}{Slope_{ref}} \quad (1)$$

at 782 nm and fitted into a linear function. The different slopes of the linear fit between **IR-26**, **CPK-03-37**, and **XW-03-66** were obtained. The quantum yields were calculated using the following Eq. (1): where $QY_{sample}$ is the quantum yield of **CPK-03-37** or **XW-03-66** in PBS, $QY_{ref}$ is the quantum yield of **IR-26** in DCE, $n_{ref}$ and $n_{sample}$ are the refractive indices of **IR-26** and dyes respectively. $Slope_{sample}$ and $Slope_{ref}$ are the slopes obtained by linear fitting of the integrated emission spectra of **IR-26** and the respective dyes.

**Photostability.** A capillary glass tube filled with 20 µM **XW-03-66** or **ICG** in the respective media. The fluorescence intensity value was measured at different time points under continuous laser illumination for 3 hours using the NIR-II fluorescence imaging system. All imaging experiments were performed in triplicates.

**Intralipid® phantom imaging.** A 1% Intralipid® solution was prepared by diluting 20% Intralipid® (Baxter Healthcare Corp., Deerfield, IL, USA) in deionized water. A cylindrical reservoir filled with 1% Intralipid® solution was positioned on a stage. The height of the stage was adjusted with a micrometer driver (Mitutoyo 151-411ST). A capillary glass tube (OD = 1.55mm/ ID = 1.0 mm) filled with 20 µM solution of **XW-03-66** or **ICG** in PBS was immersed in the Intralipid® solution. The capillary tube was imaged at depths from 0 – 7 mm from the top surface. A Raptor-Ninox 640 SWIR camera (acquired from Phoenix Engineering Inc., Berkeley Lake, GA, USA) was used to acquire images (50 frames at 500 ms each) in the NIR-II window. A long pass 1300 nm filter (Premium Longpass filter, cut-on wavelength 1300 nm, FELH1300, ThorLabs Inc., Newton, NJ, USA) was placed in front of the camera lens. All imaging experiments were performed in triplicates.

**All-atom molecular dynamics simulations.** All-atom molecular dynamics (MD) simulations were performed using the Desmond Schrodinger Package 2021.4 with the OPLS2.1 to OPLS3 and OPLS3e force fields with their usual settings. The Desmond in-built workstations were used to create all the topologies and simulation files. The salt content was fixed to 0.15 M sodium and chloride ions to simulate the physiological composition. An NPT ensemble at 300 K temperature and 1.70 bar pressure was employed. The force field was administered for 500 nanoseconds at a steady rate. The molecular conformations were output at 100 ns intervals up to 500ns.

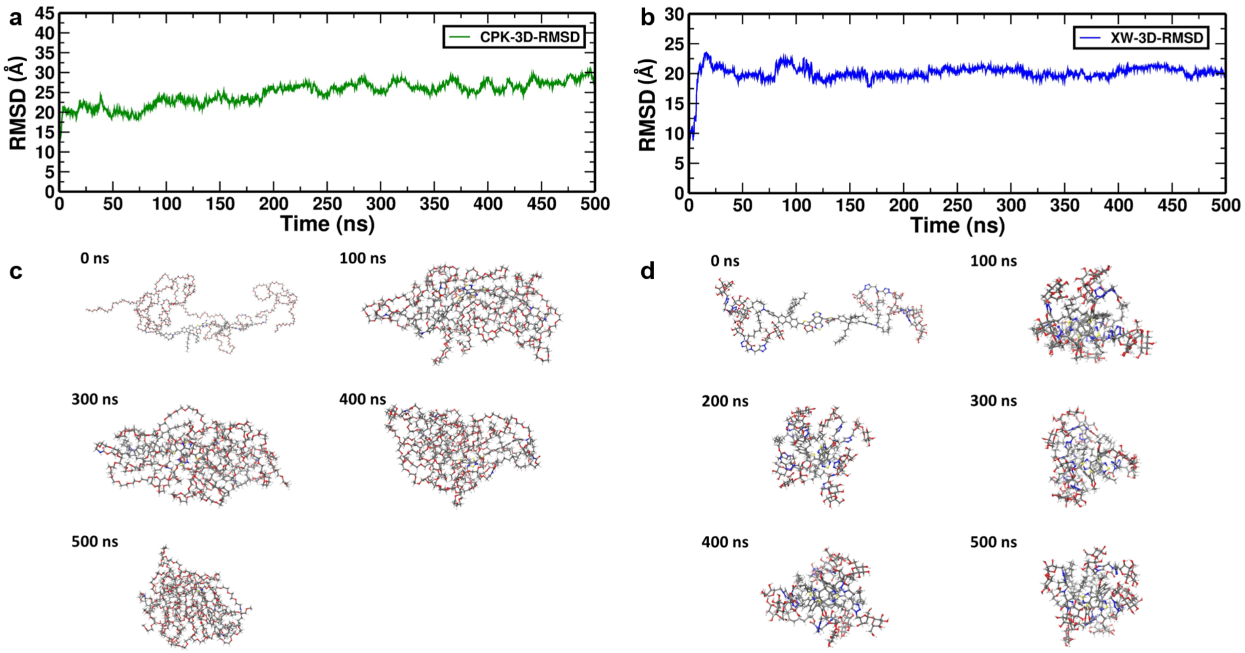


**Figure S.1.1**. Evolutions of the conformations of the **CPK-03-37** and **XW-03-66** dyes over 500 ns of all-atom MD simulations. **a)** and **b)** Root mean square deviations (RMSD) of the distances of all atoms in a conformation at a certain time of the evolution from their respective positions at time 0 ns. **a) CPK-03-37**. **b) XW-03-66**. **c)** and **d)**. Representative conformations at the listed times. **c) CPK-03-37**. **d) XW-03-66**.

**Signal-to-background, resolution using standardized measurements.** Signal-to-background ratio (SBR) was determined by drawing a red dashed line in the region of interest (ROI), and the fluorescence intensity profiles across the line extracted and fitted by Gaussian fitting. The highest intensity on the Gaussian fitted curve is selected as signal ($I_{signal}$) and the intensity of the baseline on Gaussian fitted curve is selected as background ($I_{background}$). SBR is calculated as Eq. (2):

$$\mathrm{SBR} = \frac{I_{\mathrm{signal}}}{I_{\mathrm{background}}} \qquad (2)$$

**Mice**. All animal experiments were conducted under protocols approved by the Institutional Animal Care and Use Committee (IACUC) at Baylor College of Medicine. C57BL/6J male mice at 6-7 weeks were purchased from The Jackson Laboratory and housed under specific pathogen free conditions in standard temperature and lighting conditions. Animals had free access to food and water. Tumor cells were inoculated when mice reached 8-9 weeks of age.

**Tumor models.** mEER and MOC2 tumor cell lines were obtained from Dr. Andrew Sikora's lab at MD Anderson Cancer Center, Houston, TX and maintained in culture as noted previously.[1,2] Briefly, mEER cells were maintained in DMEM: Hams F12: FBS at 67: 22: 10 percentage by volume. The rest of the volume was made up with a 100X antibiotic/antimycotic solution. The following additives, aliquoted from stock solutions was added to make up the complete media: Hydrocortisone at 42.5 ng/mL; Cholera toxin at 8.4 ng/mL; Transferrin at 5 µg/mL; Insulin at 5 µg/mL; Tri-iodo-thyronine at 1.4 ng/mL; and EGF at 5 ng/mL. MOC2 cells were maintained in IMDM: Hams F12: FBS at 63: 31: 5 percentage by volume. The rest of the volume was made up with a 100X antibiotic/antimycotic solution. The following additives, aliquoted from stock solutions was added to make up the complete media: Hydrocortisone at 40 ng/mL; Insulin at 5 µg/mL; and EGF at 5 ng/mL. The cells were maintained at 37 $^oC$ in 5% $CO_2$. For tumor inoculation, cells at 70-80 % confluency were detached from flasks using Trypsin. Cells were spun at 70-80 g's, and pellet was resuspended in cold PBS to obtain homogenous cell suspension. 100 µL of cells in PBS was injected into the subcutaneous space on the right flank of mice. Approximately $1x10^6$ mEER cells and $1x10^5$ MOC2 cells were injected.

***In vivo* NIR-II Vascular Imaging and Biodistribution Studies.** *In vivo* vascular imaging and biodistribution studies imaging were performed in nude mice. Animals were anesthetized using

2% isoflurane in an induction chamber followed by maintenance on 1–1.5% isoflurane delivered using a nose cone setup. The tail vein was catheterized for injection of **ICG** or **XW-03-66** (dosage, 10 mg/kg for vascular imaging, pharmacokinetics (nude and C57BL/6J mice), and toxicity studies (C57BL/6J mice), and 5 mg/kg for tumor imaging). The animals were secured to a platform in the supine position, with the abdomen facing the camera and laser source. All NIR-II images were collected using A Raptor-Ninox 640 SWIR camera. The excitation laser was set at 785 nm and images collected with 1300 nm long-pass filter. Exposure time for all images was 500 ms. Images and movies were processed with FIJI ImageJ software.

**Preoperative MRI Imaging**

Magnetic resonance imaging (MRI) was performed on a 1.0 T permanent MRI scanner (M2 system, Aspect Imaging, Shoham, Israel) with a 35 mm volume coil. Mice were sedated with 2-3% isoflurane, placed on the MRI animal bed, and then maintained under anesthesia at 1.5-2.5% isoflurane with a nose cone setup. Body temperature was maintained by circulating hot water through the MRI animal bed. Respiration rate was monitored using a pneumatically controlled pressure pad placed in the abdominal area underneath the animal. Tumor imaging was performed using a T2-weighted fast-spin echo (FSE) sequence with the following scan parameters: echo time (TE) = 80 ms, repetition time (TR) = 3030 ms, slice thickness = 1.2 mm, field of view = 80 mm, number of slices = 16, matrix = 256 x 250, no. of signal averages = 2, dwell time = 25 μs, scan time ~2.5 min. Images were analyzed and processed in Osirix (version 5.8.5, 64-bit, Pixmeo, Bernex, Switzerland)

***In vivo* tumor imaging and *ex-vivo* image guided tumor resection.** Animals were anesthetized using 2% isoflurane in an induction chamber followed by maintenance on 1 – 1.5% isoflurane delivered using a nose cone setup. The tail vein was catheterized for injection of **XW-03-66** or **ICG** (5 mg/kg). The animals were secured to a platform in the prone position, with the tumor

facing the camera and laser source and images captured as desired at different time points. Prior to tumor resection, animals were euthanized in a $CO_2$ chamber

**Inflammatory potential.** (ELISA MAX™ Deluxe Set Human TNF-α; ELISA MAX™ Deluxe Set Human IL-1β; ELISA MAX™ Deluxe Set Human IL-6; Biolegend).
(ELISA MAX™ Deluxe Set Mouse TNF-α; ELISA MAX™ Deluxe Set Mouse IL-1β; ELISA MAX™ Deluxe Set Mouse IL-6, Biolegend). Experiments were perfumed described in the technical data sheet.

**Cytotoxicity.** The human microglia clone 3 cell line (HMC-3) was purchased from American Type Culture Collection (ATCC, Manassas, VA, USA). $HMC_3$ cells were cultured in Eagle's Minimum Essential Media (EMEM, ATCC)) supplemented with 10% FBS (Sigma-Aldrich, St. Louis, MO, USA) and 100 units/mL (U/mL) penicillin/streptomycin (Invitrogen, Carlsbad, CA, USA), and incubated in a humidified atmosphere (5% $CO_2$) at 37 ºC.
HUVECs, SHSY5Y and SIM9A were purchased from American Tissue Culture Collection (ATCC, Manassas, VA, U.S.A.) and cultured in Dulbecco's modified Eagle's medium (DMEM, Thermo Fisher Scientific, Waltham, MA, U.S.A.) with 10% fetal bovine serum (FBS, Thermo Fisher Scientific) in an atmosphere of 5% $CO_2$ at 37 ºC.
The mouse mononuclear macrophage leukemia cell line RAW264.7 was purchased from the American Type Culture Collection (ATCC). Cells were cultured in DMEM containing 10% fetal bovine serum (FBS, Gibco; Thermo Fisher Scientific) and 1% penicillin (100 U/ mL)/streptomycin (100 μg/mL) (Hyclone; GE Healthcare Life Sciences) in a 37 ºC in an incubator containing 5 % $CO_2$ at 37 ˚C.
Kupffer cells was purchased from the American Type Culture Collection (ATCC) and cultured in Roswell Park Memorial Institute 1640 medium (Thermo Fisher Scientific, Waltham, MA, U.S.A.) with 10% fetal bovine serum (FBS, Thermo Fisher Scientific) in an atmosphere of 5% $CO_2$ at 37˚C.
Cytotoxicity was evaluated using an alamarblue (Sigma-Aldrich) assay. Cells were plated into a 96 well plate at a concentration of 15000 cells/well in medium with 10% fetal bovine serum (FBS)

at 37 ºC and 5% $CO_2$, and 24 h later, the cells were incubated with **XW-03-66** at different concentrations (0 μg/mL, 0.3 μg/mL, 1 μg/mL, 3 μg/mL, 10 μg/mL, 30 μg/mL, 100 μg/mL, 300 μg/mL, 1000 μg/mL) for 24 h. Taking out old medium after 24 h. Then medium/alamarblue (10:1) fresh solution was added and incubated for 2-3 h. The absorbance (A) at a wavelength of 570 nm was measured with a Bio-Rad microplate reader. The relative cell viability (%) was calculated by $(A_{sample} / A_{control}) \times 100$ %. All samples were done in triplicate and the experiment was replicated three times.

**Histopathologic studies.** All tissues were fixed with 4% paraformaldehyde (diluted from 10% paraformaldehyde with PBS), dehydrated in a series of ethanol solution, embedded in paraffin and cut into sections with a thickness of 15 μm for H&E staining. The sections were washed with xylene and ethanol and then immersed in hematoxylin working solution for 4 min and eosin working solution for 2 min, followed by washing with distilled water. The sections were examined using a Nikon ECLIPSE Ni microscope.

**Statistical Analysis**. Preliminary toxicity panels (TNF-α, IL-1β, and IL-6) in Kupffer cells exposed to **XW-03-66** were acquired without pre-processing (Figure 6a). All samples were tested in triplicate (n = 3) and data are presented as mean (bar plot) and standard deviation (error bars). Statistical analysis was performed using a paired two-sided t-Test with an alpha = 0.05 and 2 degrees of freedom. This statistical analysis is repeated for Supplementary Figure S1.9. In other figures within this manuscript that do not contain statistical analysis, error bars denote standard deviation.

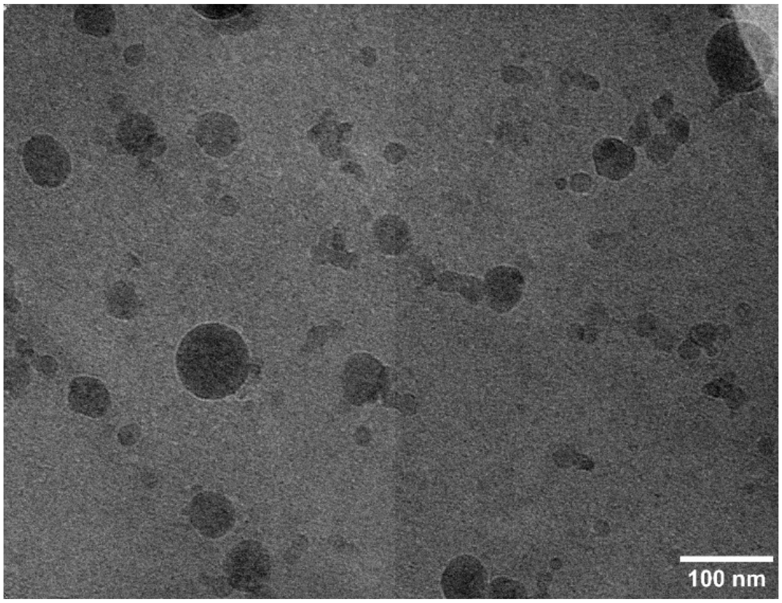


**Figure S1.2.** Cryo-electron microscopy image showing particle distribution in a 50 μM solution of **XW-03-66.**

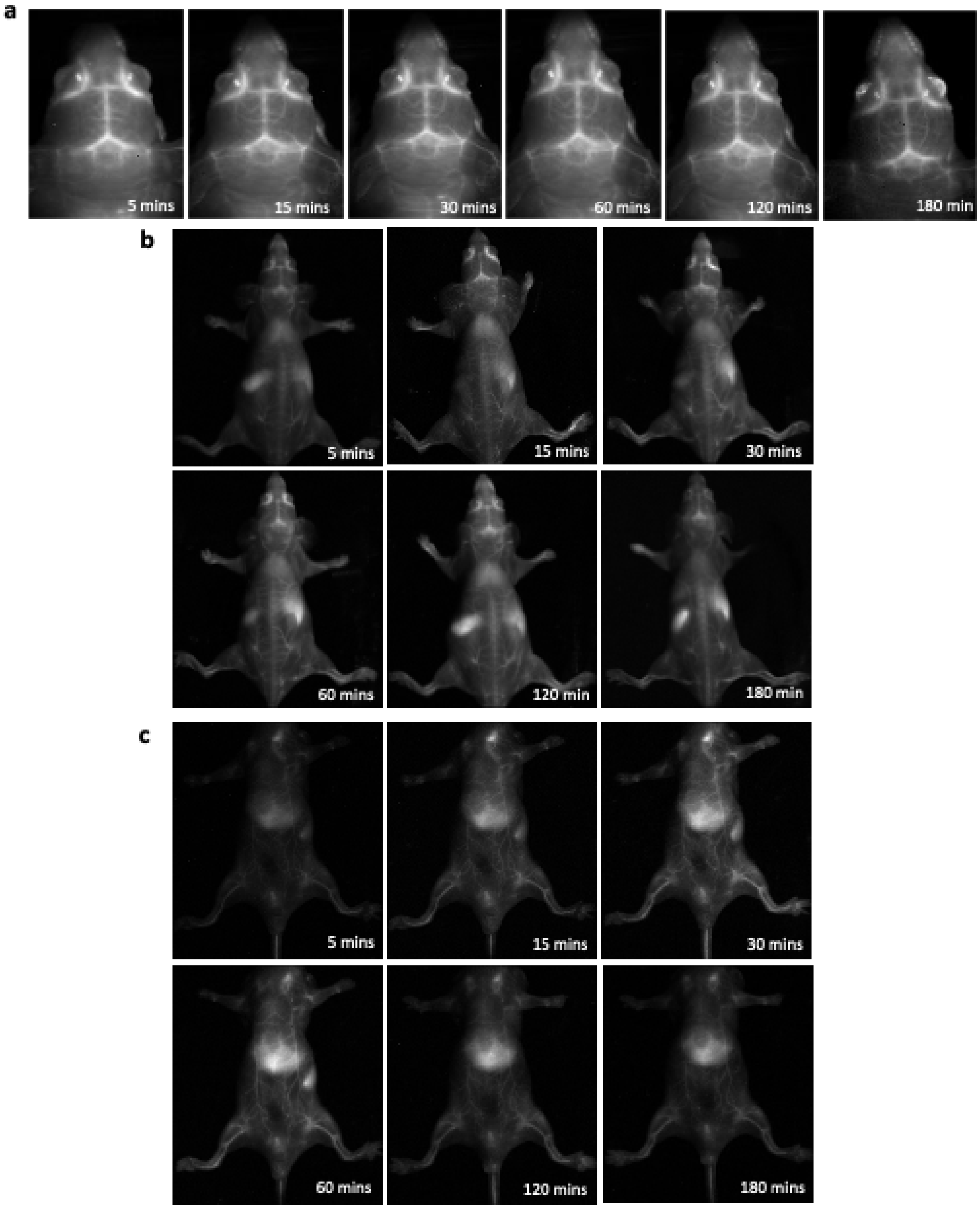


**Figure S1.3**. The long circulation and favorable *in vivo* fluorescence properties of **XW-03-66** allow for the capture of high-resolution vascular images over a 6 h period following i.v. administration. **a**) Cranial vasculature; **b**) Dorsal vasculature; **c**) Abdominal and thigh vasculature.

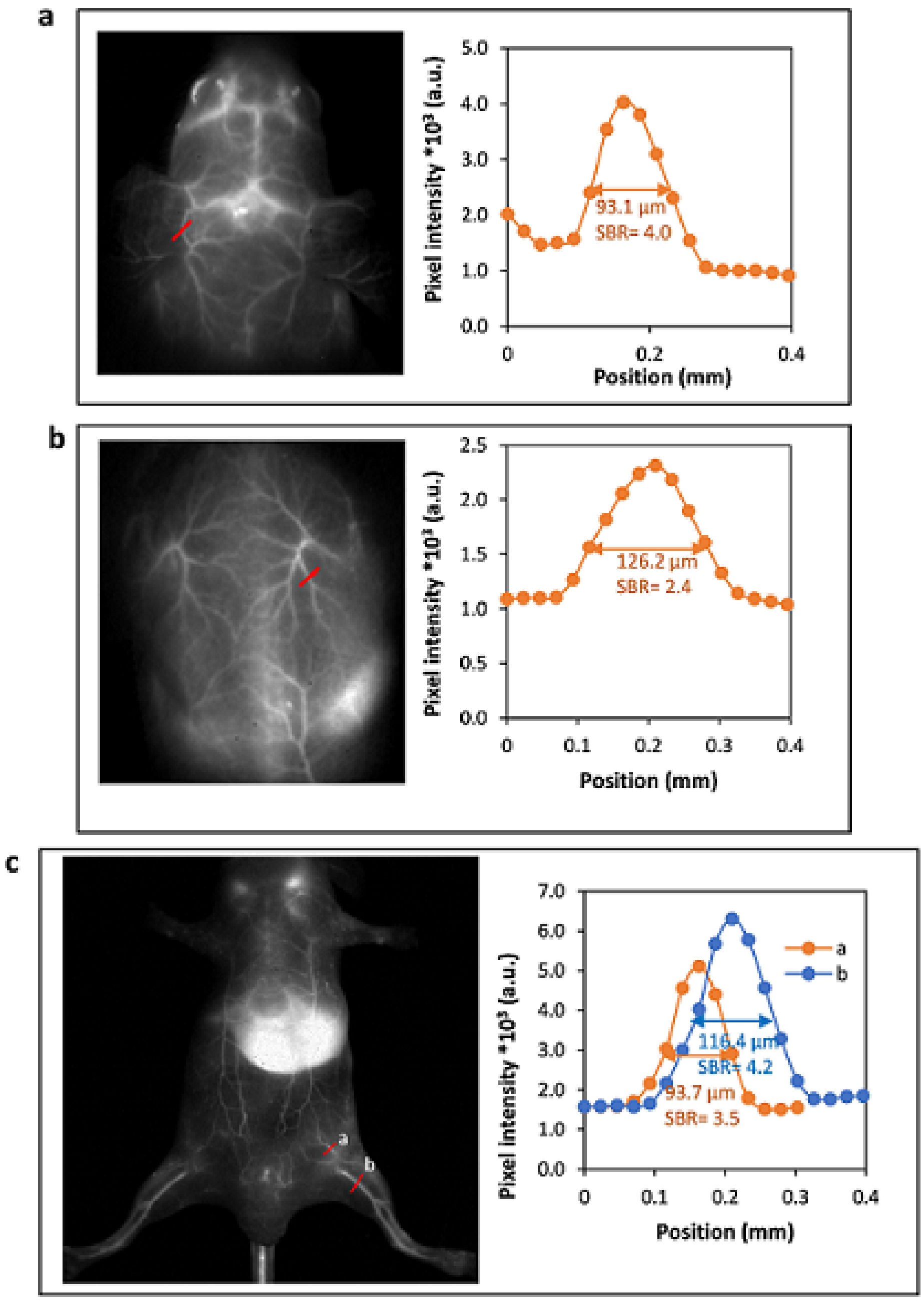


**Figure S1.4.** Fluorescence intensity profile (dots) and Gaussian fit (line) along the red line drawn across vessels. a) Head; b) Back; c) Thigh.

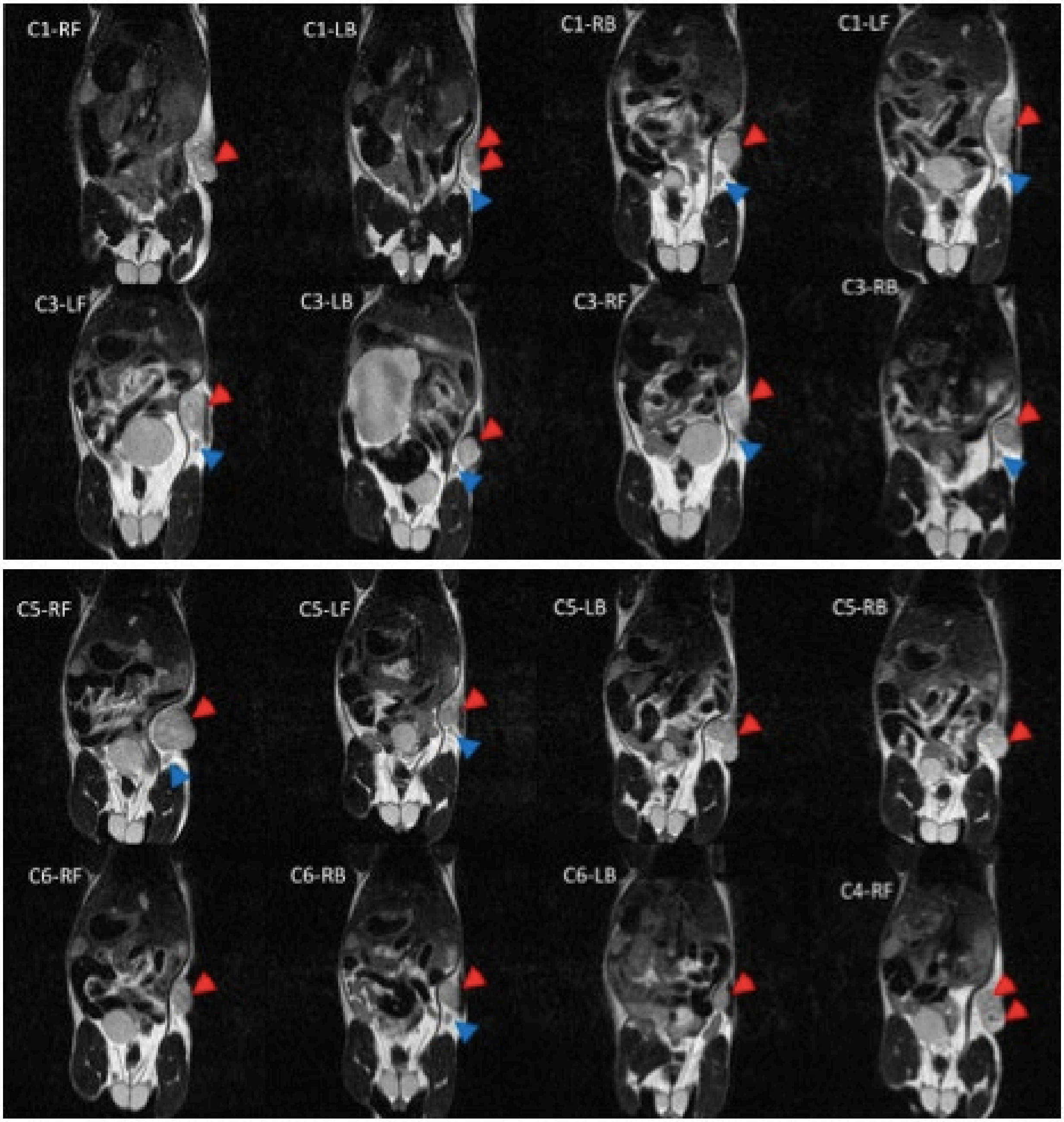


**Figure S1.5.** Preoperative MRI confirms tumor (red arrow heads) and inguinal lymph node (blue arrow heads) in both MOC2 and mEER mouse models.

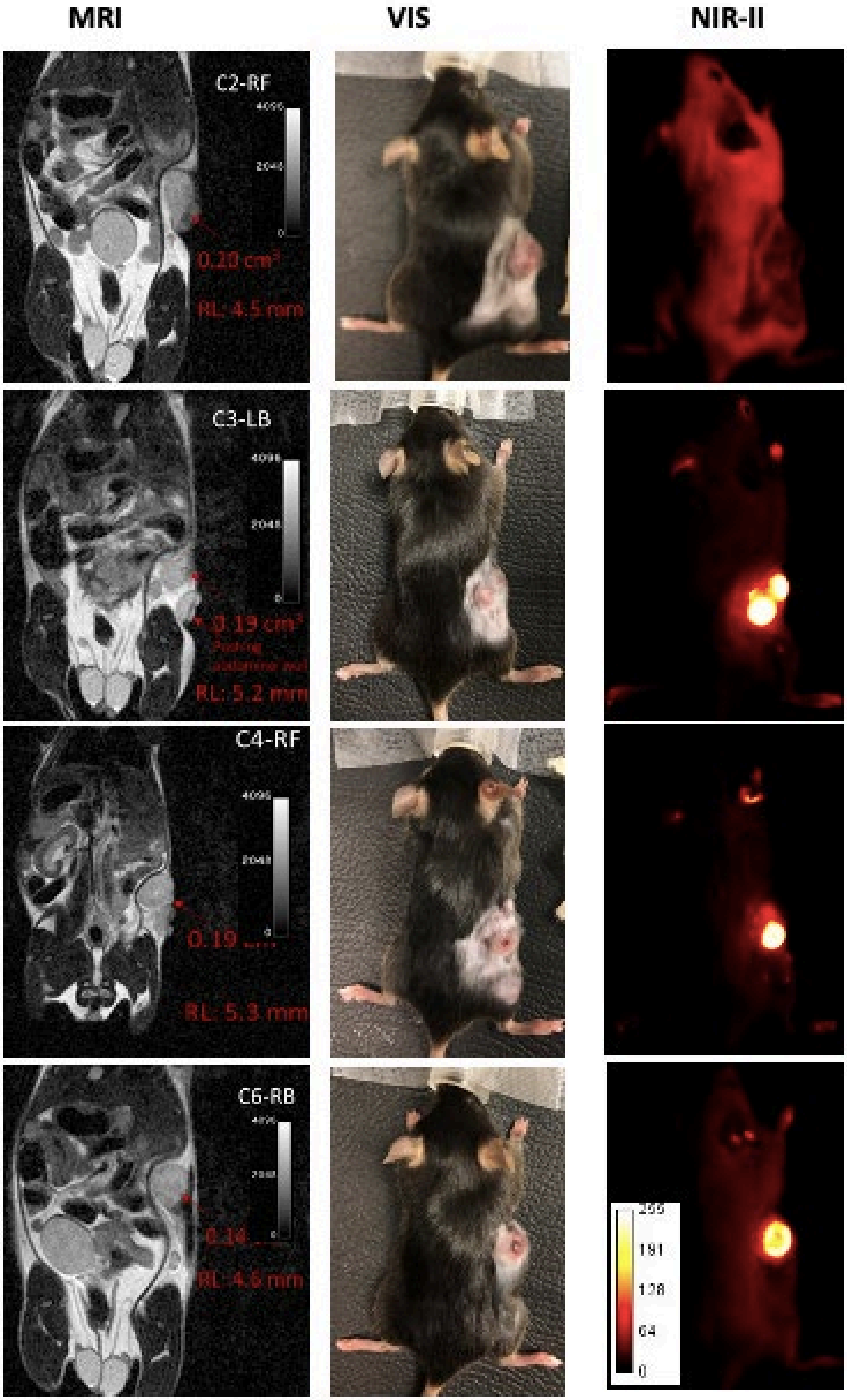


**Figure S1.6.** Gallery of MR, VIS, and NIR-II images of MOC2 tumors.

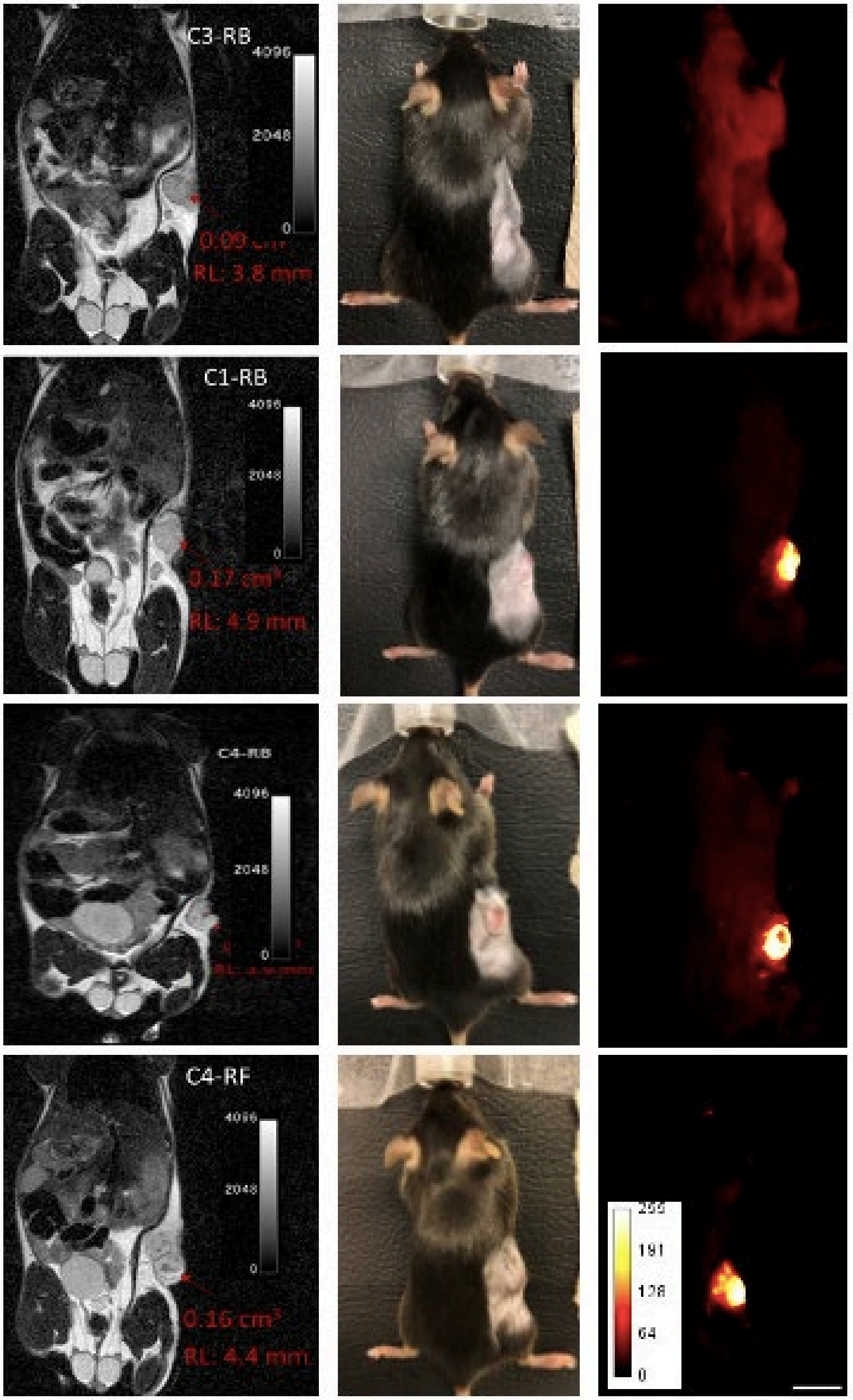


**Figure S1.7.** Gallery of MR, VIS, and NIR-II images of mEER tumors.

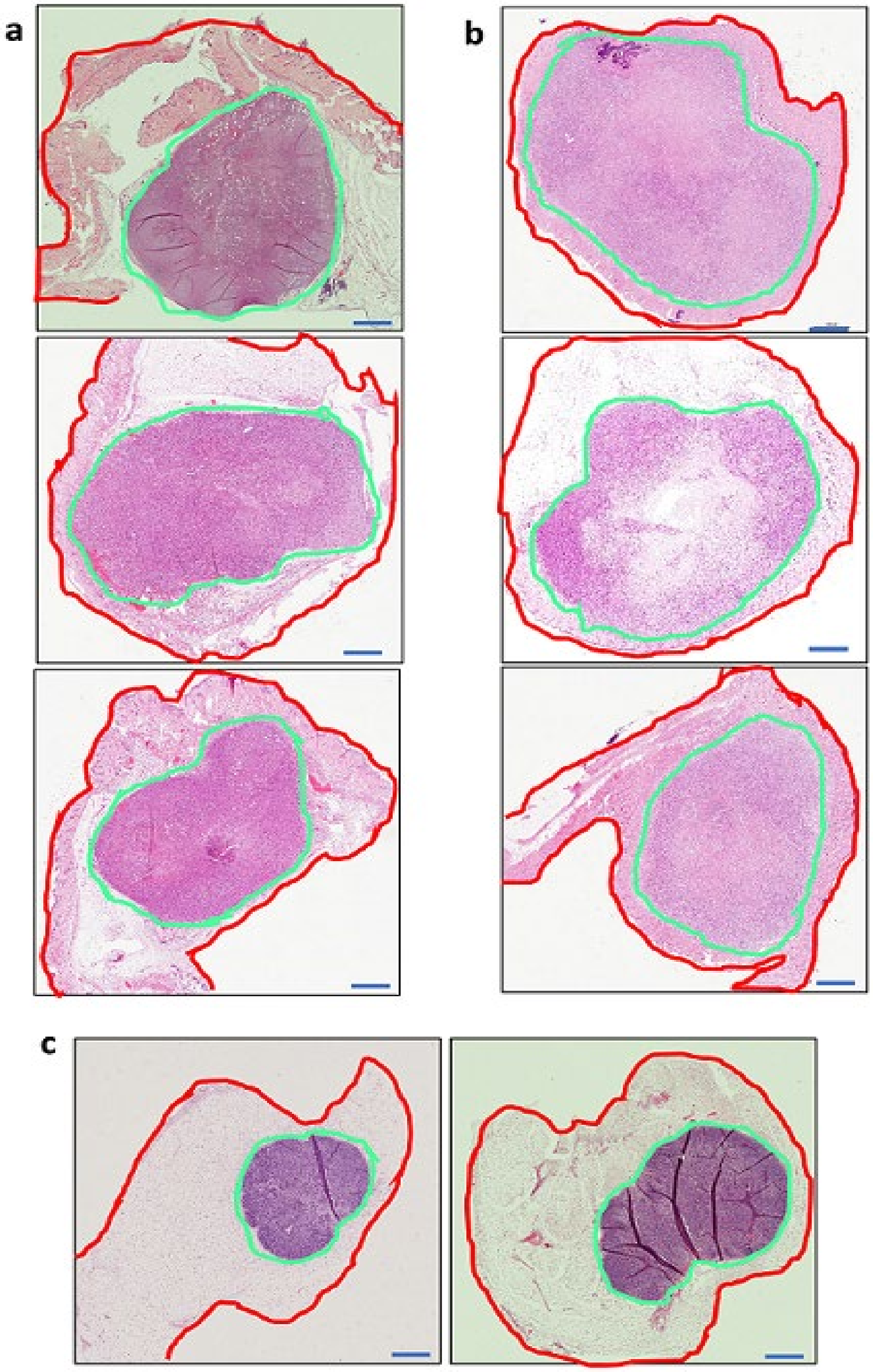


**Figure S1.8**. H&E images of showing successful removal of tumors and lymph nodes with negative resection margins (red lines) while leaving tumor/lymph node boundaries (green lines) unscathed. A) MOC2 tumors; b) mEER tumors; c) Lymph nodes. Scale bar = 1000 µm.

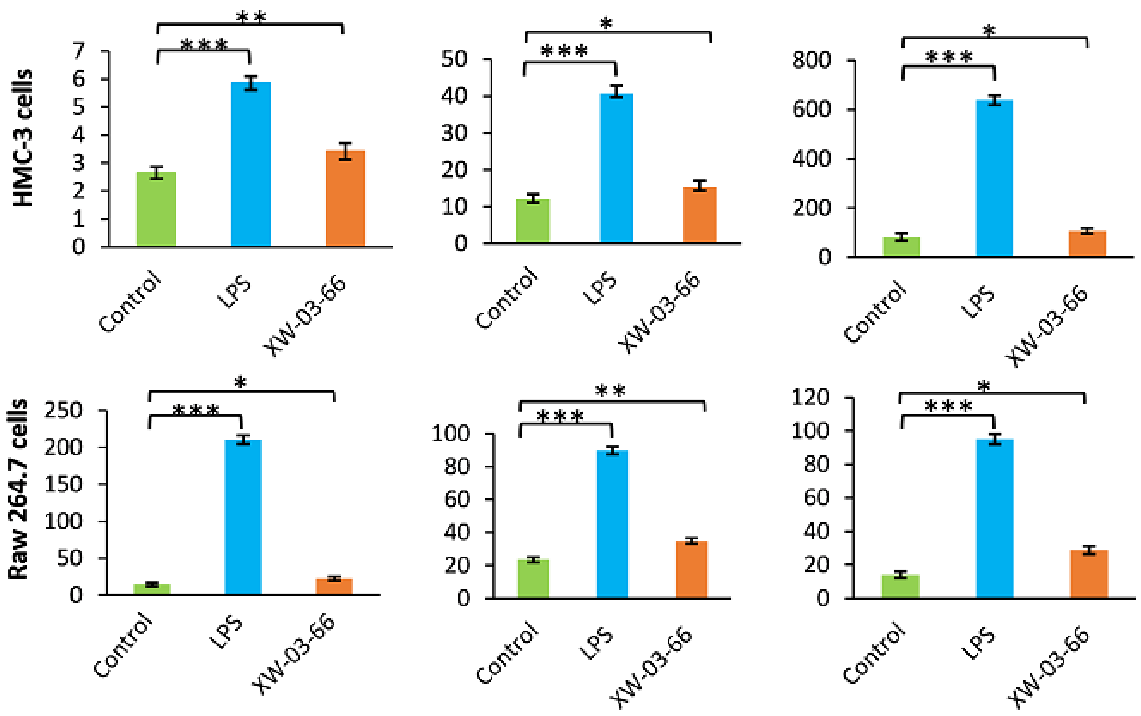


*P <0.05, **p < 0.01, ***p < 0.001

**Figure S1.9**. Evaluation of three different inflammation markers following exposure of **XW-03-66** in microglia (HMC-3) and macrophage Raw 264.7) cell lines suggest that the dye has a low inflammatory potential.

**References**.

[1] W. C. Spanos, P. Nowicki, D. W. Lee, A. Hoover, B. Hostager, A. Gupta, M. E. Anderson, J. H. Lee. *Arch Otolaryngol Head Neck Surg.* 2009;135(11):1137–46

[2] N. P. Judd, C. T. Allen, A. E. Winkler, R. Uppaluri, *Otolaryngol. Neck Surg.* **2012**, *147*, 493.

.
