# Supplementary material for: Rational Design of a Self‐Assembling High Performance Organic Nanofluorophore for Intraoperative NIR‐II Image‐Guided Tumor Resection of Oral Cancer
Source: Adv Sci (Weinh). 2023 Jan 31;10(10):2206435. doi: 10.1002/advs.202206435 (PMC10074073; doi:10.1002/advs.202206435)
Supplement: Supplementary file 2 — Supporting Information 2 [file ADVS-10-2206435-s001.pdf]

ADVANCED SCIENCE
Open Access

Supporting Information

for *Adv. Sci.*, DOI 10.1002/advs.202206435

Rational Design of a Self-Assembling High Performance Organic Nanofluorophore for Intraoperative NIR-II Image-Guided Tumor Resection of Oral Cancer

*Xianwei Sun, Praveen Kumar Chintakunta, Andrew A. Badachhape, Rohan Bhavane, Huan-Jui Lee, David S. Yang, Zbigniew Starosolski, Ketan B. Ghaghada, Peter G. Vekilov, Ananth V. Annapragada and Eric A. Tanifum**

Supporting information 2 (S2): NMR, Mass Spectra, and Profiles of XW-03-66 and CPK-03-37 Solutions

Rational Design of a self-assembling high performance organic nanofluorophore for intraoperative NIR-II image-guided tumor resection in mouse models of oral cancer

*Xianwei Sun[1], Praveen Kumar Chintakunta[1,2], Andrew Badachhape[1,3], Rohan Bhavane[1,3], Huan-Jui Lee[4], David S. Yang[4], Zbigniew Starosolski[1,3], Ketan Ghaghada[1,3], Peter Vekilov[4,5], Ananth Annapragada[1,3], and Eric Tanifum[1,3]**

[1]Department of Radiology, Baylor College of Medicine, Houston, TX 77030, USA.

[2] Current Address: Sai Life Sciences Ltd, Turakapally, Telangana, India.

[3]Department of Radiology, Texas Children's Hospital, Houston, TX 77030, USA.
[4]Department of Chemical and Biomolecular Engineering, University of Houston, Houston, TX 77204, USA.
[5]Department of Chemistry, University of Houston, Houston, TX 77204, USA

*E-mail: eatanifu@texaschildrens.org

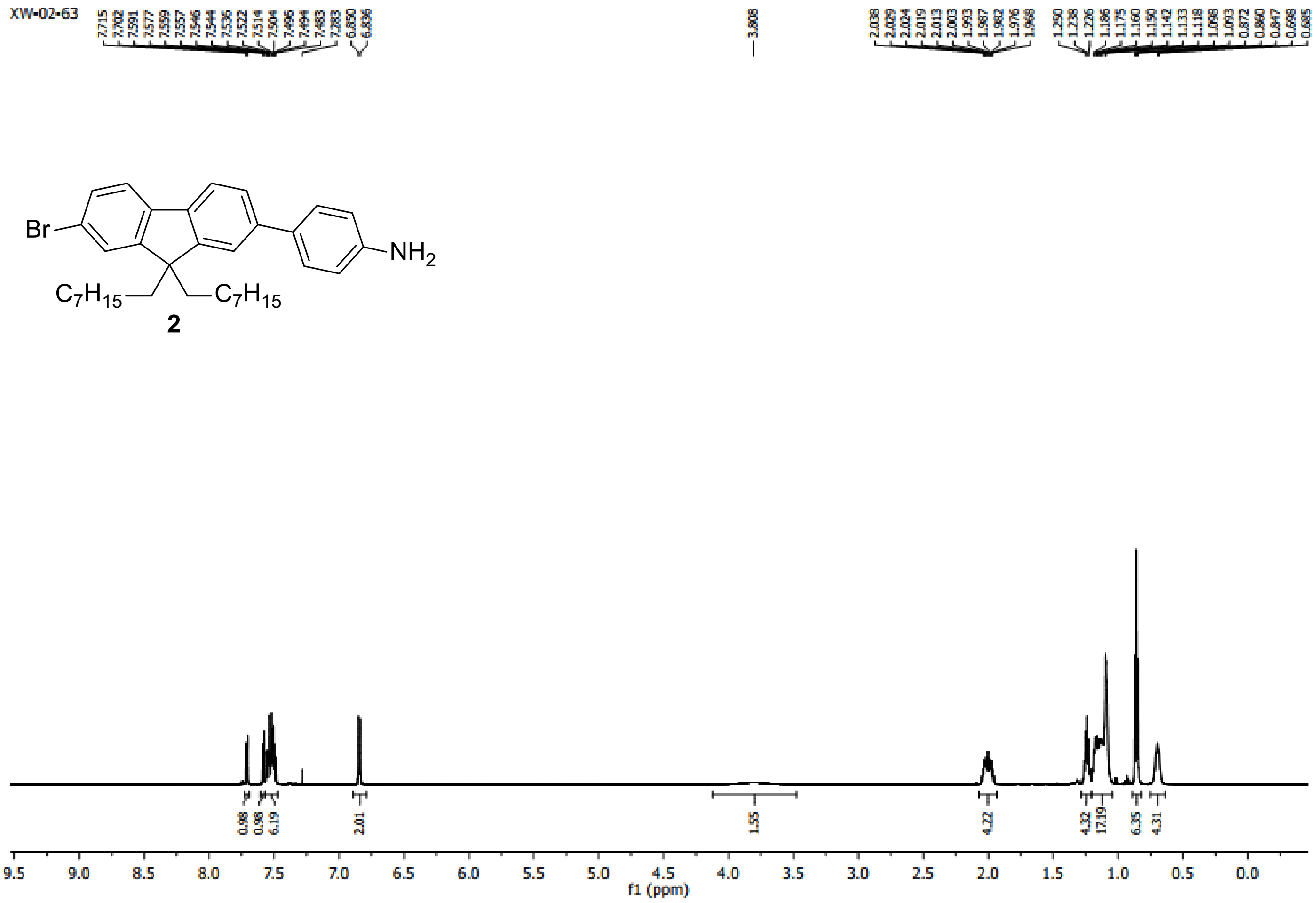

Figure S2.1. ^{1}H NMR Spectrum of Compound **2**

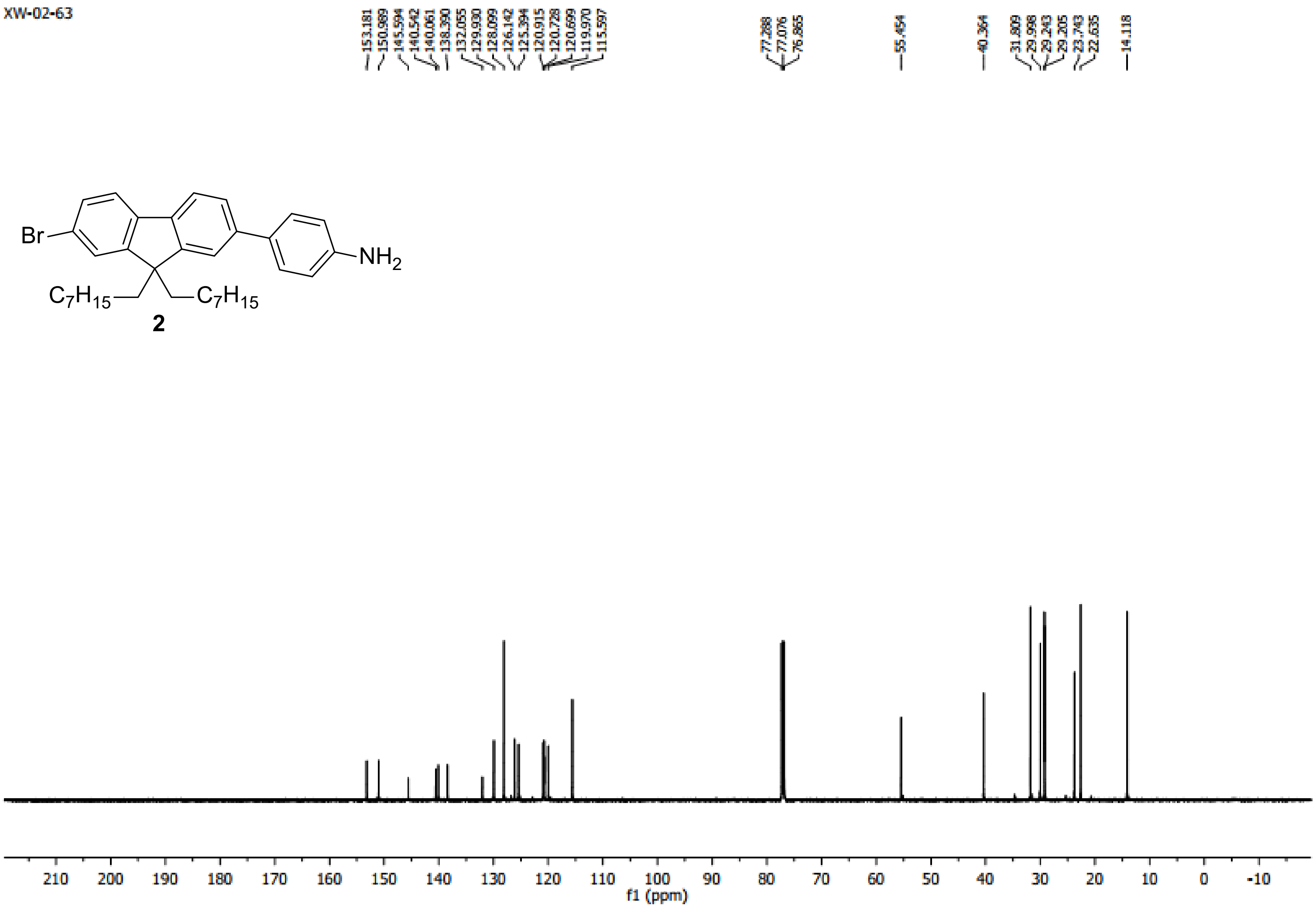

Figure S2.2. ^{13}C NMR Spectrum of Compound **2**

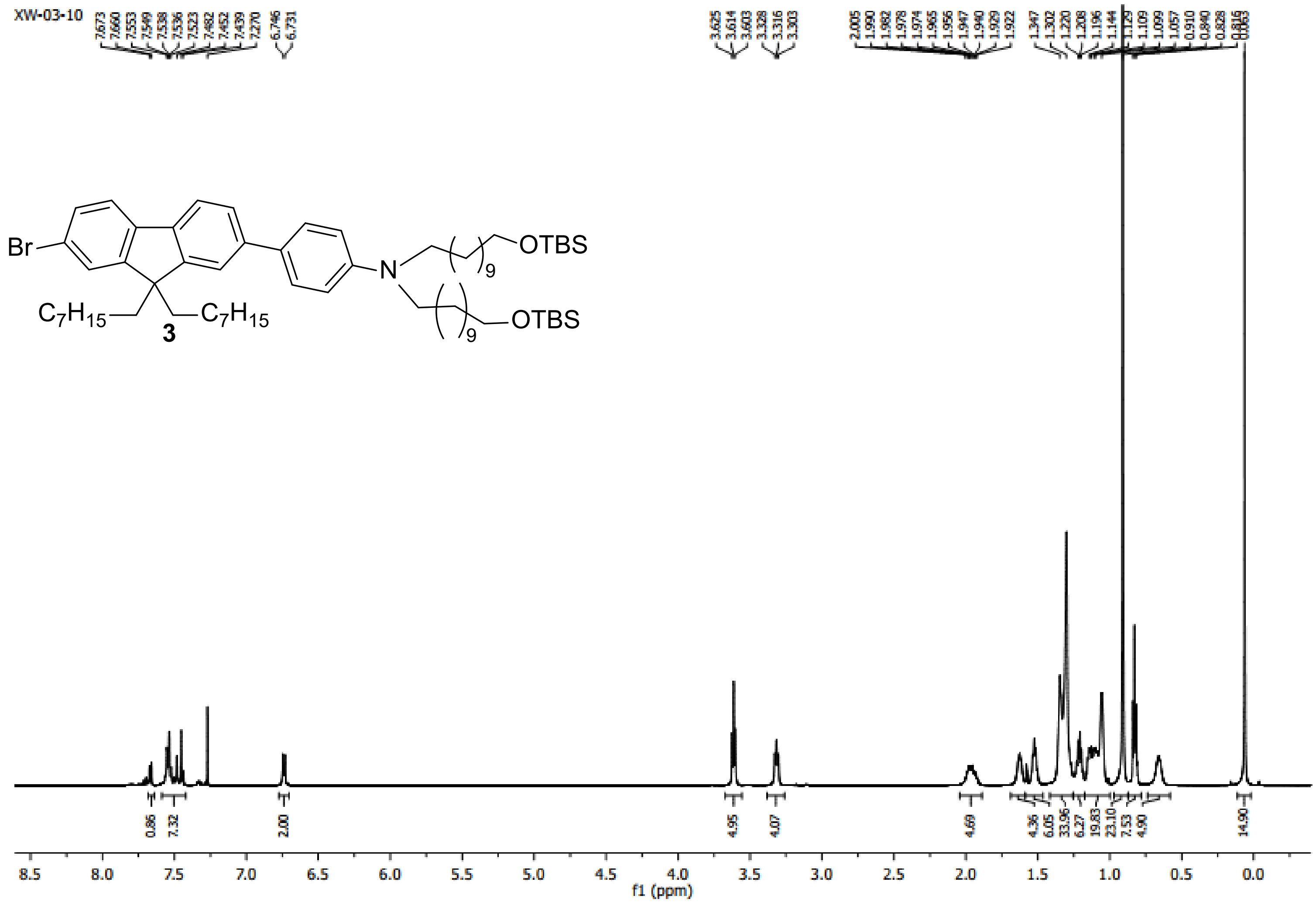

Figure S2.3. ^{1}H NMR Spectrum of Compound **3**

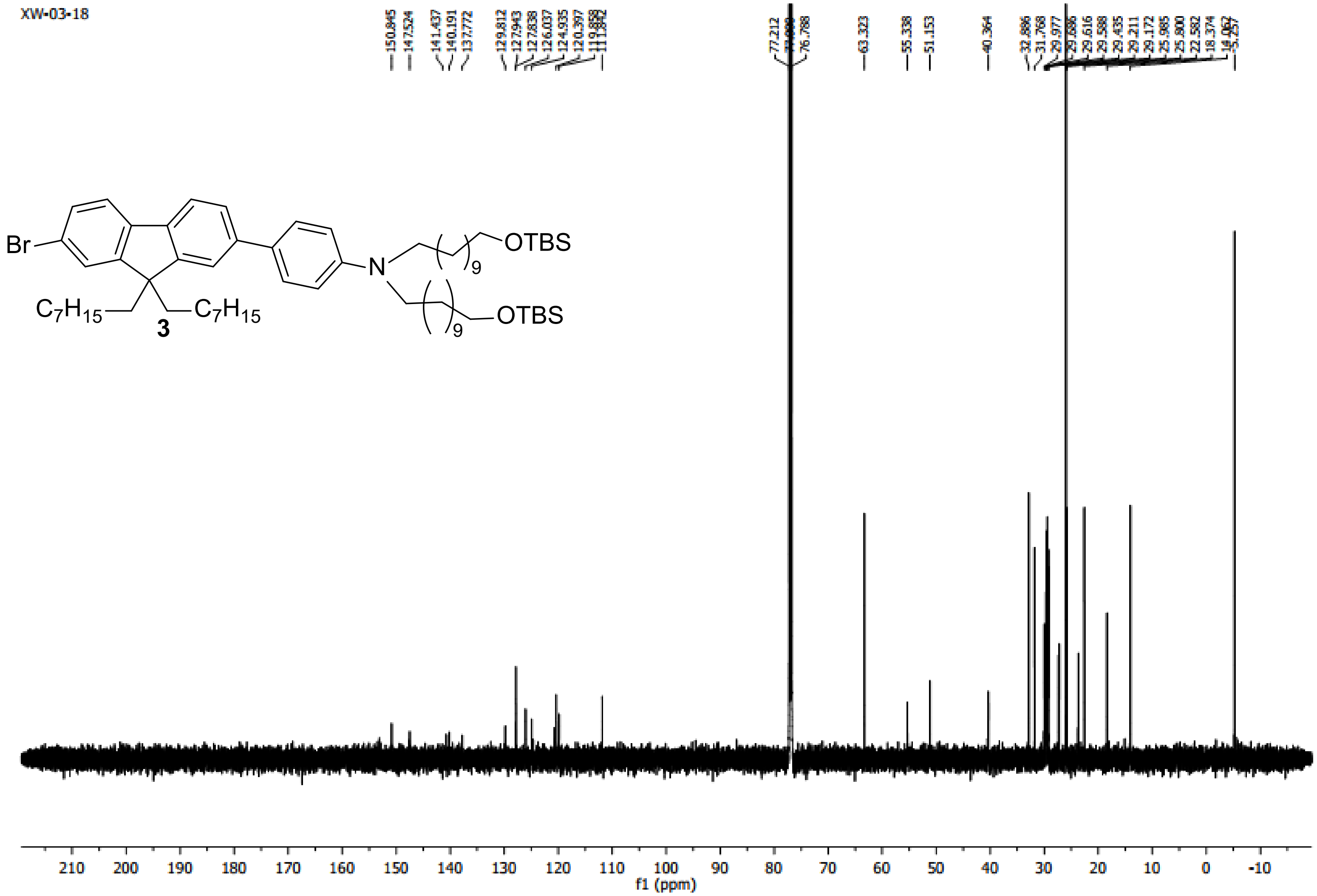

Figure S2.4. ^{13}C NMR Spectrum of Compound **2**

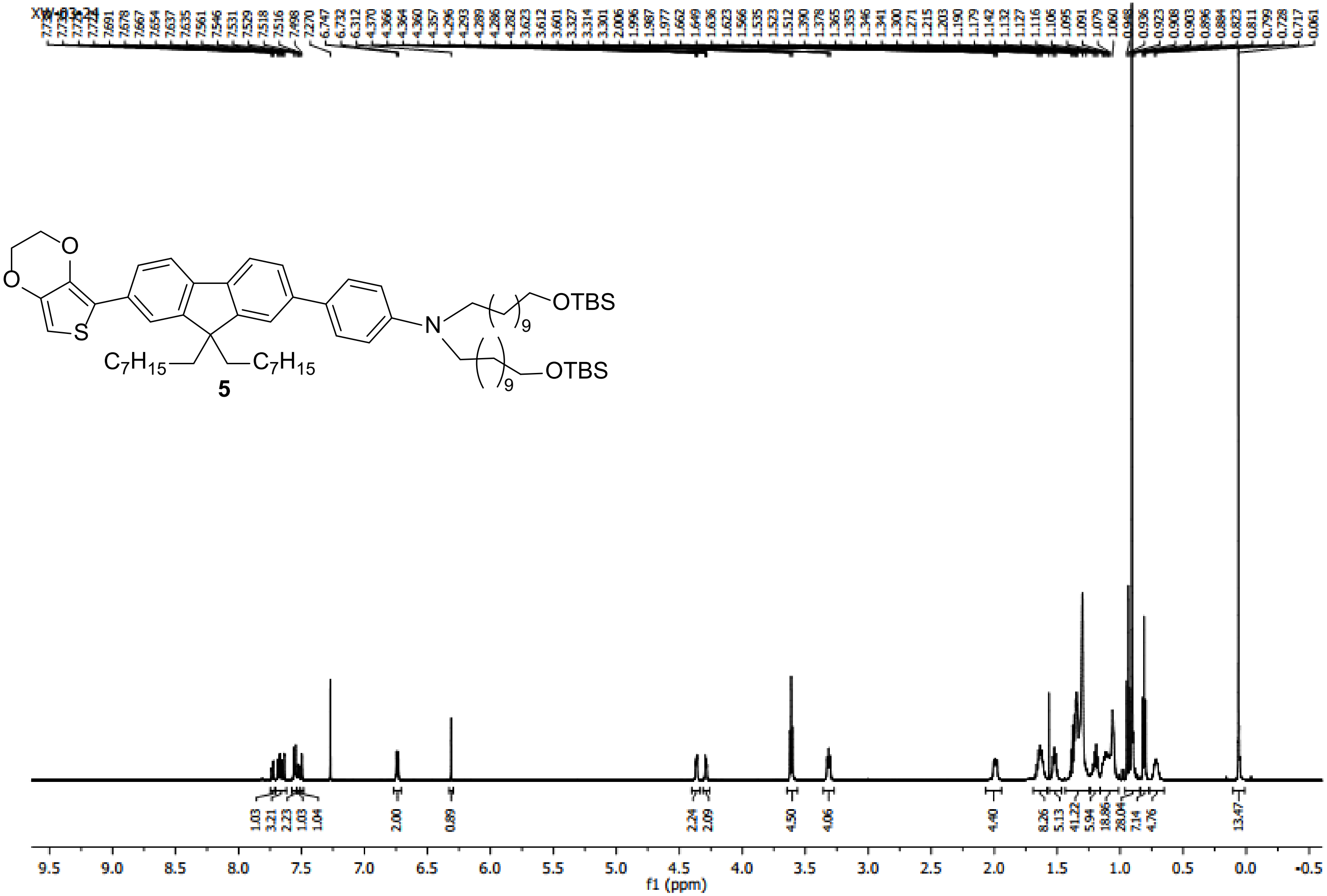

Figure S2.5. ^{1}H NMR Spectrum of Compound **5**

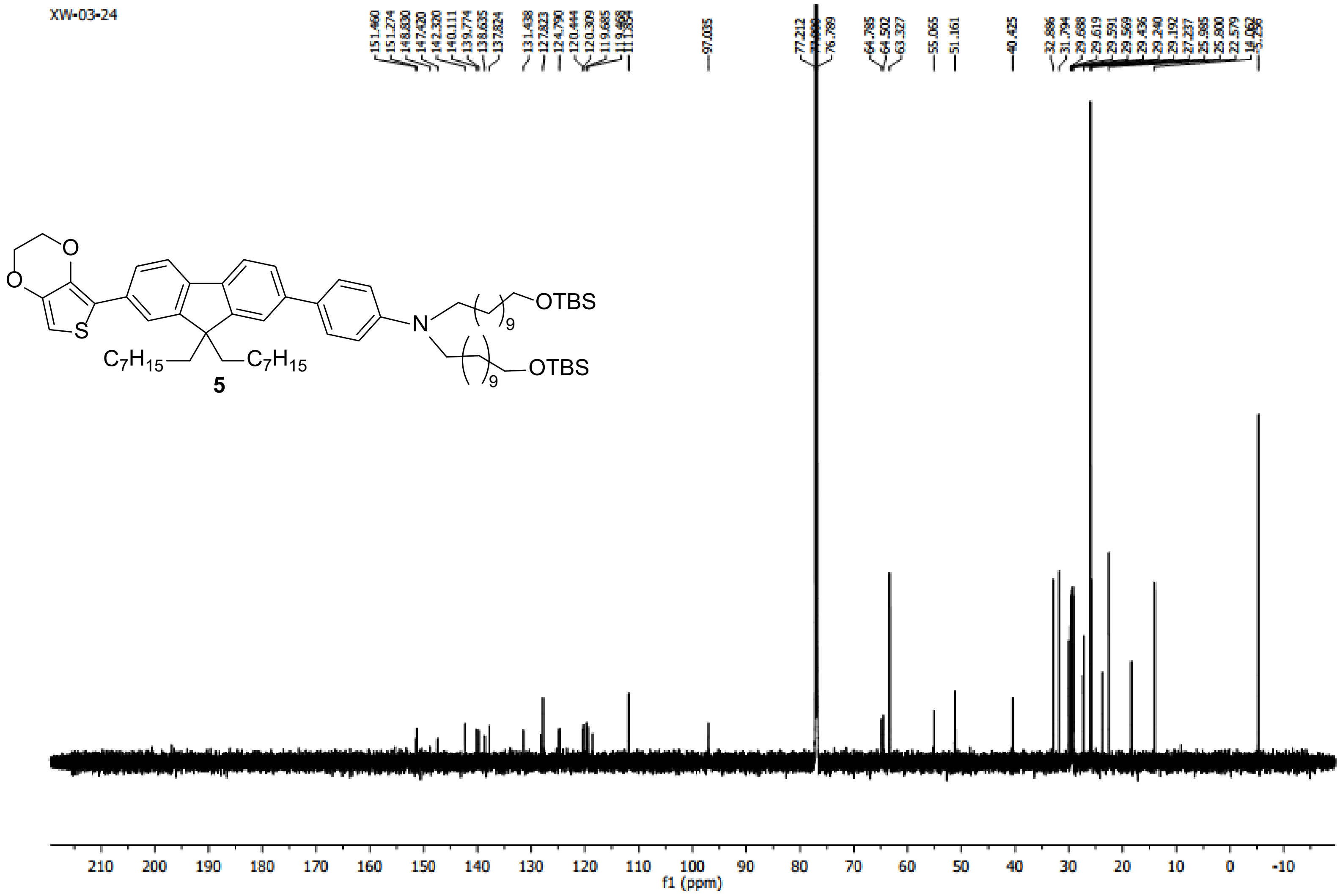

Figure S2.6. ^{13}C NMR Spectrum of Compound **5**

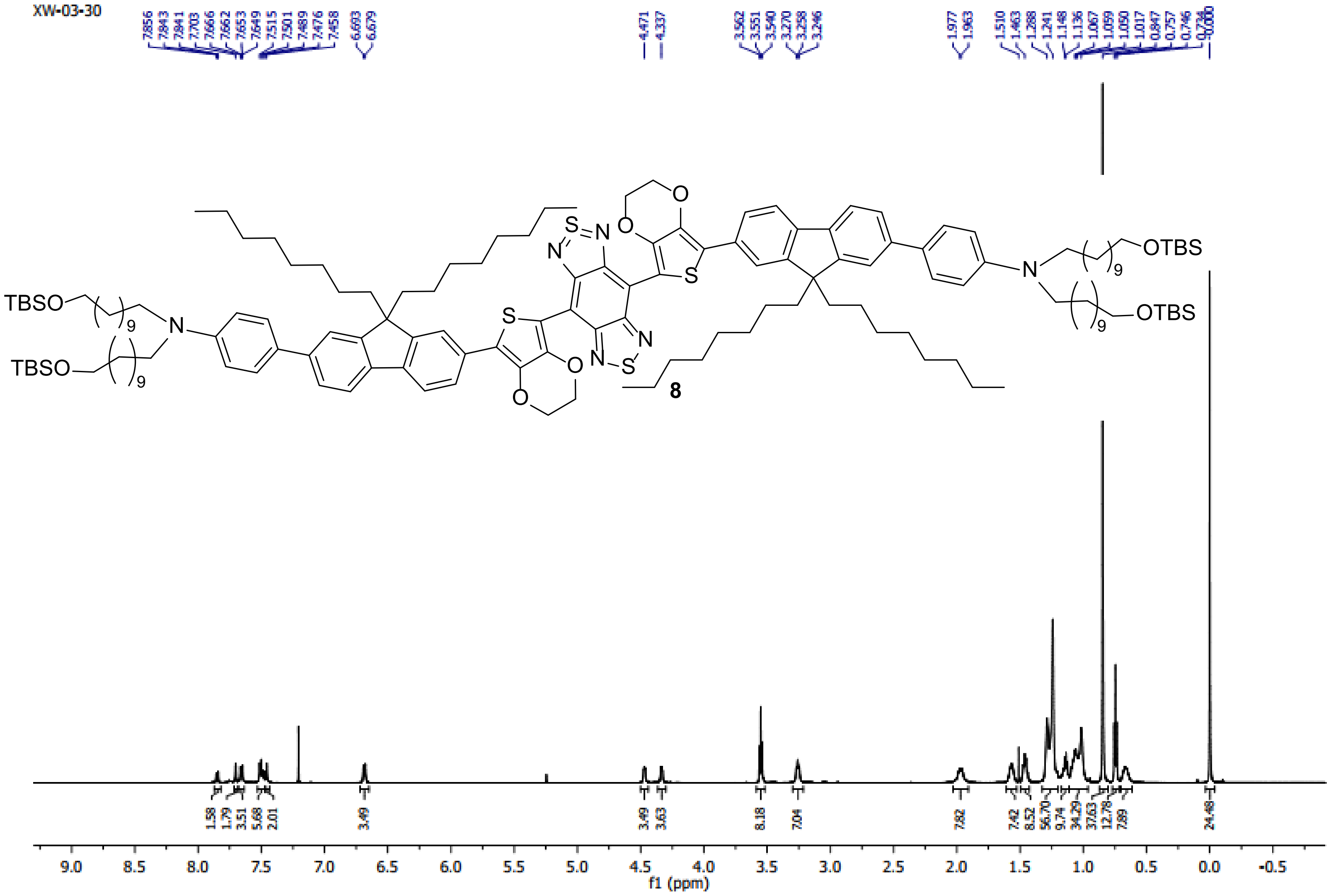

Figure S2.7. ^{1}H NMR Spectrum of Compound **8**

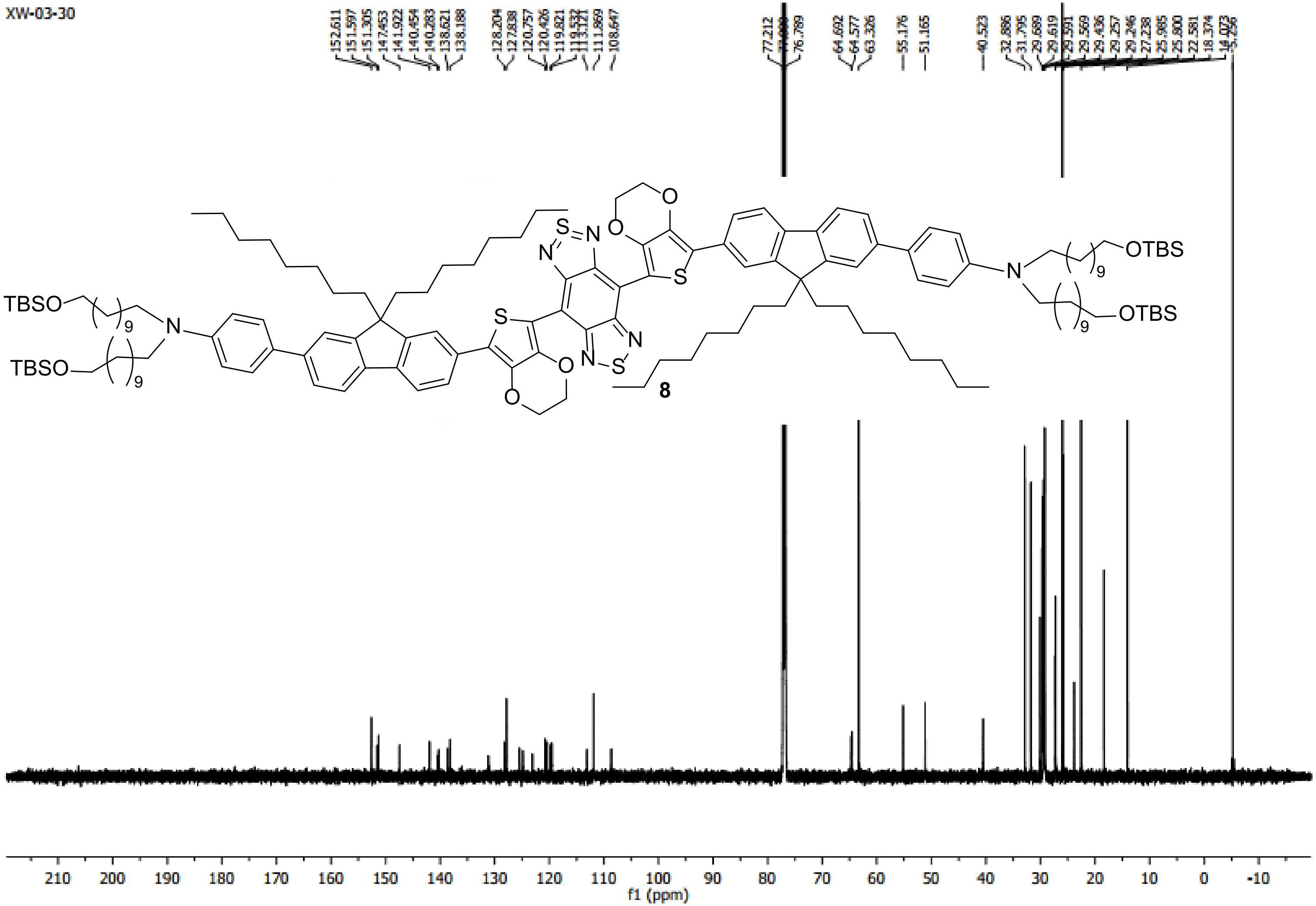

Figure S2.8. ^{13}C NMR Spectrum of Compound **8**

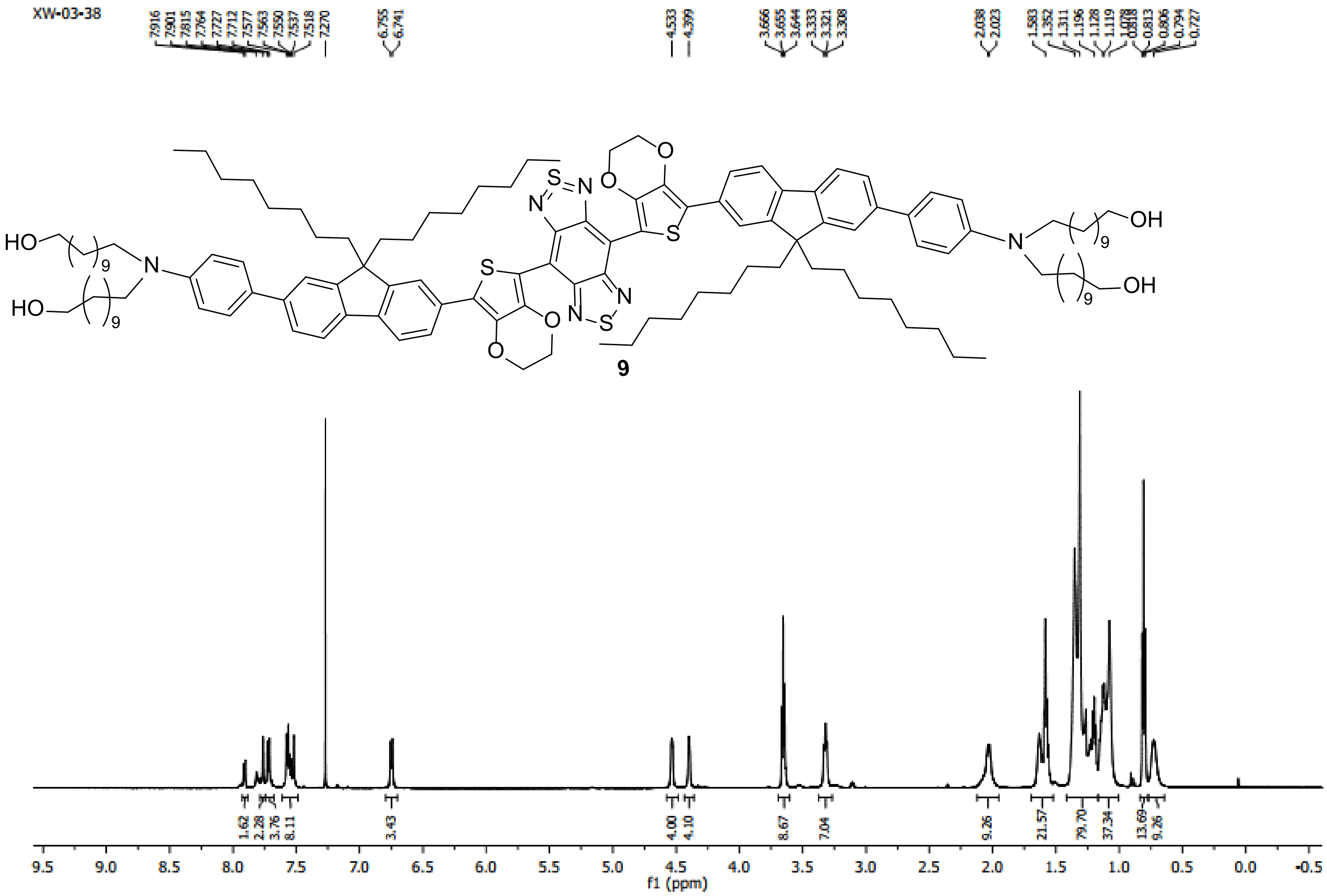

Figure S2.9. ^{1}H NMR Spectrum of Compound **9**

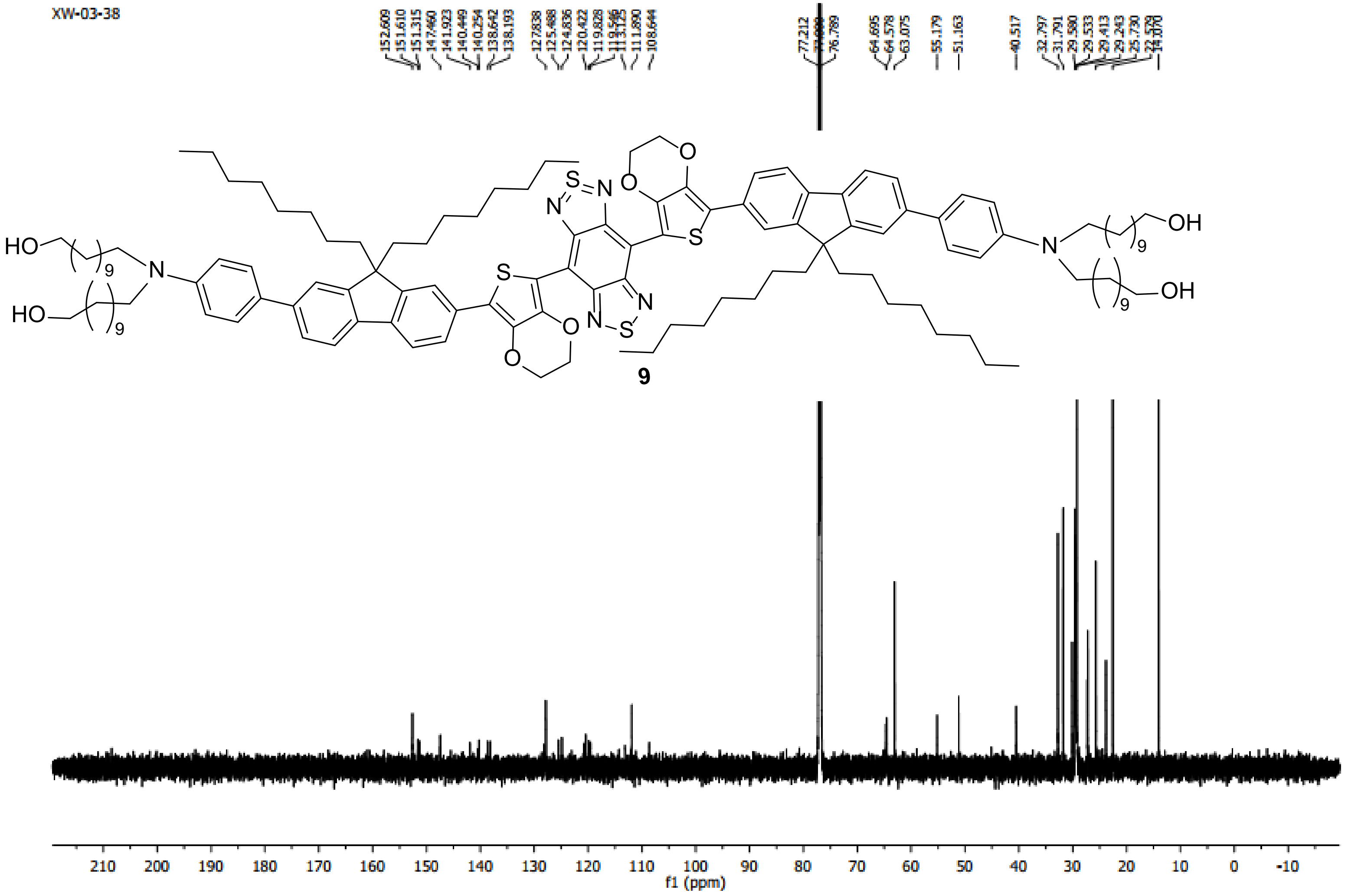

Figure S2.10. ^{13}C NMR Spectrum of Compound **9**

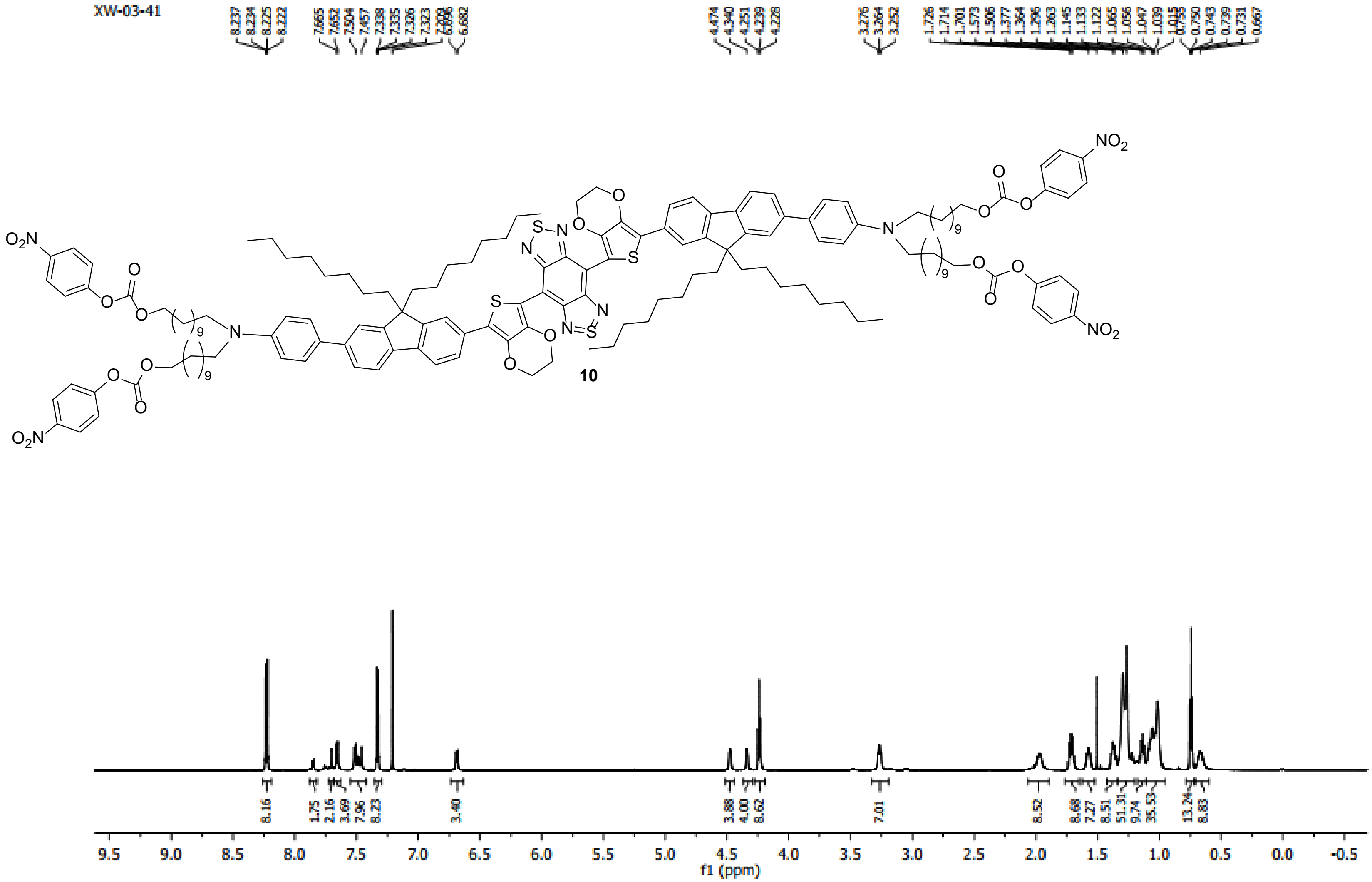

Figure S2.11. ^{1}H NMR Spectrum of Compound **10**

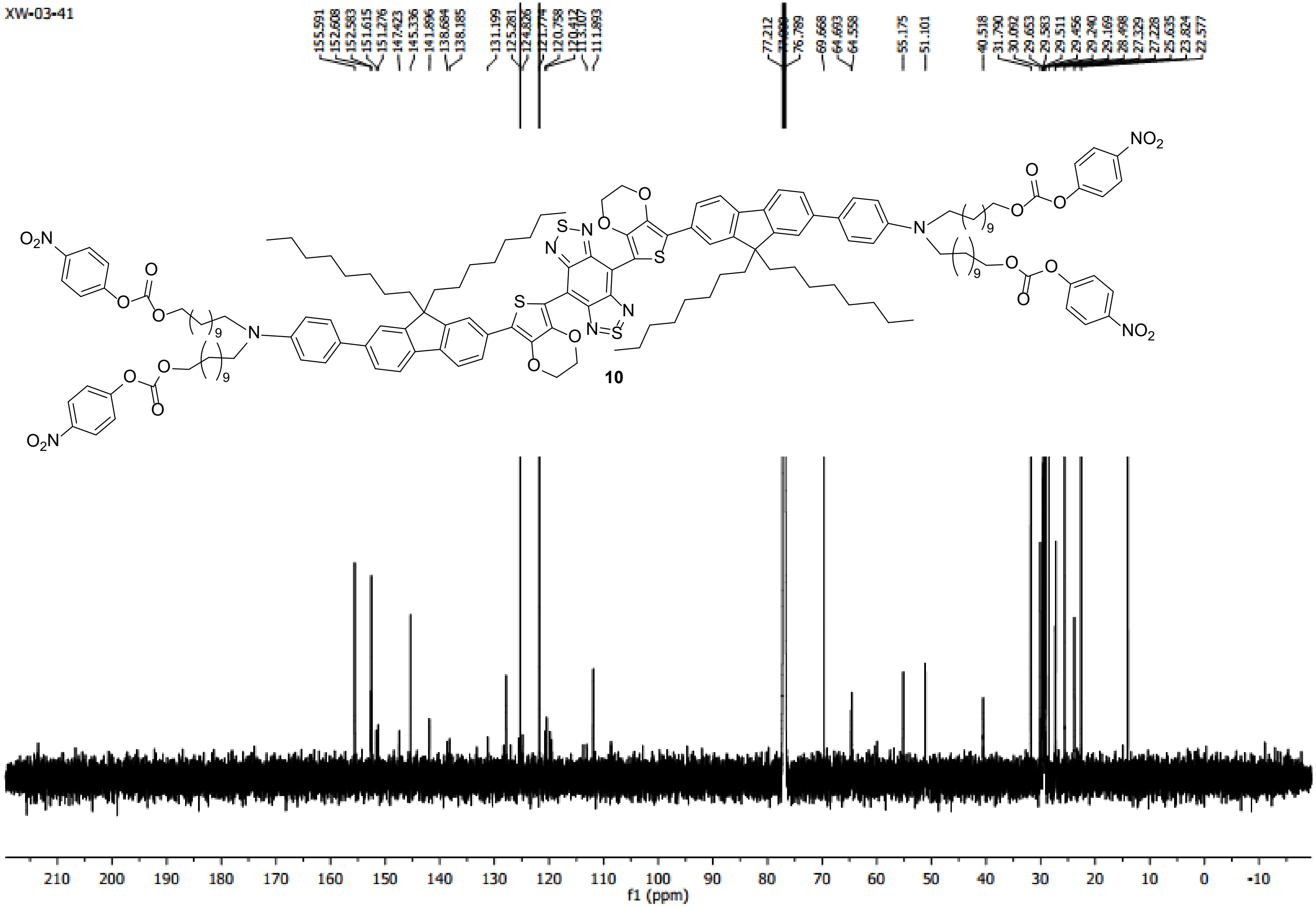

Figure S2.12. ^{13}C NMR Spectrum of Compound **10**

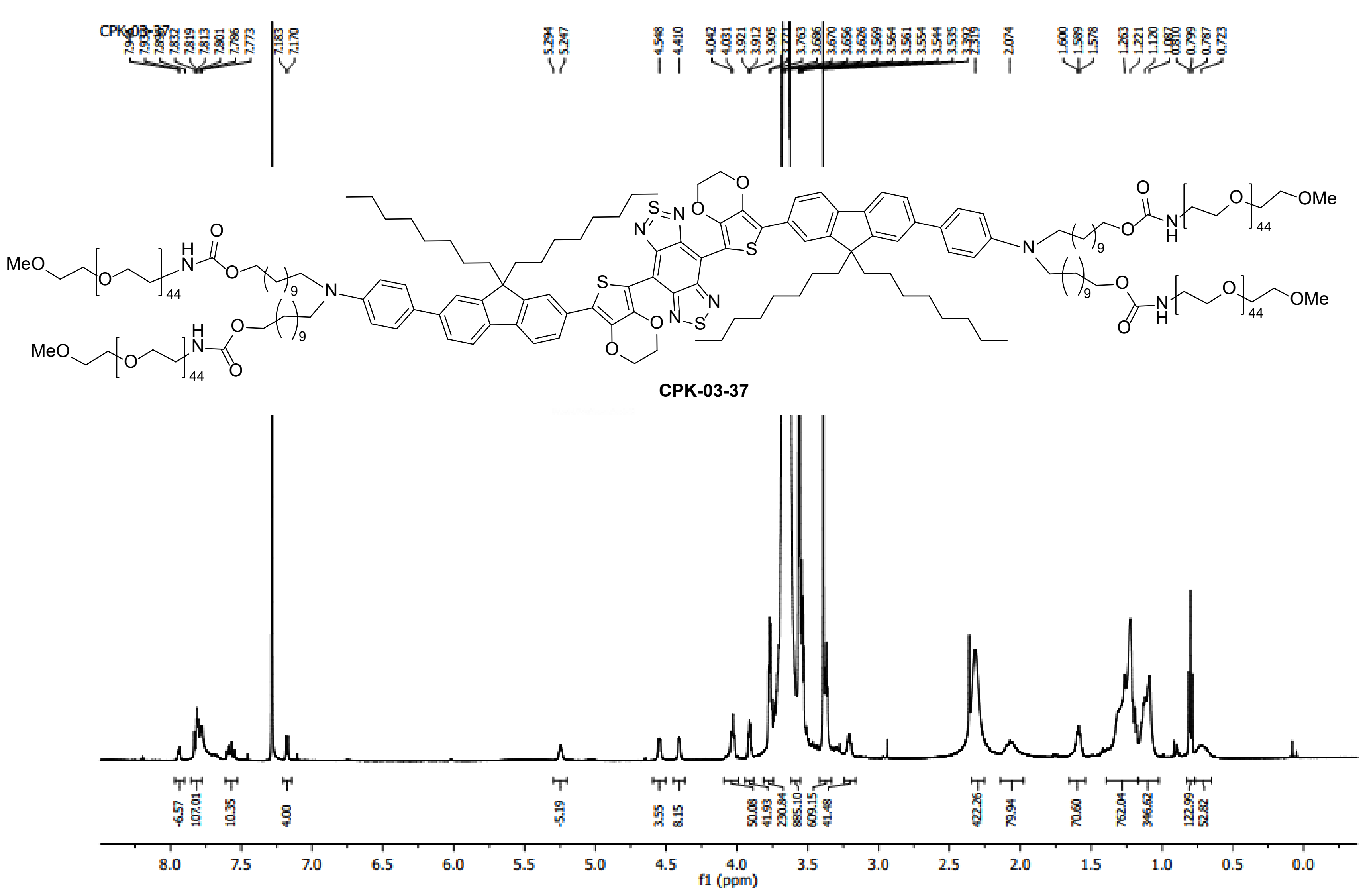

Figure S2.13. ^{1}H NMR Spectrum of Compound **CPK-03-33**

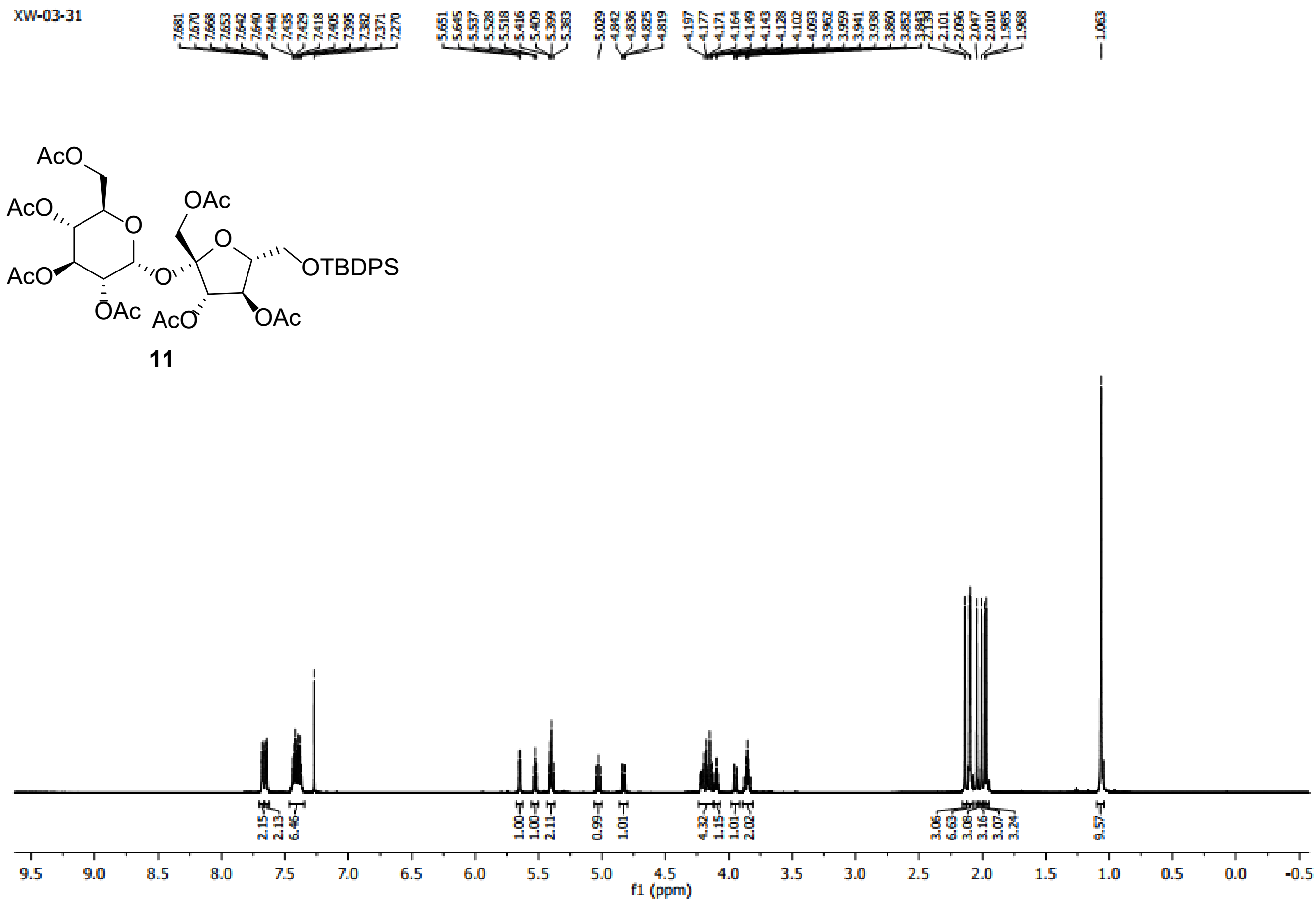

Figure S2.15. ^{1}H NMR Spectrum of Compound **11**

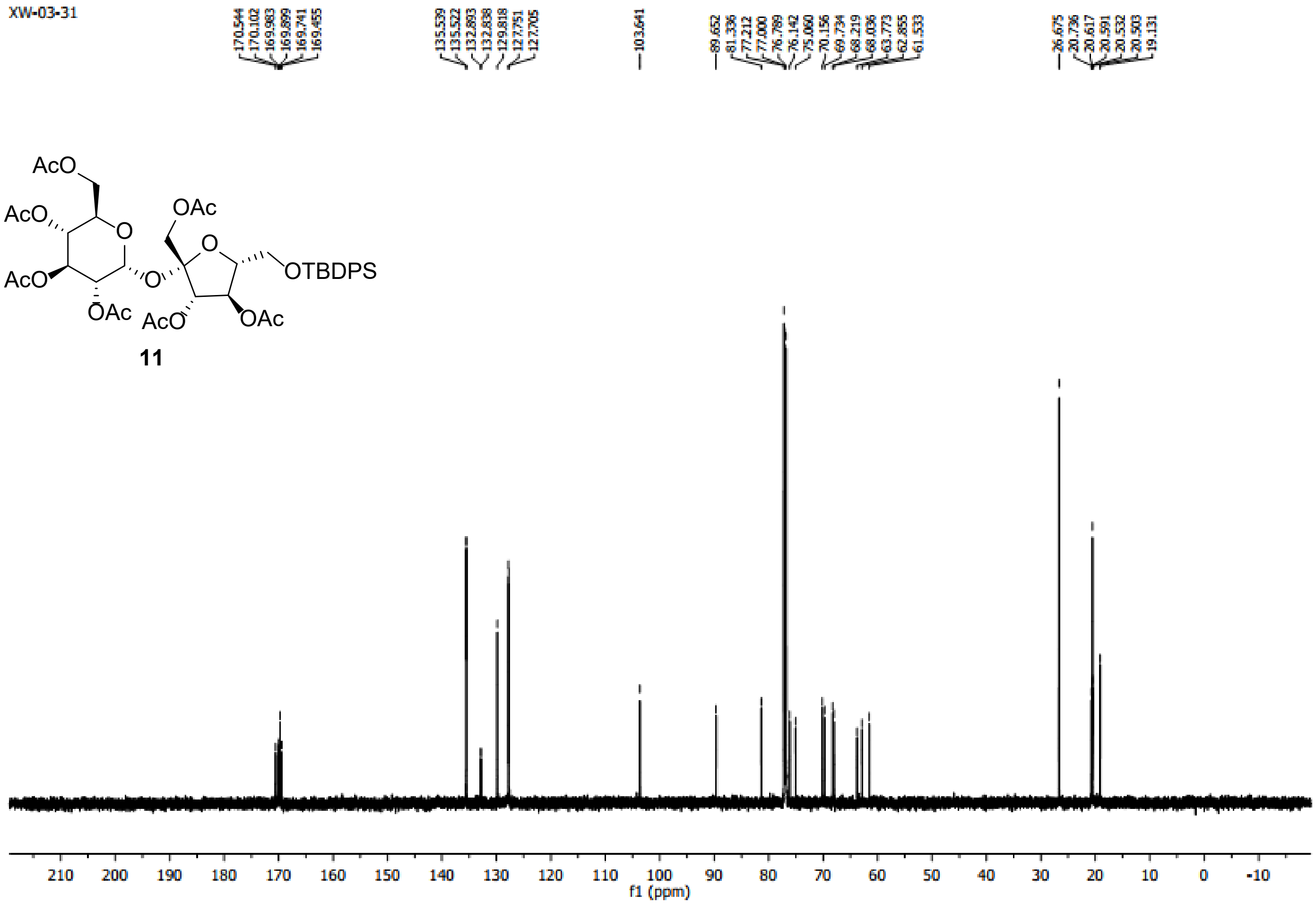

Figure S2.16. ^{13}C NMR Spectrum of Compound **11**

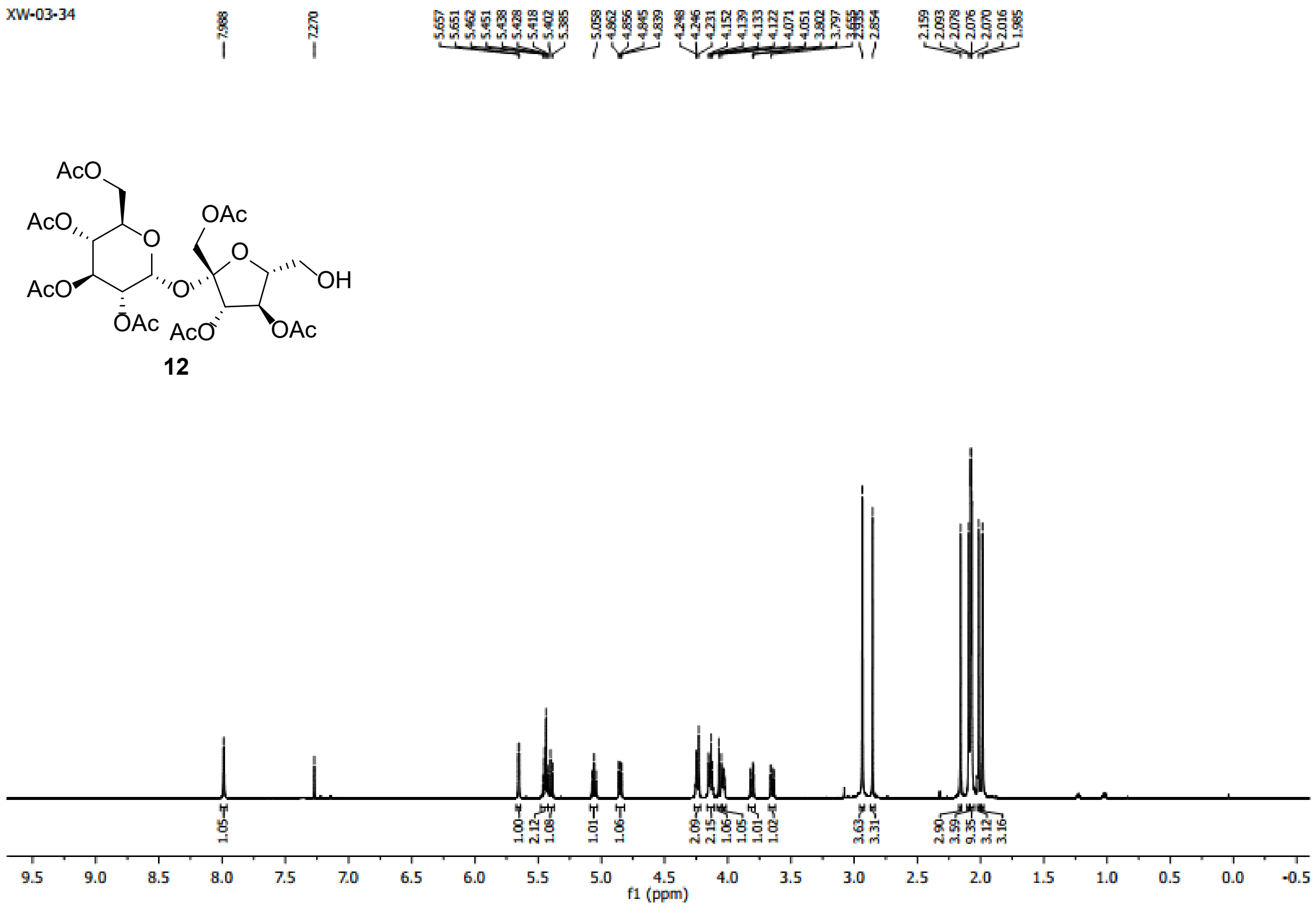

Figure S2.17. ^{1}H NMR Spectrum of Compound **12**

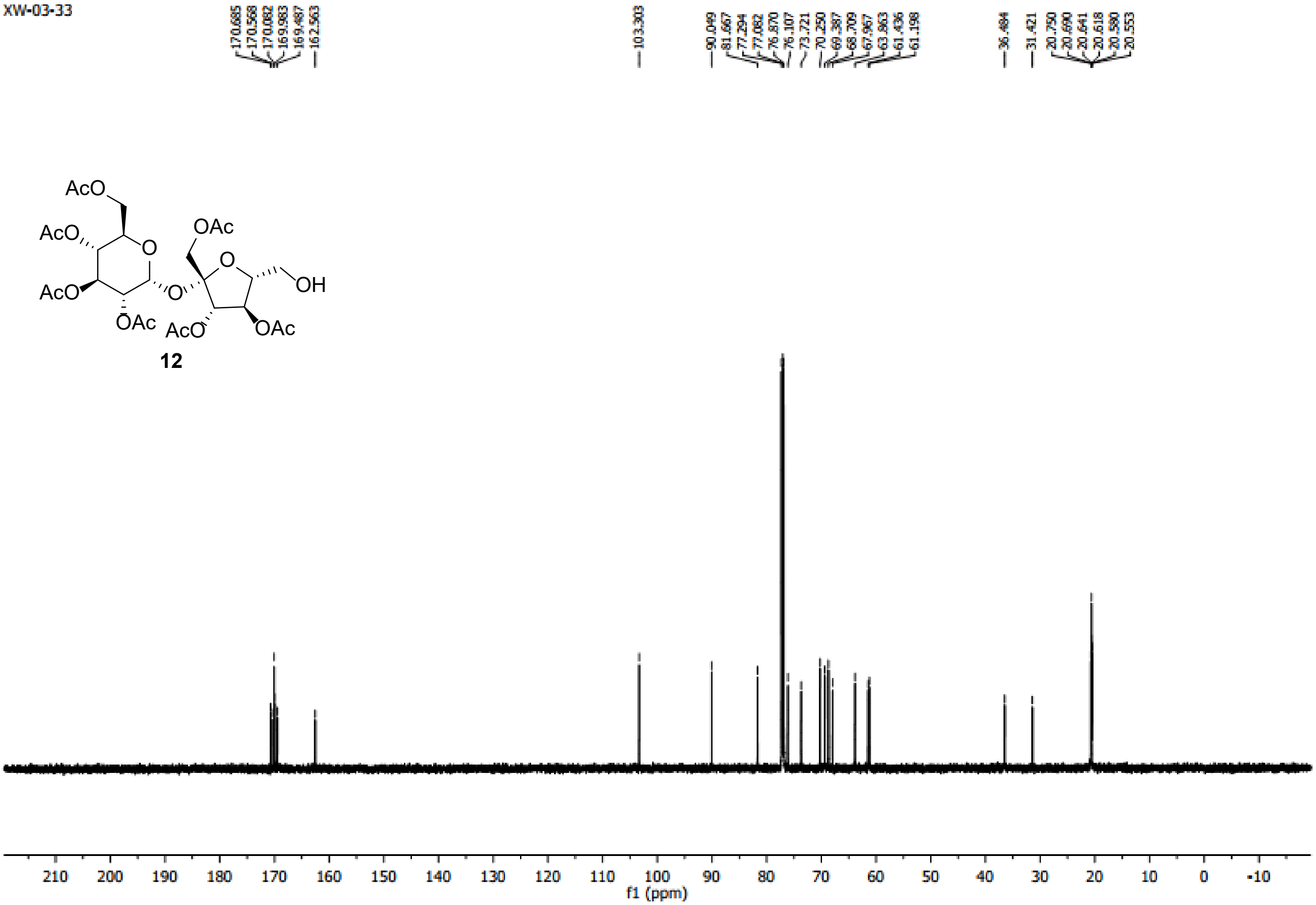

Figure S2.18. ^{13}C NMR Spectrum of Compound **12**

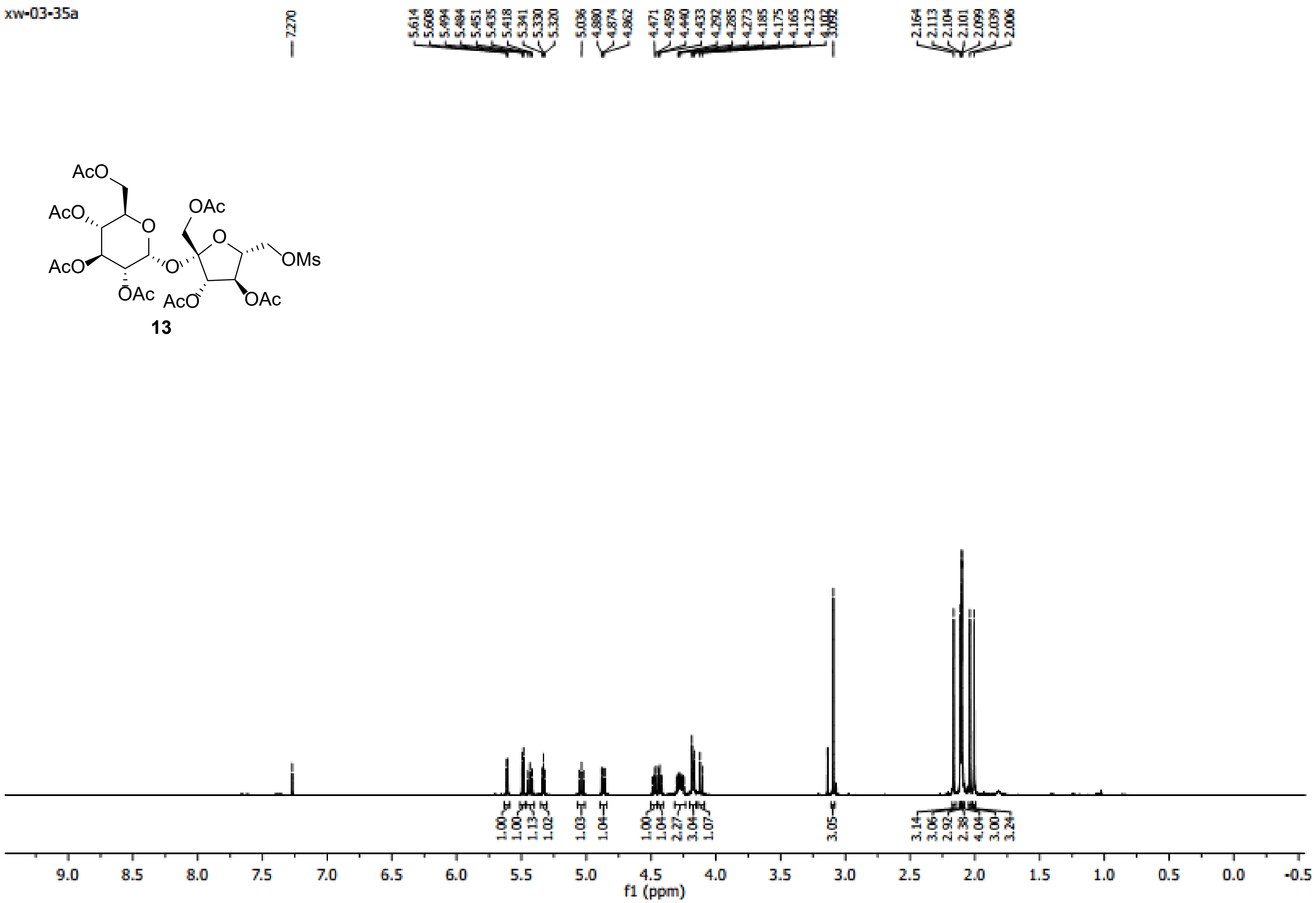

Figure S2.19. ^{1}H NMR Spectrum of Compound **13**

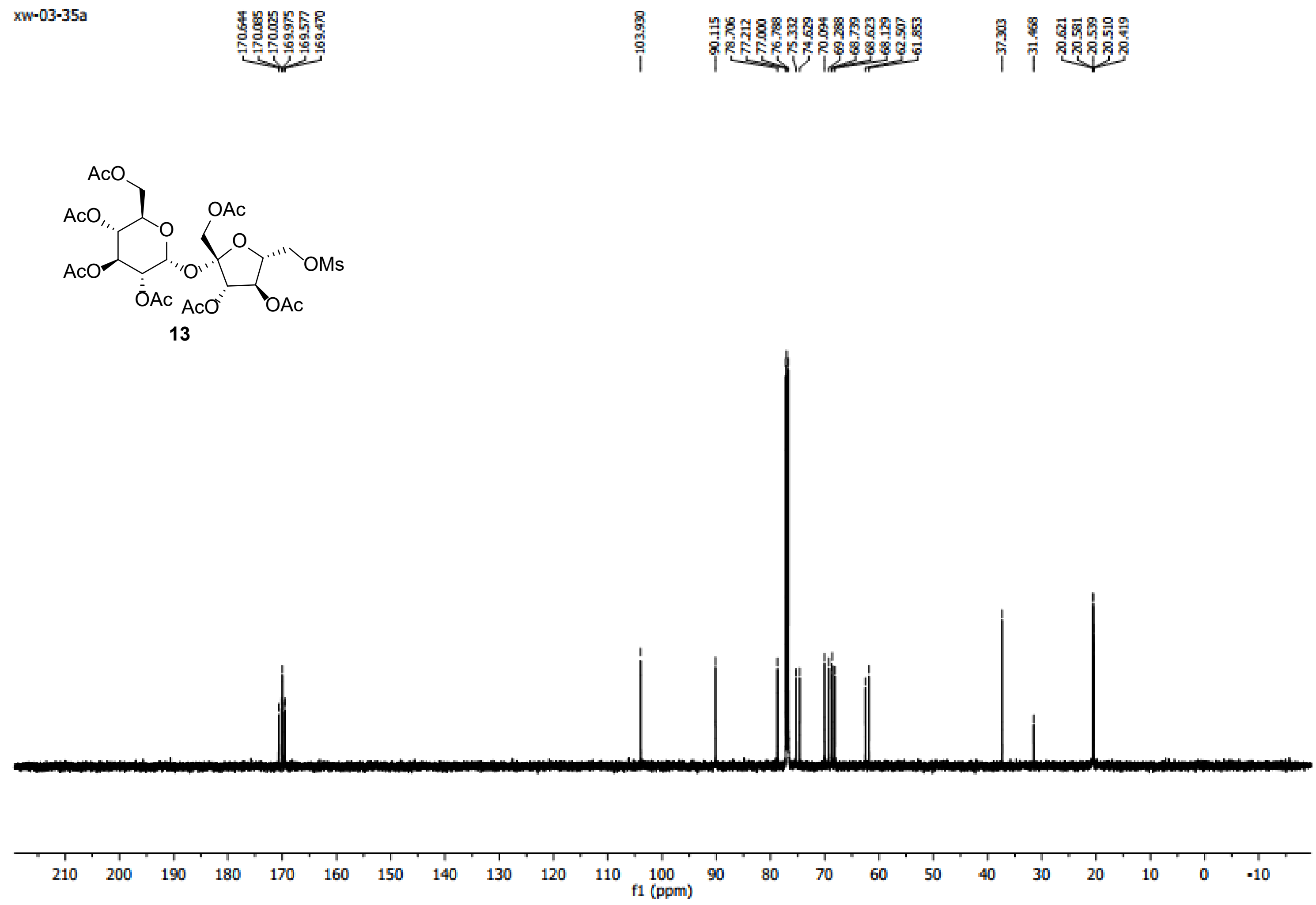

Figure S2.20. ^{13}C NMR Spectrum of Compound **13**

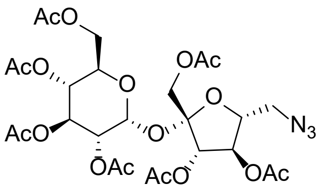

14

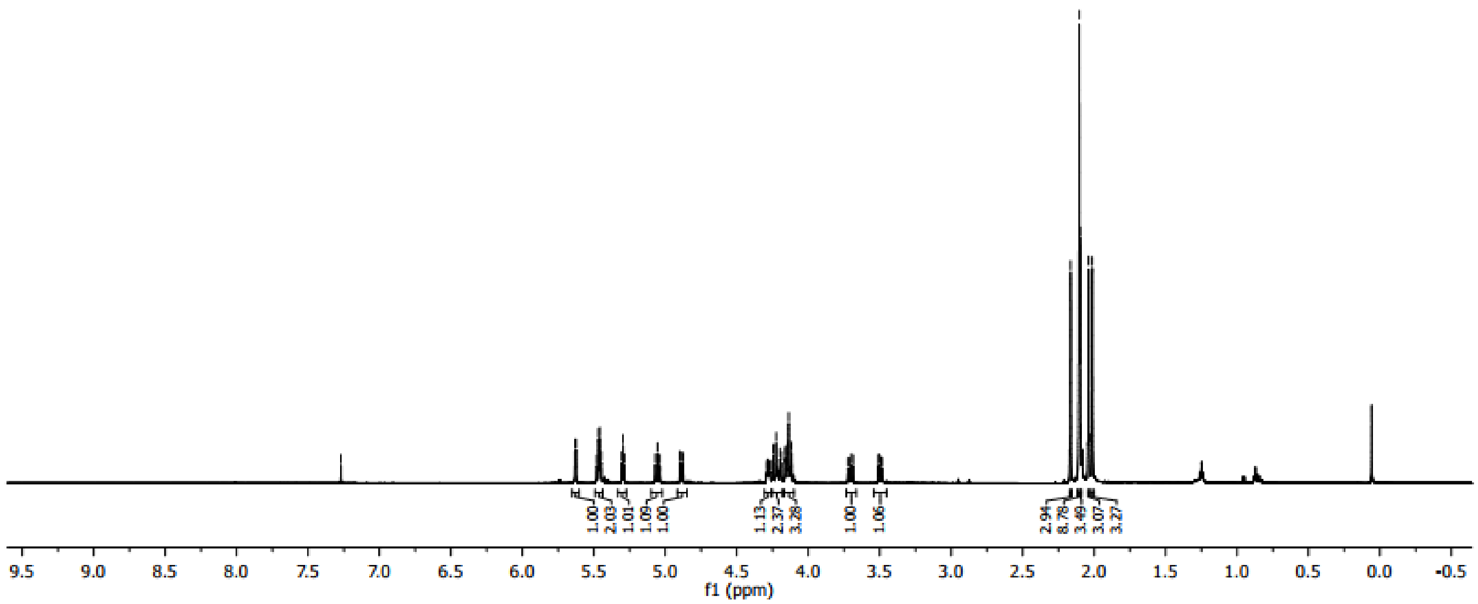

Figure S2.21. ^{1}H NMR Spectrum of Compound **14**

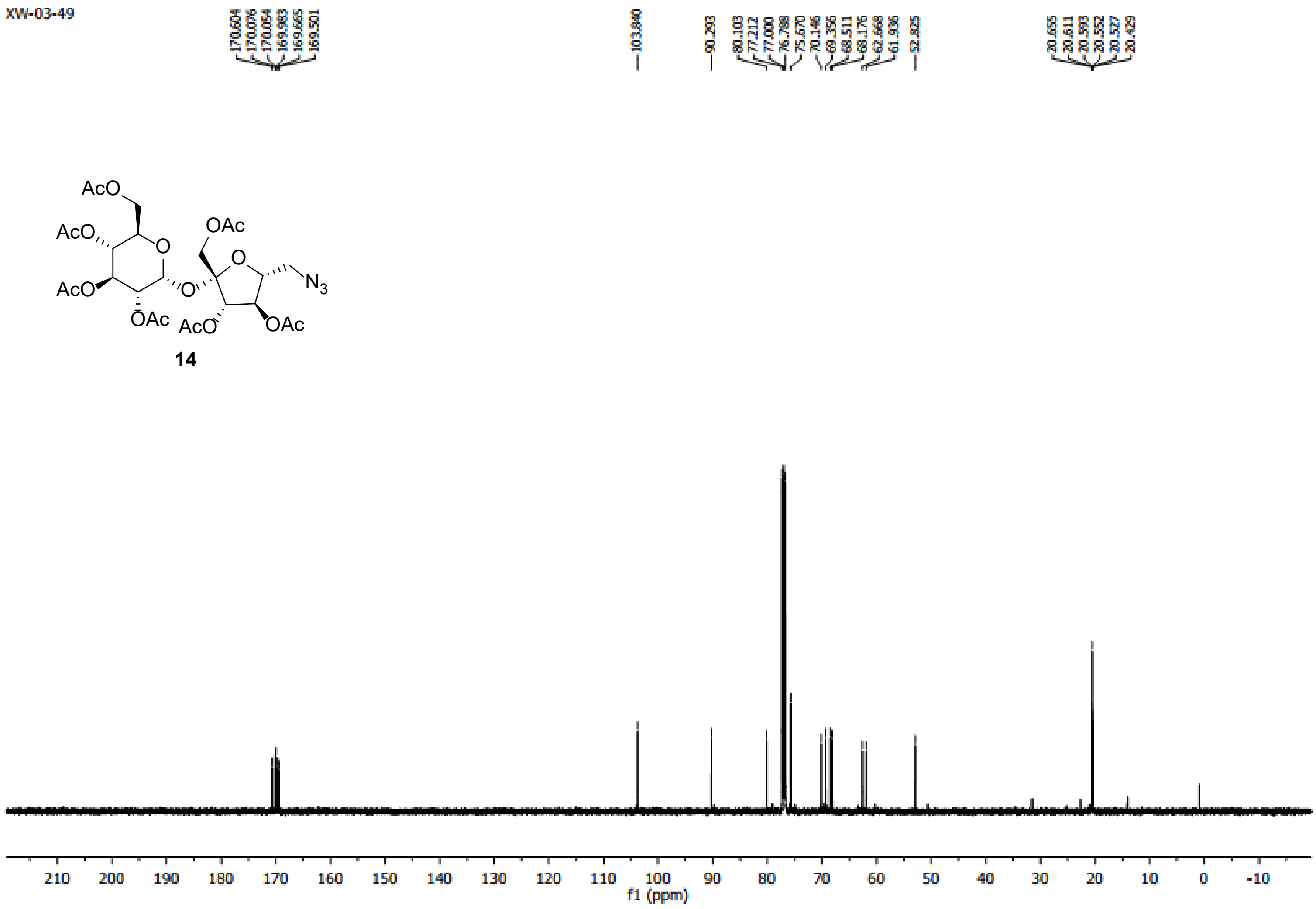

Figure S2.22. ^{13}C NMR Spectrum of Compound **14**

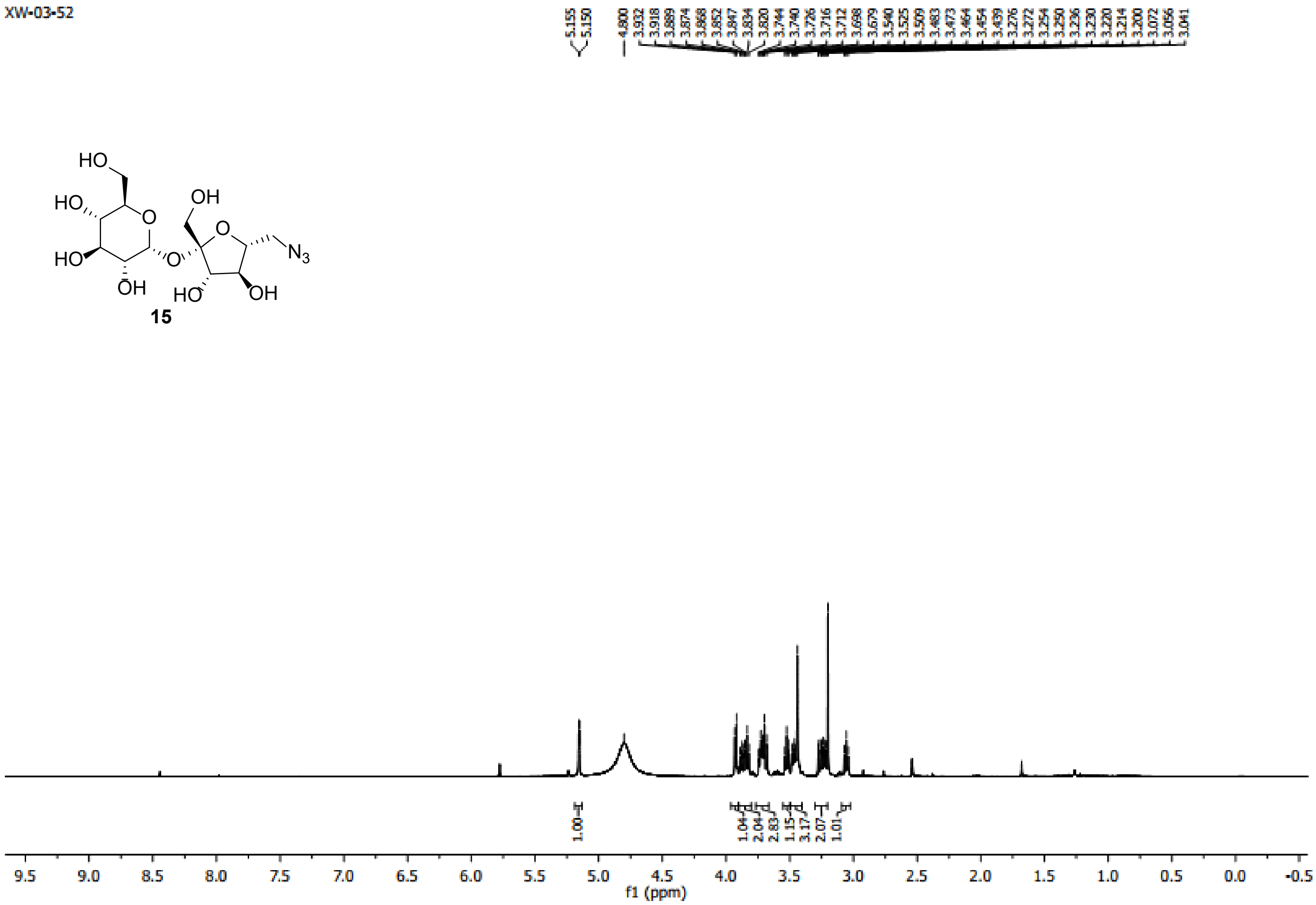

Figure S2.23. ^{1}H NMR Spectrum of Compound **15**

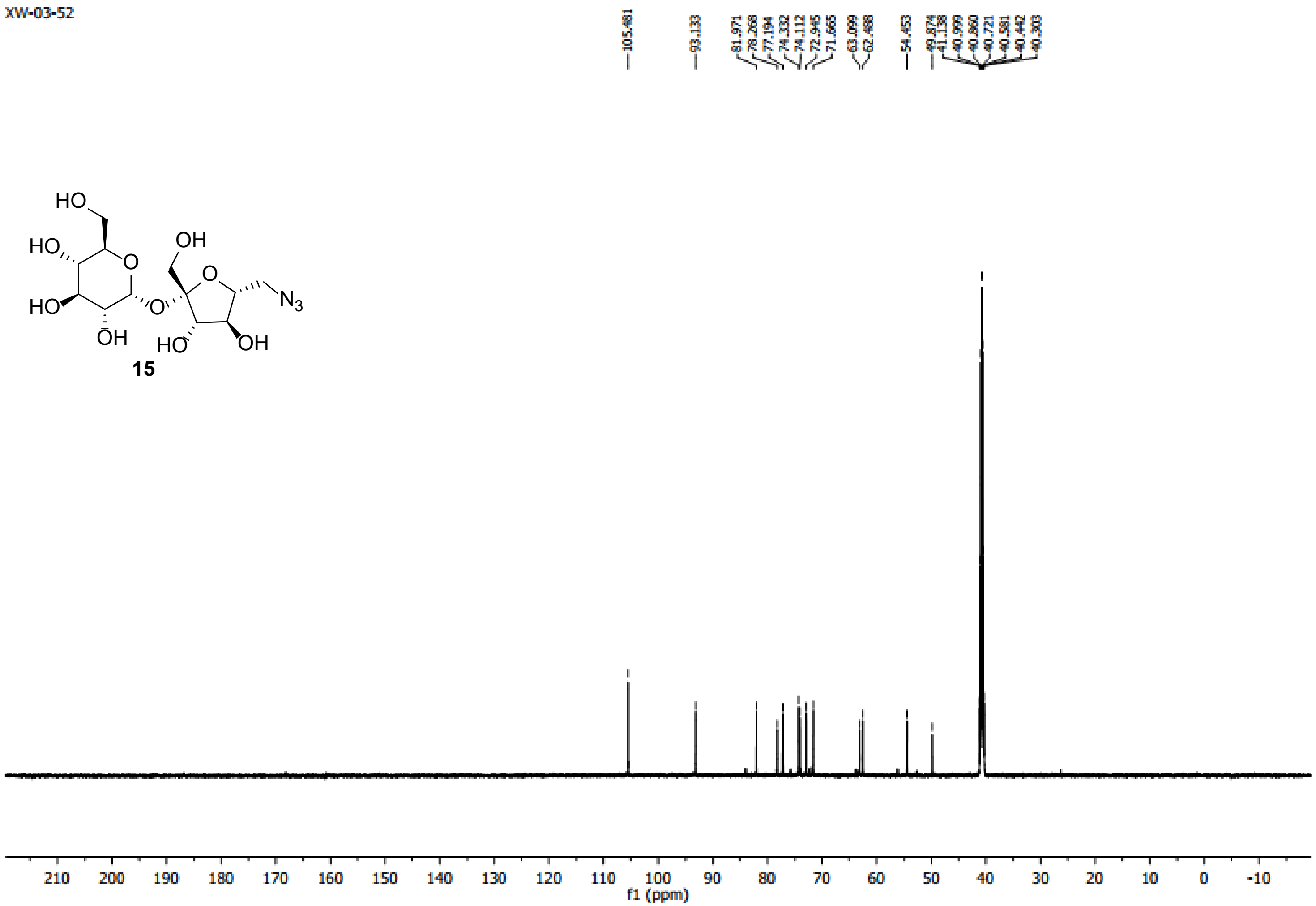

Figure S2.24. ^{13}C NMR Spectrum of Compound **15**

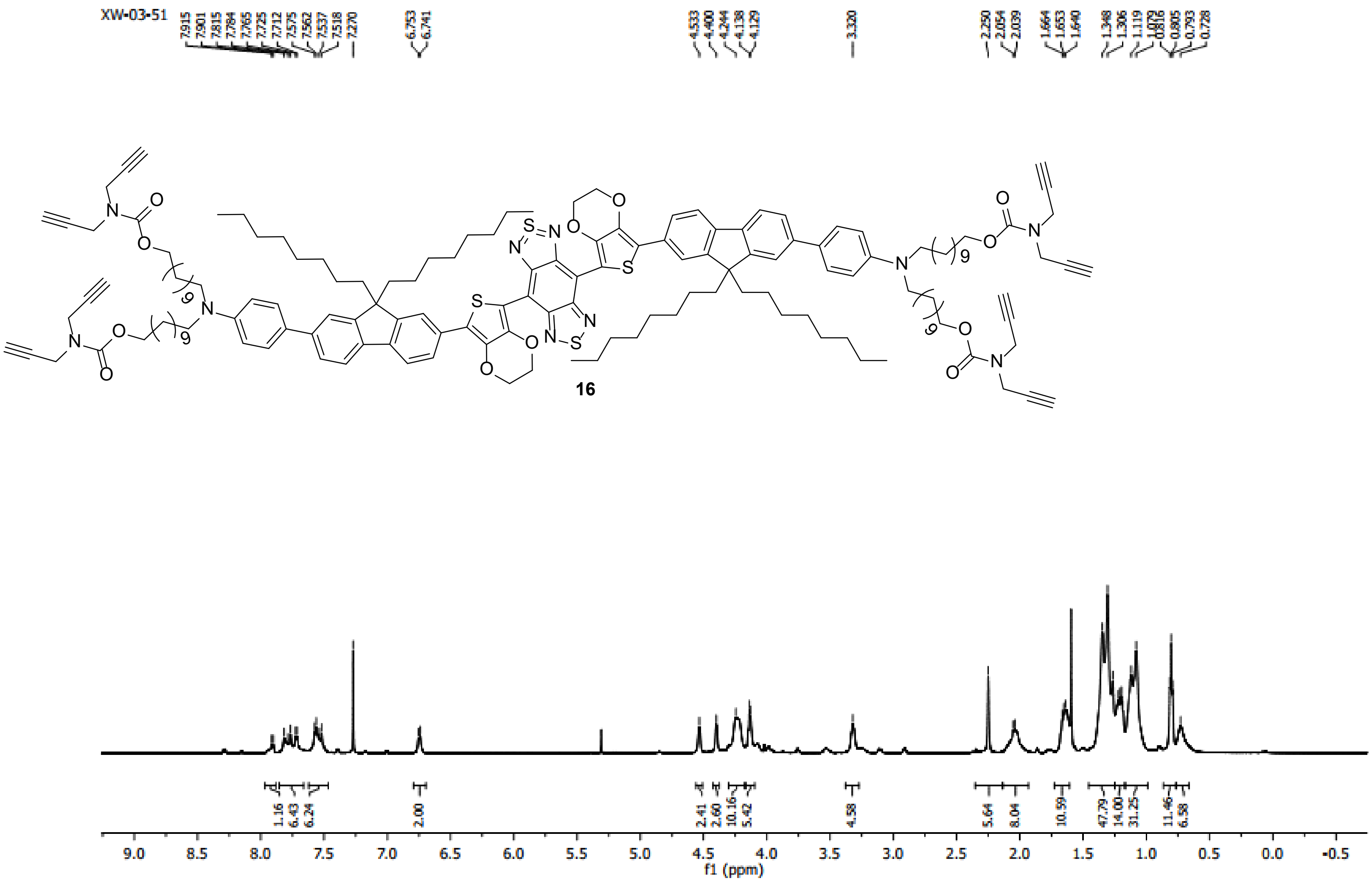

Figure S2.25. ^{1}H NMR Spectrum of Compound **16**

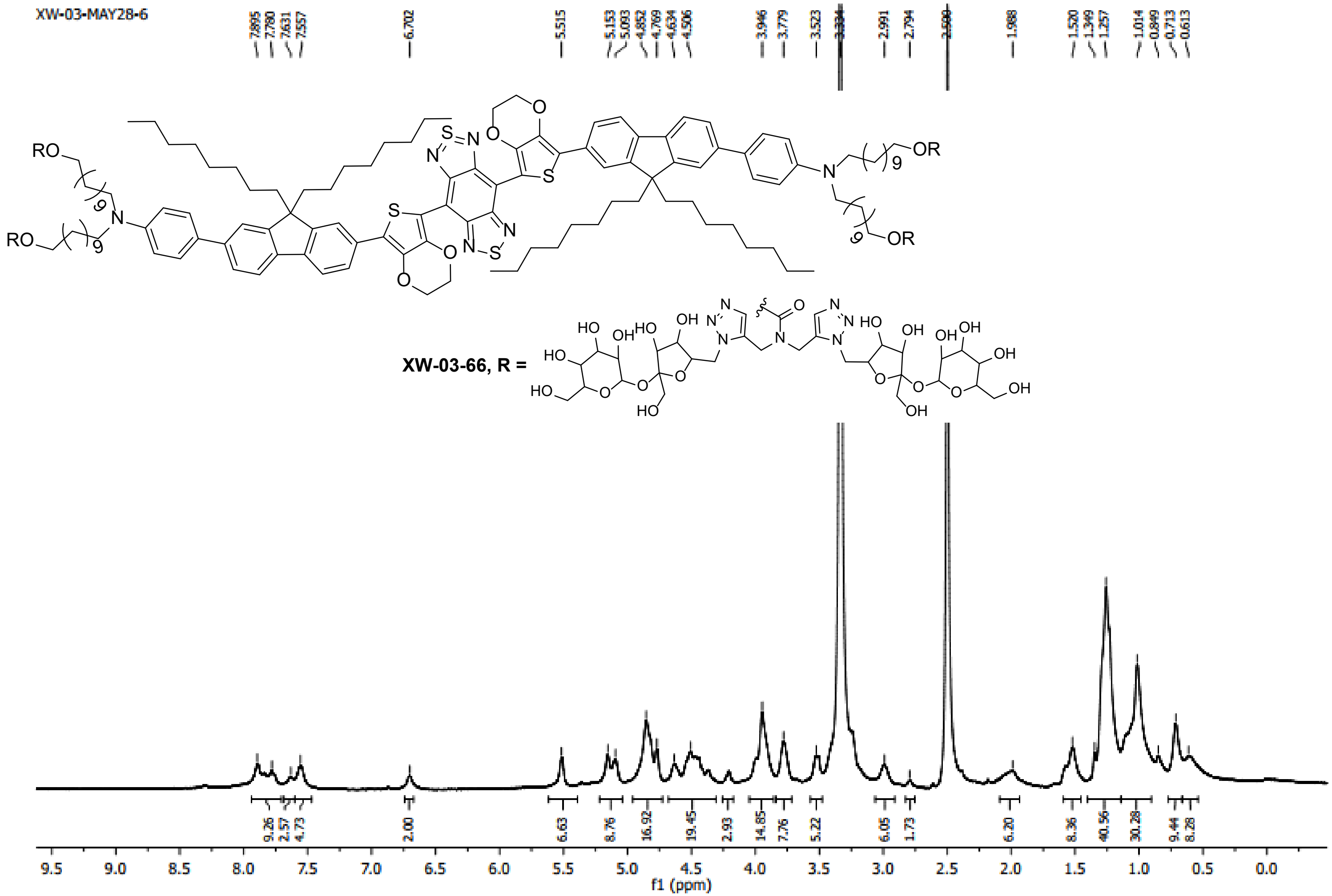

Figure S2.26. ^{1}H NMR Spectrum of Compound **XW-03-66**

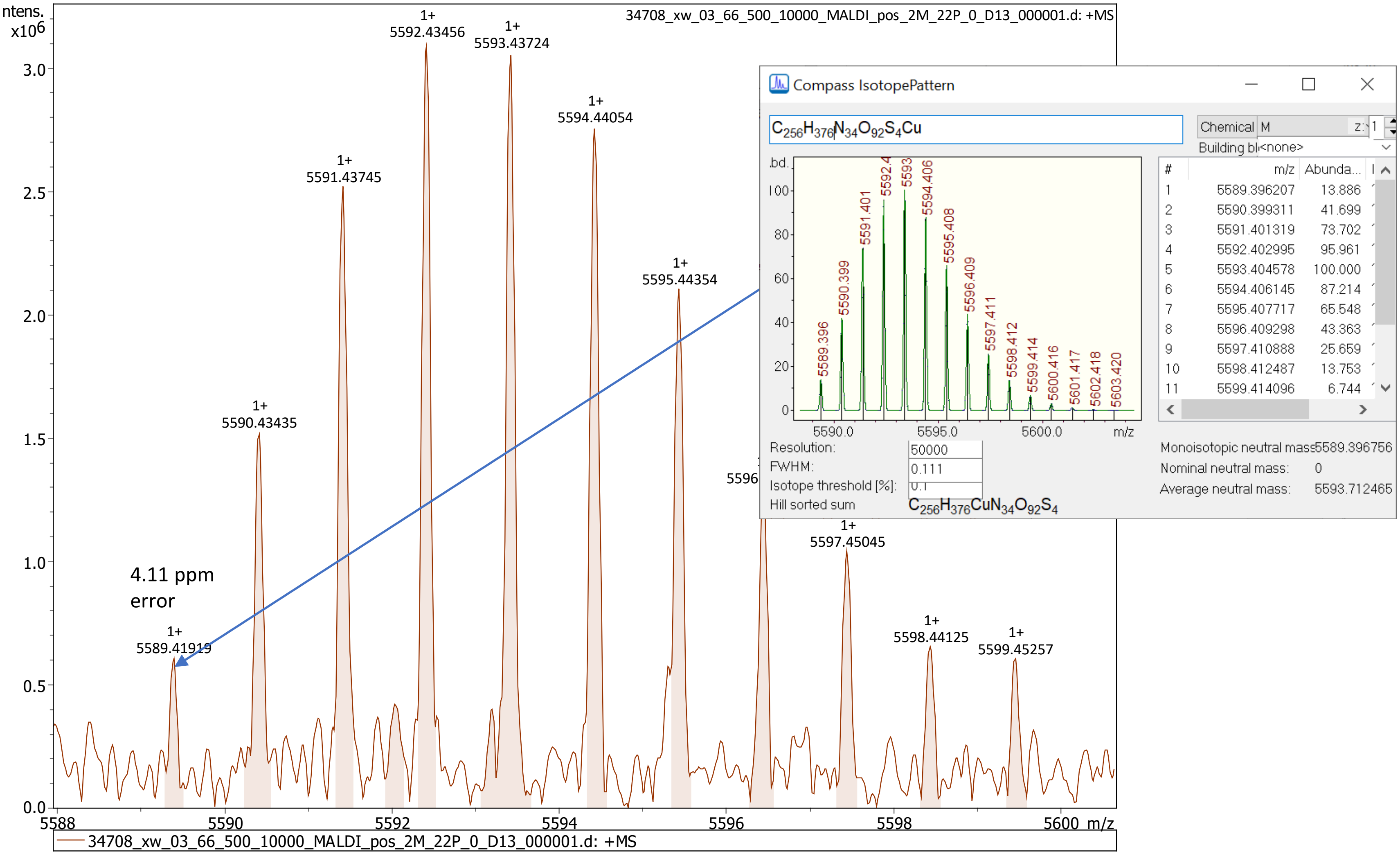

Figure S2.27. MALDI Spectrum of Compound **XW-03-66**

Data File D:\Chem32\1\Data\xianwei\XW-03-66 2022-12-03 16-00-14.D
Sample Name: XW-03-66

===

Acq. Operator   : SYSTEM
Sample Operator : SYSTEM
Acq. Instrument : LCMSD1   Location :   44
Injection Date : 12/3/2022 4:01:08 PM
                                                Inj Volume : 5.000 µl
Acq. Method   : C:\Chem32\1\Methods\Reaction-Detectionr-LCMS.M
Last changed   : 12/1/2022 4:04:06 PM by SYSTEM
                  (modified after loading)
Analysis Method : C:\Chem32\1\Methods\Reaction-Detectionr-LCMS.M
Last changed   : 12/3/2022 4:26:16 PM by SYSTEM
                  (modified after loading)
Method Info   : DON'T CHANGE THIS METHOD

Additional Info : Peak(s) manually integrated

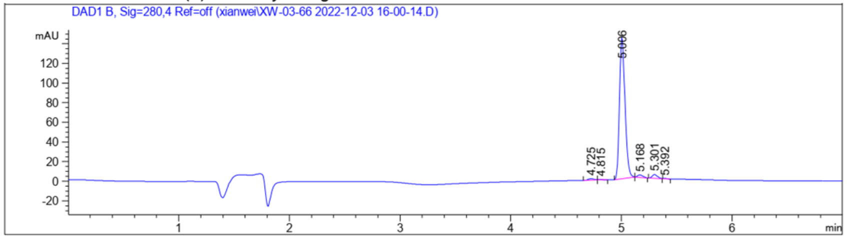

===

Area Percent Report

===

Sorted By   :   Signal
Multiplier   :   1.0000
Dilution   :   1.0000
Sample Amount: :   10.00000 [ng/ul]   (not used in calc.)
Use Multiplier & Dilution Factor with ISTDs

Signal 2: DAD1 B, Sig=400,4 Ref=off

Peak #	RetTime [min]	Type	Width [min]	Area [mAU*s]	Height [mAU]	Area %
1	4.725	BB	0.0529	4.30979	1.29386	0.8217
2	4.815	BB	0.0405	4.11902e-1	1.57341e-1	0.0785
3	5.006	BB	0.0545	499.28375	143.87720	95.1919
4	5.168	BB	0.0513	8.19625	2.56676	1.5627
5	5.301	BB	0.0489	11.83781	3.73808	2.2570
6	5.392	BB	0.0375	4.62826e-1	1.96642e-1	0.0882

Totals :   524.50233 151.82987

===

*** End of Report ***

Figure S2.28. HPLC Profile of Compound **XW-03-66**

```
Data File D:\Chem32\1\Data\xianwei\CPK-03-37 2022-12-03 16-19-15.D
Sample Name: CPK-03-37

=====================================================================
Acq. Operator   : SYSTEM
Sample Operator : SYSTEM
Acq. Instrument : LCMSD1                          Location :   42
Injection Date  : 12/3/2022 4:20:11 PM
                                                Inj Volume : 5.000 µl
Acq. Method     : C:\Chem32\1\Methods\Reaction-Detectionr-LCMS.M
Last changed    : 12/1/2022 4:04:06 PM by SYSTEM
                  (modified after loading)
Analysis Method : C:\Chem32\1\Methods\Reaction-Detectionr-LCMS.M
Last changed    : 12/3/2022 4:26:16 PM by SYSTEM
                  (modified after loading)
Method Info     : DON'T CHANGE THIS METHOD

Additional Info : Peak(s) manually integrated
```

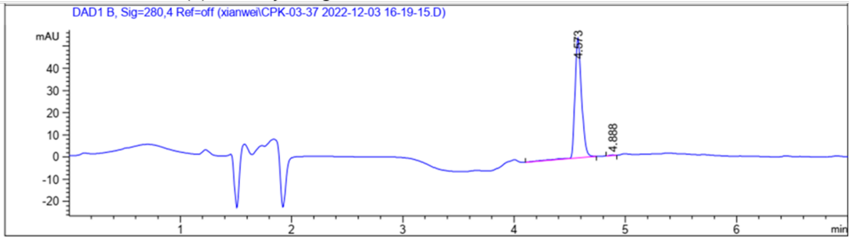


```
=====================================================================
                         Area Percent Report
=====================================================================

Sorted By             :      Signal
Multiplier            :      1.0000
Dilution              :      1.0000
Sample Amount:               :       5.00000  [ng/ul]   (not used in calc.)
Use Multiplier & Dilution Factor with ISTDs

Signal 2: DAD1 B, Sig=280,4 Ref=off
```

Peak #	RetTime [min]	Type	Width [min]	Area [mAU*s]	Height [mAU]	Area %
1	4.573	BB	0.0643	223.22112	53.94491	99.7276
2	4.888	BB	0.0512	6.09646e-1	1.91037e-1	0.2724

```
Totals :                   223.83076   54.13595

=====================================================================
                          *** End of Report ***
```

Figure S2.29. HPLC Profile of Compound **CPK-03-37**

XW-03-66 DLS (50 µM in PBS) CPK-03-37 DLS (500 µM in PBS)

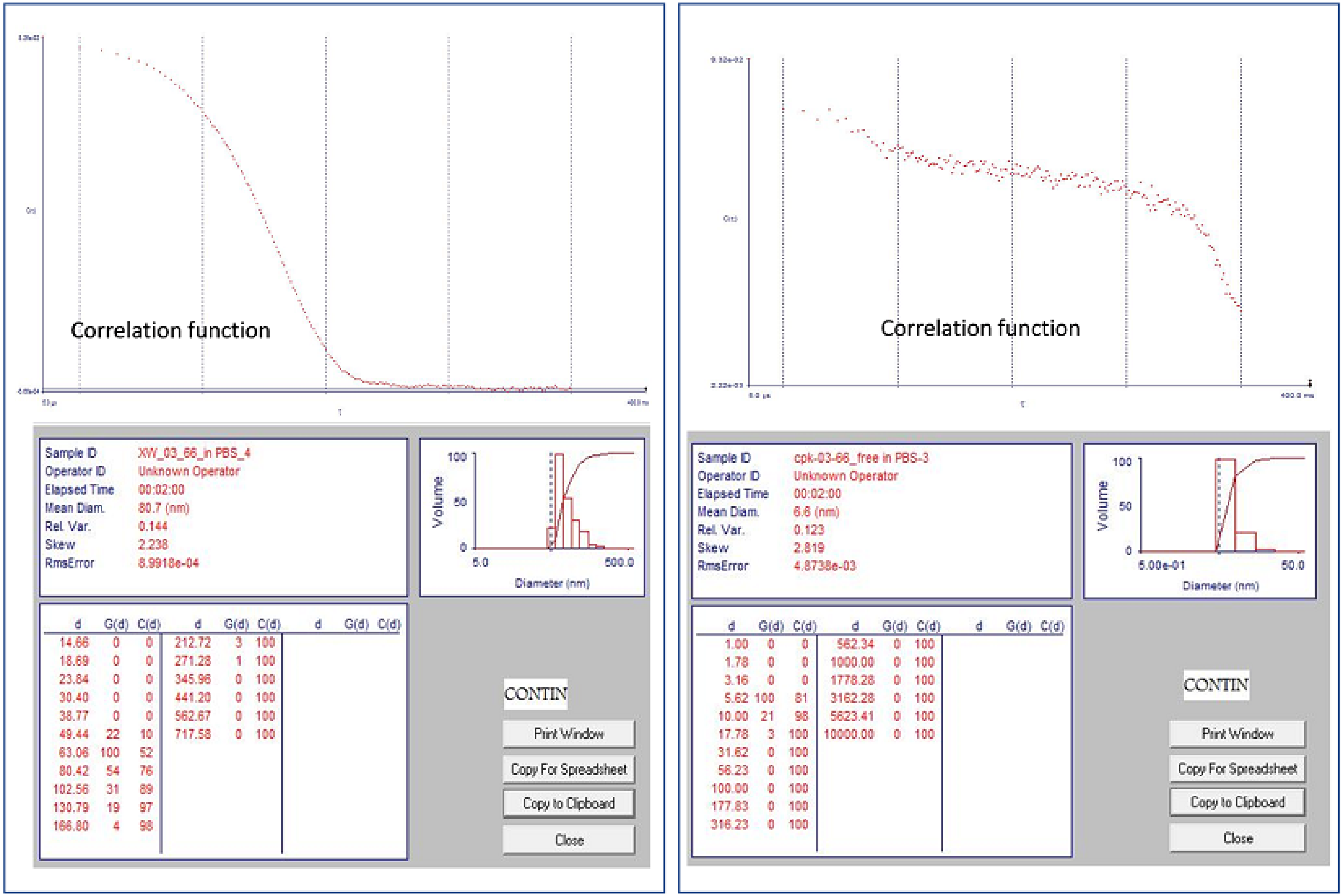

Figure S2.30. DLS Characterization of **XW-03-66** and **CPK-03-37** solutions show that unlike **XW-03-66**, **CPK-03-37** does not form particles at concentrations less than 500 µM.
